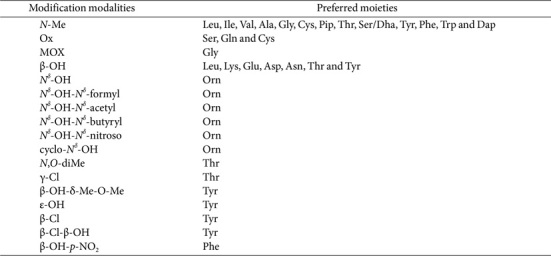# Comparative Analysis of Signature Sequences from Adenylation Domains Situated within Bacterial-Origin Nonribosomal Peptide Synthetase Modules

**DOI:** 10.4014/jmb.2503.02030

**Published:** 2025-07-14

**Authors:** Weina Gao, Zhishen Zhang, Huiying Yu, Xin Li, Chunshan Quan, Yun Xue, Pengchao Zhao

**Affiliations:** 1 College of Medical Technology and Engineering, Henan University of Science and Technology, Luoyang 471023, P.R. China; 2 Shanxi Key Laboratory of Yuncheng Salt Lake Ecological Protection and Resource Utilization, Yuncheng University, Yuncheng 044000, P.R. China; 3 Department of Life Science, Dalian Nationalities University, Dalian 116600, P.R. China

**Keywords:** α-Amino acid moiety, adenylation domain, distribution algorithm, sequence alignment, signature sequences

## Abstract

Nonribosomal peptides are assembled by large enzymes that contain multiple active sites, which function in a modular manner. The adenylation (A) domains present within typical nonribosomal peptide synthetase (NRPS) modules contain specificity-conferring codes or signature sequences (SNSs). In this study, we obtained 2051 A domain sequences from 67 bacterial species. Their alignment and clustering identified 508 SNSs. Over 80% of the SNSs displayed distinct specificity for 36 proteinogenic and nonproteinogenic α-amino acid moieties (α-AAMs). Furthermore, modifications such as *N*-methylation, monooxygenase activity, and oxidation contributed to the elongation of the A domains, while conferring pronounced affinities for certain α-AAMs. Notably, β-hydroxylation demonstrated particular preferences. Specifically, ornithine, threonine, tyrosine, and phenylalanine moieties frequently underwent atypical covalent modifications, and 41 modules were used iteratively. These insights significantly facilitate the identification of uncharacterized NRPS systems—expediting traditional identification processes—although novel modifications, unusual domain organizations, and dormant domains pose challenges for their accurate prediction.

## Introduction

Nonribosomal peptides (NRPs) constitute a prominent family of structurally diverse bacterial secondary metabolites, characterized by their brevity in that they consist of 30 or fewer residues [1]. These peptides have a remarkable potential in biotechnological and biopharmaceutical applications based on their multifaceted properties, which include antiallergic, antitumor, and immunosuppressive effects, as well as siderophore and antibiotic functions [2-5]. Their antibiotic spectrum includes activities against the bacterium *Mycobacterium tuberculosis* which causes tuberculosis, fungi, bacteria, viruses, protozoa, and even insect pests [6-11]. NRPs are synthesized by nonribosomal peptide synthetases (NRPSs), large multifunctional enzymes with a modular architecture [12-14]. Typically, the protein subunits comprise modules that process a-amino acid moieties (α-AAMs), arranging them in a linear fashion. The structural and functional parallels between NRPs and polyketides (PKs) allow NRPSs to engage with polyketide synthases (PKSs) to form hybrid assembly lines that yield novel compounds [15]. To date, an extensive array of bacterial genera—including *Bacillaceae*, *Burkholderia*, *Chondromyces*, *Cystobacter*, *Lysobacter*, *Myxococcus*, *Photorhabdus*, *Pseudomonas*, *Sorangium*, *Vibrio*, and *Xenorhabdus*—has emerged as prolific producers of NRPs and hybrid PK–NRPs [16, 17].

A quintessential NRPS module comprises an adenylation (A) domain adjacent to a peptidyl carrier protein (PCP) domain and a condensation domain, and these components collaboratively catalyze each step of the condensation process and chain elongation [18-20]. The A domain is responsible for recognizing and activating specific α-AAMs before incorporating them into the final product. Previous investigations have demonstrated the structural conservation within these domains—highlighting 10 core motifs, designated from A1–A10—and have clarified the substrate specificity inherent in the A domains [21, 22]. Notably, 10 residues located at positions 235, 236, 239, 278, 299, 301, 322, 330, 331, and 517 act as specificity-conferring codes or signature sequences (SNSs). Based on these characteristics—particularly noting that all α-AAM-activating domains possess conserved Asp235 and Lys517 residues—the putative substrates potentially recruited by A domains can be effectively predicted. Furthermore, their positions are delineated within the sequence of the NRPS subunit GrsA phenylalanine (Phe)-activating domain (consisting of 530 residues). Several NRPS services, such as NP.Searcher, SBSPKSv2, AntiSMASH, and SANDPUMA, leverage these SNSs to reflect the confidence associated with each α-AAM predicted [23-26]. Partial A domains are also endowed with under-exploited sequences situated between the consensus sequence motifs of A8 and A9. This unique positioning facilitates the modification of core NRPs with *N*-methylation (*N*-Me), monooxygenase (MOX) activity, and oxidation (Ox) [27-29].

In recent decades, many catalytic substrates of NRPSs and hybrid PKS–NRPSs have been reported. These include 20 proteinogenic and 16 nonproteinogenic α-AAMs, and among the latter are compounds such as α-aminobutyrate (Abu) [29], α-aminoadipic acid (AMA) [30], α-amino-δ-nitropentanoic acid (ANPA) [31], azetidine α-carboxylic acid (AZC) [32], β-cyano-alanine (Cya-3) [33], α-γ-diaminobutyric acid (Dab) [34], a,β-diaminopropionic acid (Dap) [35], dehydro-α-aminopropanoic acid/dehydroalanine (Dha) [36], dehydrobutyrine (Dhb) [37], dehydrovaline (Dhv) [38], enduracididine (End) [39], homoarginine (Har) [40], γ-hydroxy-phenyl glycine (Hpg) [41], homoserine/homoserine lactone (Hse/Hsl) [42], ornithine (Orn) [43], and pipecolic acid (cyclic lysine) (Pip) [8]. However, many existing SNSs cannot precisely identify these compounds. Notably, many silent biosynthetic gene clusters (BGCs) related to NRPSs and PKS–NRPSs have been identified with genomic sequencing and bioinformatic methodologies—indicating a variability in SNS matches [7, 44-47]. In this study, we examined the SNSs associated with published A domains together with their respective substrates, which were categorized based on their similarities, and especially examined indistinguishable SNS profiles for atypical modifications, unconventional domain organizations, and inactive domains. Our finding extend our understanding of NRPS systems and suggest innovative frameworks with which to decode their biosynthetic intricacies.

## Materials and Methods

### Data Sources

In total, 321 NRPS and PKS–NRPS sequences were sourced from recognized databases, including the National Center for Biotechnology Information (NCBI, http://www.ncbi.nlm.nih.gov/), the *Pseudomonas* Genome Database (https://www.pseudomonas.com/), and AntiSMASH (https://antismash.secondarymetabolites.org/). These data included a diverse range of substrates, bacterial sources, and biological evaluations (Table S1). Of these sequences, we screened representatives from 67 bacterial species-approximately two-thirds of whose genomes have now been sequenced.

### Determination of A Domain Sequences and SNSs

We used ClustalX version 1.81 and NCBI BLAST to construct intricate multiple alignments of the downloaded NRPS and PKS–NRPS sequences against the GrsA Phe-activating domain, culminating in the identification of 2051 A domain sequences. Ultimately, multiple analytical tools-including PKS/NRPS Analysis website (http://nrps.igs.umaryland.edu/), AntiSMASH, NCBI BLAST, ClustalX, and SnapGene (USA) - were used for the acquisition of the SNS data.

### Multiple Alignment of SNSs

In an exhaustive analysis involving 36 substrates, a total of 508 SNSs clustered together. Appropriate α-AAMs displaying highly homologous SNSs were aggregated according to their occurrence frequencies. The sequences within each group were aligned with Excel 2010 and ClustalX, and their subsequent examination was based on observed similarities.

## Results

### Leucine-, Ioleucine-, Valine-/Dehydrovaline-, and γ-Hydroxyphenyl-Glycine-Activating Domains

Table S2 shows that among the 36 substrates examined, leucine (Leu), isoleucine (Ile), and valine (Val)—including its modified derivative, Dhv—were represented most abundantly, cumulatively with 525 occurrences (n).

A comprehensive analysis of 224 Leu-activating domains, 104 Ile-activating domains, and 197 Val-activating domains detected a total of 117 SNSs. The majority of these motifs were significantly conserved, conferring structural similarities (Tables S3−S5). However, they were still distinguishable. The most commonly encountered codes included DAWFLGNVVK, DAMFLGCTYK, and DALWIGGTFK (Table 1). This demonstrates that Leu-activating domains had the most-conserved SNS profiles. Furthermore, a group of 17 SNSs showed marked prevalence. It is noteworthy that approximately two-thirds of these SNS occurrences were represented across various species—illustrating the inherent sequence diversity within three distinct A domains (Tables S3−S5).

The phenomenon of substrate promiscuity associated with identical SNS patterns is further highlighted in Table 2. It is evident that Ile shares greater homology within its SNS structures with Val than with Leu, implying a closer structural relationship between Ile and Val, despite their classification as isomers. Furthermore, six notable sequences are expected to display considerable flexibility towards *N*-Me and β-hydroxylated (β-OH) moieties (Table 3). Simultaneously, DAWCIGAVCK, DLYNLSGVWK, and DAMHLGCTFK/DILHLGCTFK also manifest a capacity to accommodate Phe, Ala, and Hpg moieties, respectively. The sequence DAWWIGGTFK, derived from the Dhv-activating domain, is not identical to the sequences found in Val-activating domains, but impressively shares 90% homology with sequences such as DALWIGGTFK, DAWFIGGTFK, and DAWWLGGTFK.

### Alanine-/α-Aminobutyrate-, Methionine-, Glycine-, and Cysteine-Activating Domains

In this study, we identified 155 alanine (Ala)-activating domains distributed across 73 distinct assembly lines (Table S2). Among the 46 SNSs examined, five carried two specific residues at positions 239 and 330 (*n* = 101; Table 4). The overall analysis identified the predominant variability, but six sequence clusters shared ≥ 80%identity. Furthermore, three sequence pairs accommodated serine (Ser), Abu, and glycine (Gly) moieties. Notably, DVFYLGGVFK, DVFYLGGVCK, and DVWYLGGICK incorporated methionine (Met) moieties, and two of them shared ≥ 70% homology with DVFWLGGTFK within the Ala-activating domain.

Concurrently, within the 72 megasynthases identified, we detected 93 Gly-activating domains (Table S2). Of these, 12 SNSs demonstrated remarkable (70%) homology, distinguished by three distinctive residues at positions 299, 322, and 330 (*n* = 83; Table 5). Furthermore, two sequence pairs shared 90% identity. MOX is a specialized domain that cleaves the carbon backbone inherent in Gly moieties. It is particularly noteworthy that four Gly-activating domains showed MOX characteristics and all shared the identical sequence DILQLGMIWK.

Moreover, 42 megasynthases included 74 cysteine (Cys)-activating domains (Table S2). Remarkably, a predominant cluster comprising 17 homologous SNSs seems to have emerged through recombination events between Ala- and Gly-activating domains (Table S8). Residues Asp235, Leu236, Tyr/Trp239, and Asn278 trace back to the origins of the Ala-activating domain, whereas Leu/Met299, Leu/Met322, Ile/Val330, Trp331, and Lys517 derive from Gly-activating domains. Obviously, five of these have garnered considerable attention (*n* = 55). In addition, DLYNLSLIWK, DLYNMSLIWK, DLWNLSLIWK, DLYNLALVWK and DLYNWSLIWK catalyzed Ox modified moieties.

### Ornithine-/α-Amino-δ-Nitropentanoic Acid-, Lysine-/Cyclic Lysine-, Proline-/Azetidine α-Carboxylic Acid-, and Arginine-/Enduracididine-/Homoarginine-Activating Domains

It is noteworthy that 147 Orn-/lysine (Lys, including Pip)-activating domains were distributed across 71 assembly lines (Table S2). Remarkably, ten SNSs derived from Orn-activating domains showed substantial divergence, with only 70% identity (Table 6). Fifteen SNSs had the capacity to accommodate modifications on the Orn moiety—specifically *N*^δ^-hydroxylation (*N*^δ^-OH), *N*^δ^-acetylation (*N*^δ^-acetyl), *N*^δ^-formylation (*N*^δ^-formyl), *N*^δ^-butyrylation (*N*^δ^-butyryl), and *N*^δ^-nitrosylation (*N*^δ^-nitroso). With exceptions such as DGEACGGVTK, DGECTGGITK, DGEGSGGVTK, and DVWNIGLIHK, these sequences can be readily distinguished from one another. Furthermore, six specific sequences were extended through an innovative modification involving *N*^δ^-OH-Orn, subsequently undergoing cyclization into stable six-membered rings via the closure of the thioesterase domain. Consequently, these variants are predominantly situated at terminal positions. DMEDVGSVDK and DVETLGGISK also demonstrated an ability to assemble Lys and ANPA moieties, respectively.

Equally remarkably, among the 58 Lys-activating domains examined, 13 SNSs were detected with notable frequency (*n* = 39; Table 7). Four sequences may facilitate Lys cyclization (*i.e.*, Pip) between the N-terminal and ε-amino groups. Furthermore, two sequence pairs are representations of Pro and Arg moieties, respectively. Subsequent interaction analyses revealed that the sequence DGEDHGTVVK shares 90% identity with its counterpart DGEDHGTVTK, derived from Orn-activating domains.

We also established that 71 proline (Pro)-activating domains were distributed across 60 assembly lines (Table S2). Notably, 25 SNSs containing identical residues—specifically Val236 and Gln239—were frequently observed (*n* = 59; Table S11). Almost 90% of these instances have been grouped, with each showing 80% identity (Table 8). Three other sequences showing 70% identity were also identified. It is important to note that both sequences DVQCLSEVTK and DMQLVSQQVK have evolved separately to accommodate AZC.

Thirty-one megasynthases collectively featured 37 arginine (Arg)-activating domains (Table S2). Although 29 SNSs were utilized infrequently, the majority showed a significant clustering tendency, including Asp235, Ala/Val236, Glu239, Asp278, Ile/Val/Leu299, Gly301, Ala322, Val/Ile330, Thr/Asp331, and Lys517 (Table S12). Furthermore, the sequences DIGDLGIIDK and DAEDVAAMIK were distinctly linked to α,β-dehydro-Arg and End, respectively, and feeding experiments confirmed their origin from Arg. Both showed degrees of clustering exceeding 70%, highlighting the intrinsic challenges associated with modifying the Arg moiety [48]. Notably, DVESIGGVTK showed particular versatility by also accommodating the Har moiety, suggesting structural similarity between them.

### Glutamate-/Glutamine-, Aspartic Acid-/Asparagine-, β-Cyano-Alanine-, and α-Aminoadipic Acid-Activating Domains

We established 227 glutamate (Glu)-/glutamine (Gln)-activating domains and aspartic acid (Asp)-/asparagine (Asn)-activating domains positioned across 108 assembly lines (Table S2). Among the delineated Glu-/Gln-activating domains, 21 SNSs featured prominently (*n* = 95; Table 9). It is evident that the sequence DAQDLGVVDK recognizes both Glu and Gln moieties.

Similarly, an analysis of Asp-/Asn-activating domains revealed the presence of 24 SNSs (Table S2). Remarkably, both domains showed an identical sequence DLTKVGHVGK (Table 10). However, most sequences favored the Asp moiety (Table S15). Moreover, 16 SNSs were characterized with *n* = 100. Notably, two-thirds of Asn-activating domains displayed 80% identity (Table 10). Further investigations indicated that various sequences can accommodate β-OH-Asp/-Asn moieties, implying strong amenability for modification within the Asp moiety. We also discovered that two sequence clusters from Asp- and Glu-activating domains shared ≥ 80% homology, and both Asn- and Gln-activating domains shared the identical sequence DAVQMGCVDK. Collectively, these findings suggest a cross-linkage among four distinct A domains—a phenomenon that may present slight challenges to their precise differentiation. DLTKIGEVGK is also compatible with the Cya-3 moiety, whereas DPRHLALLAK, specific for the AMA moiety, showed 80% identity with its counterpart DPRHVSLLAK, derived from Asn-activating domains.

### Threonine-/Dhb-Activating Domains

A comprehensive analysis revealed that 213 threonine (Thr)-/Dhb-activating domains occurred within 127 assembly lines (Table S2). One-third of the SNSs that recognize both the Thr and Dhb moieties were characterized by two distinct residues located at positions 278 and 299 (*n* = 205; Table S16). Three other sequences—DMFCAGLIWK, DMFSAGLIWK, and DMFVAGLIWK—showed remarkable flexibility towards the Thr moiety, and shared 90% similarity, whereas DFWNIGMVHK, DFWSVGMVHK, DMFNFGVLWK, and DMFCNGIIWK also showed significant selectivity for *N*-Me-/*N,O*-diMe-, γ-Cl-, β-OH-Thr, and serine (Ser) moieties, respectively.

### Serine-/Dehydroalanine- and Homoserine/Homoserine Lactone-Activating Domains

We identified 201 Ser-/Dha-activating domains-characterized by identical sequences DVWHLSLVDK and DVWHMSLVDK-arranged across 132 assembly lines (Tables S2 and S17). Ten SNSs contained two distinct residues at positions 299 and 330 (*n* = 191), four of which were also specific for Ox and *N*-Me modifications. DLKNVGSDVK and DLKNLGTDVK were also identified within the Hse/Hsl-activating domains, frequently accompanied by occurrences of Ser/Dha (Tables S1 and S17).

### Tyrosine-, Phenylalanine-, and Tryptophan-Activating Domains

The 169 domains responsible for the activation of tyrosine (Tyr)-, Phe-, and tryptophan (Trp) were distributed across 114 assembly lines (Tables S2). Among these, seven SNSs derived from Tyr-activating domains displayed 70% similarity (Table 11). Homology of ≥ 80% was also observed among two distinct sequence clusters. Particularly noteworthy was the affinity shown by various sequences toward modifications such as β-OH, *N*-Me, ε-OH, β-Cl, β-Cl-β-OH, and β-OH-δ-Me-O-Me.

Substantial variability was detected in the pool of 54 Phe-activating domains. However, the frequency of SNSs remained relatively low (n £ 5; Tables S2 and S19), eight of which had been grouped (Table 12). High homologies, up to 90%, were observed among two distinct sequence pairs. Four sequences recognized *N*-Me and β-OH-*p*-NO_2_ modifications, and 11 SNSs from Trp-activating domains were classified based on their ≥ 80% homology (*n* = 19; Table S20).

Modified Tyr and Phe moieties were activated with greater facility, paving the way for unconventional substrates to emerge. However, the corresponding sequences displayed only ≤ 60% identity, suggesting that novel moieties may be introduced through intricate modifications, concurrently altering SNSs. An identical sequence, DVSAIGCVTK, was present within these three distinct A domains (Table 11). In pairwise alignments between them, the Phe- and Trp-activating domains showed four identical sequences, which significantly surpassed the number shared between the Tyr- and Phe-activating domains, or between the Tyr- and Trp-activating domains (Table 12). This observation confirms the strong association between Phe and Trp moieties during the chain elongation processes.

### α,γ-Diaminobutyric Acid, α,β-Diaminopropionic Acid-, and Histine-Activating Domains

The 86 Dab-activating domains were integrated into 43 assembly lines, seven of which were classified (*n* = 82; Tables S2 and 13). Notably, DVWQMIGDDK specifically recognizes the β-OH modification (Table 13). Intriguingly, two sequences to accommodate Dap moieties shared 80% identity with those derived from Dab-activating domains. This observation underscores the notable similarities between the Dab and Dap domains, although they are probably differentiated by subtle but significant variations.

Finally, four SNSs were incorporated within 14 His-activating domains. Remarkably, the first two shared 90%homology and appeared most frequently (*n* = 11; Table S21). It is noteworthy that DSALIAEVWK can accept the β-OH modification. A multiple-sequence alignment revealed their weak homology with other α-AAMs, allowing the precise identification of His moieties.

Based on the genetic findings outlined above, over the past 2 years, NRPS services have shown that the genomes of various bacteria have BGCs encoding known NRPSs, including *Bacillus amyloliquefaciens* MR14M3 [49], *Bacillus velezensis* NDB [50], *Bacillus halotolerans* AQ11M9 [51], *Bacillus paralicheniformis* PBl 36 [52], and *Bacillus subtilis* BS21 and PBs12, together with 13 isolates of *Xenorhabdus* and *Photorhabdus* (Table S22) [53, 54]. They have also demonstrated genetically and biochemically that BGC5 (*Paenibacillus brasilensis* KACC 13842), *corA* (*Corallococcus exiguus* SDU70), *crz123456* (*Corallococcus coralloides* B035), *mgpABCDEF* (two *Burkholderiales* strains), *selAB* (*Burkholderia* sp. FERM BP-3421) and *solFGH* (*Dickeya solani* MK10) orchestrate novel NRPs and PK–NRPs, including bracidin [55], coralinone [56], corallorazines [57], megapolipeptins [58], selethramide [59], and solanimycins [60]. The predicted α-AAMs align remarkably well with those characterized with traditional methods, such as liquid chromatography–mass spectrometry (LC–MS), LC–MS/MS, and nuclear magnetic resonance (NMR).

## Discussion

This study demonstrates that bacterial genera contain a strong diversity of NRPS and PKS–NRPS chains. An exhaustive investigation into 2051 A domain sequences revealed the existence of 36 proteinogenic and non-proteinogenic α-AAMs, each characterized by its specific SNSs (roughly two thirds are shown in Table 14). It is clear that these motifs are frequently used. However, 89 SNSs have a demonstrated capacity to accommodate two or more α-AAMs, highlighting the complexity involved in their recognition. Distinguishing noncanonical moieties, such as Hpg, Abu, ANPA, Pip, AZC, Har, Cya-3, Dhb, and Dha, from their parent or structurally analogous proteinogenic α-AAM counterparts is a formidable challenge given their indistinguishable SNS profiles. Among these atypical compounds, Hpg—a rare phenylglycine—has been shown to play significant roles in structural stabilization [41, 61]. ANPA represents an unusual nitro α-AAM, which is exceedingly scarce in nature. Its biosynthesis involves the NRPS subunit ornithine MOX (MbaC), which facilitates *N*-OH conversion, whereas unprotected hydroxylamine undergoes spontaneous Ox to yield nitroso and nitro derivatives [31]. Pip features a hydrazine N–N bond formed through the action of piperazate synthase together with Pro. Together they impose conformational constraints on peptides-essential for pharmaceutical activities [62]. Although AZC has been detected in certain plant species, it remains relatively uncommon among bacteria [63]. Its biosynthetic pathway shows that azetidine originates from the intramolecular cyclization of Met-derived S-adenosylmethionine-a process akin to the synthesis observed in plant nicotianamine. The existence of two identical SNSs between AZC and Pro moieties opens new avenues for investigating the structural diversity and pharmaceutical functions within the NRPs. Studies of the structure–activity relationships of peptidomimetic inhibitors have indicated that elongating the side chain of Lys through the incorporation of a Har moiety yields more active and more-stable analogues [64]. The metabolic routes to Har formation in bacteria include amidinotransferase AmtA (in *Pseudomonas*) and inosamine-phosphate amidinotransferase McyK (in *Fischerella*) [65]. Notably, Cya-3 was exclusively detected within the PK–NRP albicidin [33]. Its activating domain encompasses an extraordinary insertion of 342 amino acids, probably affiliated with the adenosine nucleotide a-hydrolase superfamily, suggesting that the NRPS subunit AlbIV facilitates the transformation of Asn into Cya-3 *in situ*. Dhb and Dha, generated from Ser and Thr residues, respectively, have profound effects on the pharmaceutical properties [66]. Both often appear together with their biosynthetic precursors, so these NRPS modules must act iteratively.

To enhance their structural complexity, the PCP-domain-bound α-AAMs may undergo further modification by *cis*-acting moieties, such as *N*-methylase, oxidase, or MOX. In this study, we have shown that the modification modalities of *N*-Me, Ox, and MOX typically induce the elongation of A domains. This elongation confers a pronounced affinity for specific moieties. However, some retain congruence with their parental structures (Table 15). Among these structural modification, *N*-Me is a prevalent alteration that contributes significantly to pharmaceutical properties by enhancing proteolytic stability, oral bioavailability, membrane permeability, and selective binding to target sites [67, 68]. The Ox modification transforms protein-bound Ser-S-PCP, Gln-S-PCP, and Cys-S-PCP to oxazole, 5-amino-3H-pyridine-2,6- dione, and thiazole, respectively, on the growing intermediates. These crucial structural motifs often play key roles in the related NRP activity [69]. The MOX modification engages with assembly lines to instate β-hydroxylation, followed by terminal amide formation through the cleavage of the carbon backbone—often contributing significantly to the pharmaceutical efficacy of the final product [70]. In this study, all MOX modules were integrated within Gly-activating domains. However, among the newly identified PK–NRP megapolipeptins biosynthesized, MgpD lacked a MOX module. Conversely, its adjacent MgpG could catalyze the hydroxylation and dealkylation of the Val moiety [58]. β-OH α-AAMs are ubiquitous across NRPS assembly lines [71]. In this study, β-OH showed distinct preferences while maintaining the invariant lengths of its A domains—indicating that this modification requires separately encoded hydroxylases, acting in *trans*. To date, four families of hydroxylases have been discovered within the NRPS biosynthetic pathways. Free hydroxyl groups often act as recognition sites for specific pharmaceutical targets. The Orn moiety is frequently encountered in various modified forms—most notably as unique variants, such as *N*^δ^-OH-, *N*^δ^-OH-*N*^δ^-formyl, *N*^δ^-OH-*N*^δ^-acetyl, *N*^δ^-OH-*N*^δ^-butyryl, *N*^δ^-OH-*N*^δ^-nitroso-, and cyclo-*N*^δ^-OH-Orn. Similarly, Thr, Tyr, and Phe are susceptible to atypical covalent modifications. However, most of these derivatives are indistinguishable from the parent α-AAMs. The inherent complexity associated with α-AAMs is further exacerbated by NRPS–PKS chains. Therefore, the heterologous reconstitution of PKS and NRPS domains in a combinatorial fashion can potentially generate an extensive library of “unnatural” natural products characterized by novel structures.

Importantly, during the complex process of peptide elongation, a total of 41 modules were used in an iterative manner (Table S23)—although some, such as XcnA, may have remained inactive [72]. In stark contrast to this highly coordinated process, their biosynthesis is characterized by iteration and nonlinear NRPSs. However, the fundamental mechanisms underlying these phenomena remain unclear. Nonetheless, notable advances have been made. The amphi-enterobactin “waiting room” model of biosynthesis has been proposed, wherein the thioesterase (TE) domain functions as a “waiting room” for the Ser moiety throughout each iterative cycle [73]. Cereulide is synthesized through TE-mediated trimerization and the macrocyclization of the anticipated tetradepsipeptide substrate based on the canonical activities of NRPS subunits CesA and CesB [74]. DidA operates bimodularly in an iterative fashion to incorporate three to four Gln residues into didemnins, which may involve an export-hydrolysis model [75]. The long-range transactivation whereby the tandem PCP domains of KorC are charged by the A domain of KorD could represent a rate-limiting step [76]. Consequently, unconventional domain organizations present substantial challenges for prediction, and inactive domains further complicate these intricacies.

In summary, over 80% of identified SNSs show exceptional selectivity, so aligning these sequence motifs can allow the analysis of unknown NRPSs while streamlining traditional identification methodologies, although substantial challenges persist due to novel modifications, unconventional domain organizations, and dormant domains, which complicate predictive efforts. However, incorporating these unpredictable elements diversifies and complicates the final product structure. Simultaneously, it inspires researchers to identify additional BGCs with suitable pharmacological activity. An array of bioinformatics tools—including Global Natural Products Social Molecular Networking (GNPS), metabolomics, and various machine learning algorithms-has been introduced into this field, so we believe that this will become an important topic in the foreseeable future.

## Supplemental Materials

Supplementary data for this paper are available on-line only at http://jmb.or.kr.



## Figures and Tables

**Table 1:** SNSs located in Leu-, Ile- and Val-activating domains and their frequency.

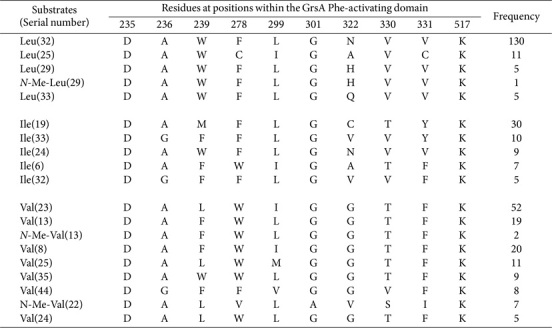

**Table 2:** Identical SNSs located in Leu-/Ile-, Leu-/Val-, Ile-/Val-, and Leu-/Ile-/Val-activating domains.

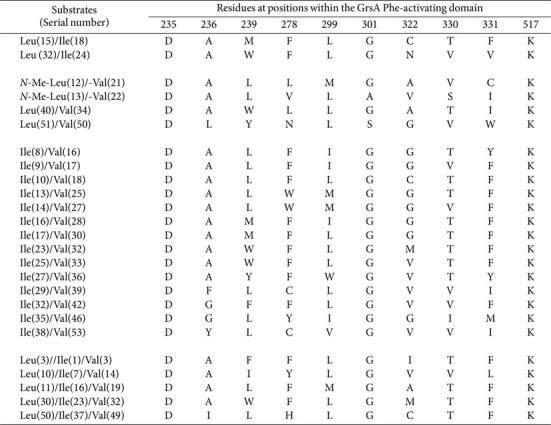

**Table 3:** SNSs located in *N*-Me-Leu-/Val-, *N*-Me-Ile, β-OH-Leu-, Phe-/Leu-, Ala-/Leu-/Val-, Hpg-, and Dhvactivating domains.

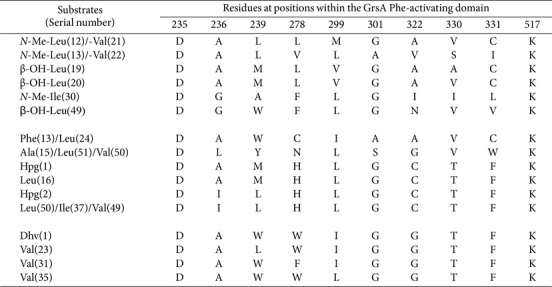

**Table 4:** SNSs located in Val- and Met-activating domains and their similarities.

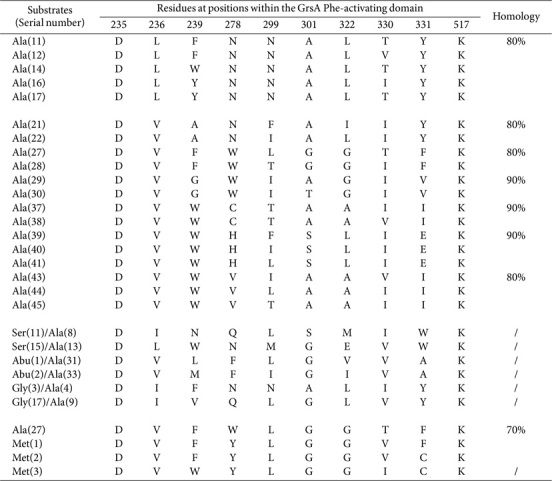

**Table 5:** SNSs located in Gly-activating domains and their similarities.

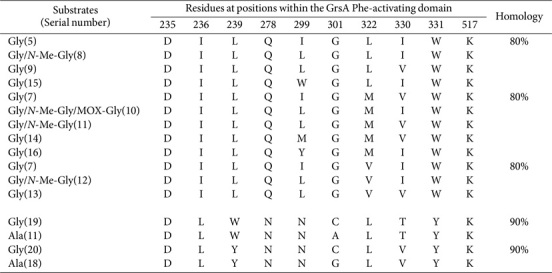

**Table 6:** SNSs located in Orn- and ANPA--activating domains and their similarities.

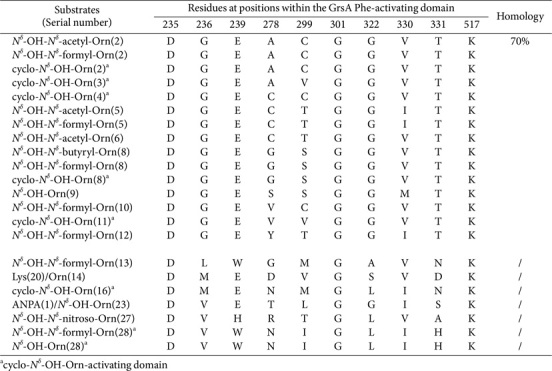

**Table 7:** SNSs located Lys (including Pip)-activating domains and their similarities.

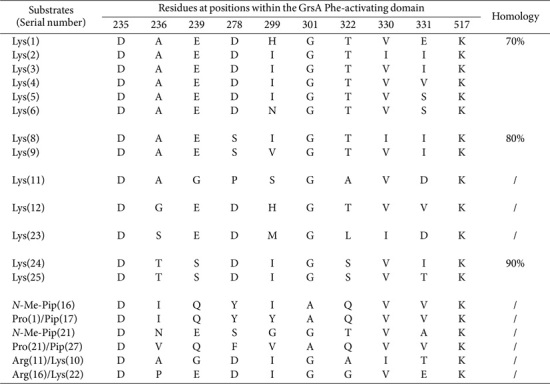

**Table 8:** SNSs located Pro- and AZC-activating domains and their similarities.

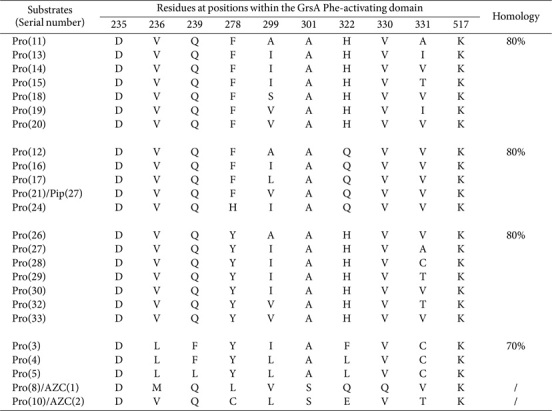

**Table 9:** SNSs located Glu- and Gln-activating domains and their similarities.

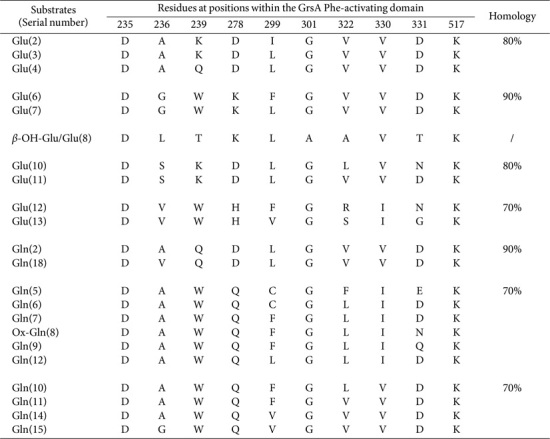

**Table 10:** SNSs located Asp-/AMA-and Asn/Cya-3-activating domains and their similarities.

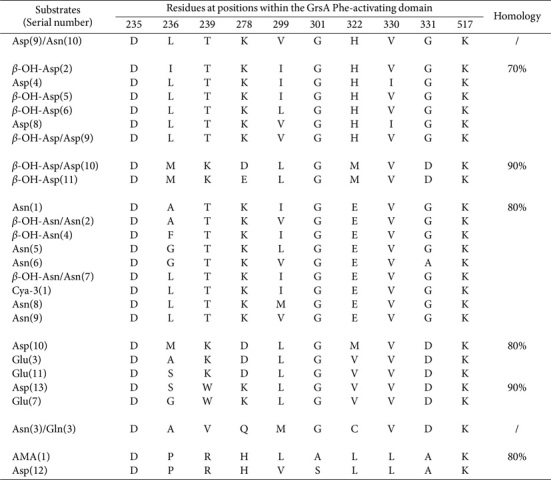

**Table 11:** SNSs located Tyr-activating domains and their similarities.

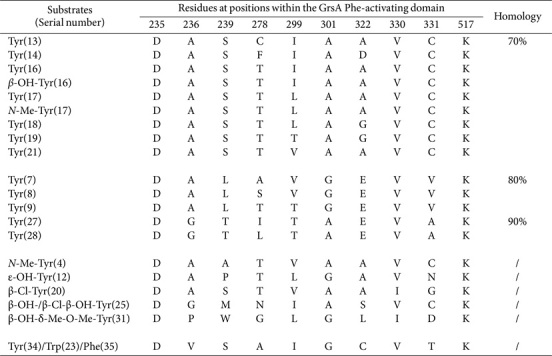

**Table 12:** SNSs located Phe-activating domains and their similarities.

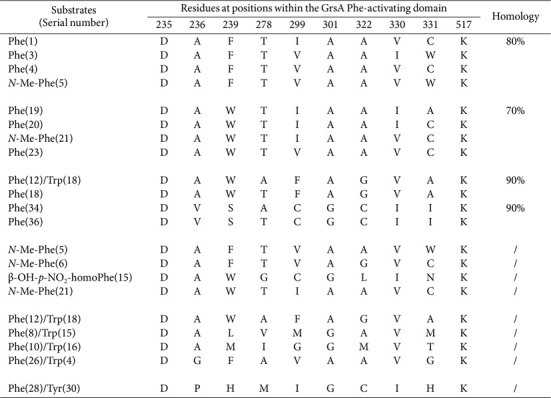

**Table 13:** SNSs located Dab- and Dap-activating domains and their similarities.

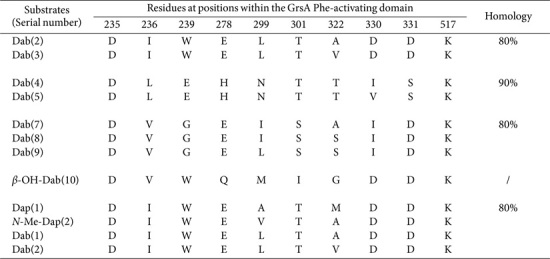

**Table 14:** Specific SNSs located 25 α-AAM-activating domains.

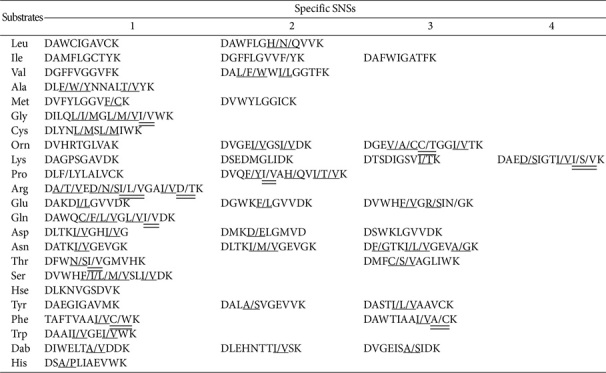

**Table 15:** Usual modification modalities and their preferred moieties.