# Supplementary material for: Comparative Analysis of Signature Sequences from Adenylation Domains Situated within Bacterial-Origin Nonribosomal Peptide Synthetase Modules
Source: J Microbiol Biotechnol. 2025 Jul 14;35:e2502030. doi: 10.4014/jmb.2503.02030 (PMC12283262; doi:10.4014/jmb.2503.02030)
Supplement: Supplementary file 1 [file jmb-35-e2502030-supple.pdf]

J. Microbiol. Biotechnol. 2025. 35: e2502030
https://doi.org/10.4014/jmb.2503.02030

Supplementary Tables

Comparative analysis of signature sequences from adenylation domains situated within bacterial-origin nonribosomal peptide synthetase modules

Weina Gao[1#] Zhishen Zhang[1#] Huiying Yu[2*] Xin Li[2] Chunshan Quan[3] Yun Xue[1]
Pengchao Zhao[1*]

Tables

Table S1 List of NRPSs and hybrid NRPS-PKSs with their recruited substrates, bacterial sources and biological evaluation

Table S2 Occurrence frequency of 36 proteinogenic and non-proteinogenic α-AAMs

Table S3 SNSs located in Leu-activating domains and their NRPS/NRPS-PKS subunits

Table S4 SNSs located in Ile-activating domains and their NRPS/NRPS-PKS subunits

Table S5 SNSs located in Val-activating domains and their NRPS/NRPS-PKS subunits

Table S6 SNSs located in Ala- and Met-activating domains and their NRPS/NRPS-PKS subunits

Table S7 SNSs located in Gly-activating domains and their NRPS/NRPS-PKS subunits

Table S8 SNSs located in Cys-activating domains and their NRPS/NRPS-PKS subunits

Table S9 SNSs located in Orn- and ANPA--activating domains and their NRPS/NRPS-PKS subunits

Table S10 SNSs located Lys (including Pip)-activating domains and their NRPS/NRPS-PKS subunits

Table S11 SNSs located Pro- and AZC-activating domains and their NRPS/NRPS-PKS subunits

Table S12 SNSs located Arg-, End- and Har- activating domains and their NRPS/NRPS-PKS subunits

Table S13 SNSs located Glu-activating domains and their NRPS/NRPS-PKS subunits

Table S14 SNSs located Gln-activating domains and their NRPS/NRPS-PKS subunits

Table S15 SNSs located Asp-/AMA-and Asn/Cya-3-activating domains and their NRPS/NRPS-PKS subunits

Table S16 SNSs located Thr-/Dhb-activating domains and their NRPS/NRPS-PKS subunits

Table S17 SNSs located Ser-/Dha and Hse-/Hsl-activating domains and their NRPS/NRPS-PKS subunits

Table S18 SNSs located Tyr-activating domains and their NRPS/NRPS-PKS subunits

Table S19 SNSs located Phe-activating domains and their NRPS/NRPS-PKS subunits

Table S20 SNSs located Trp-activating domains and their NRPS/NRPS-PKS subunits

Table S21 SNSs located Dab-, Dap- and His-activating domains and their NRPS/NRPS-PKS subunits

Table S22 Bacterial species known to synthesize NRPs through NRPS services, documented from the year 2023 to 2024, alongside their genomic information

Table S23 Subunits in an iterative manner

References

Table S1 List of NRPSs and hybrid NRPS-PKSs with their recruited substrates, bacterial sources and biological evaluation

No.	Product Name	Protein Name [a]	Type	Protein ID (GenBank) [a]	Recruited Substrates [a]	Bacterial sources	Genome ID (GenBank)	Biological Evaluation	References
1	Acinetobactin	BasA	NRP	AB621369 (BAM44995)	Thr	*Acinetobacter haemolyticus* ATCC 17906	None	Siderophore	[1]
2	Aeruginaldehyde/ Enantio-pyochelin	PchE/PFL_3493, **PchF/PFL_3492**	NRP	AAY92760, **AAY92759**	Cys-***N*-Me-Cys**	*Pseudomonas protegens* Pf-5	CP000076	Siderophore	[2]
3	Ajudazols A and B	AjuL, **AjuD**	PK-NRP	AM946600 (CAQ18839, **CAQ18831**)	*N*-Me-Gly-**Ox-Cys**	*Chondromyces crocatus* Cmc5	CP012159	Inhibitor of mitochondrial electron transport	[3]
4	Ala-AMB/Ala-Methoxyvinylglycine	AmbB, **AmbE**	NRP	AAG05693, **AAG05690**	Ala-**Glu**	*Pseudomonas aeruginosa* PAO1	AE004091	Antibacterial	[4]
5	Albicidin	AlbIV	PK-NRP	AJ586576 (CAE52342)	Cya-3	*Xanthomonas albilineans* Xa23R1	None	Phytotoxin, antibacterial	[5]
6–7	Alterochomides A and B	AltK/PPIS_a0417/CKO50_10720, **AltL/PPIS_a0416/CKO50_23365**, AltM/PPIS_a0414/CKO50_23030	NRP	ATD05721/PAY01194, **ATD05720/PAX98997**, ATD05719/PAX99137	Thr-**Val-Asn**-Asn-Leu	*Pseudoalteromonas piscicida* CM 20779/*Pseudoalteromonas* sp. HM-SA03	CP011924, CP011925/ NSDG00000000	Antifungal, antibacterial	[6—7]
8	Althiomycin	AlmA, **AlmB**	PK-NRP	FR831800 (CCA29202, **CCA29203**)	Gly-Ox-Cys-**Ser-Cys-Gly**	*Myxococcus xanthus* DK897	None	Antibacterial	[8]
9	Althiomycin	Alb4/SMDB11_2290, **Alb5/SMDB11_2289**	PK-NRP	CDG12865, **CDG12864**	Gly-Ox-Cys-**Ser-Cys-Gly**	*Serratia marcescens* Db11	HG326223	Antibacterial	[9]
10	Ambactin	AmbS/Xmir_01407	NRP	PHM49485	Ser-Gln-Phe-Leu-Phe-Lys	*Xenorhabdus miraniensis* DSM 17902	NITZ01000000	Anti-protozoic	[10]
11	Amicoumacin	AmiA, **AmiI-1**, AmiJ	PK-NRP	CDH00817, **CDH00813**, CDH00811	Asn-**Asn**-Leu	*Xenorhabdus bovienii* Moldova	CBSV010000000	Anti-inflammator, antiulcer, antibacterial	[11]
12	Amonabactins P 750 and T 789	AmoH/AHA_2473, **AmoF/AHA_2476**, AmoG/AHA_2474	NRP	WP_011706304, **WP_011706307**, WP_011706305	Gly-**Lys-Lys**-[b] Phe/Trp	*Aeromonas hydrophila* ATCC 7966	NC_008570	Siderophore	[12]
13	Amphibactin	AbsE/TW84_00905, **AbsF/TW84_00900**	NRP	KJY94240, **KJY94239**	N^{δ}-OH-N^{δ}-acetyl-Orn-N^{δ}-OH-N^{δ}-acetyl-Orn-Ser-**N^{δ}-OH-N^{δ}-acetyl-Orn**	*Vibrio neptunius* S2394	NZ_JXXU00000000	Siderophore	[13]
14	Amphi-enterobactin	AebF, **AebF**, AebF, **AebF**	NRP	WP_041853220	Ser-**Ser**-Ser-**Ser**	*Vibrio harveyi* BAA-1116	NC_022269, NC_022270/ NC_009783, NC_009784	Siderophore	[14]

[a]: Font with same color in the same row denotes the protein and its ID (GenBank) and recruited substrates;

[b]: denoting these adenylation could accept multiple substrates;

Table S1 Continued

No.	Product Name	ProteinName	Type	Protein ID (GenBank)	Recruited Substrates	Bacterial sources	Genome ID (GenBank)	Biological Evaluation	References
**15**	Andrimid/ Moiramide B	AdmK, **AdmP**	PK-NRP	AY192157 (AAO39105, **AAO39110**)	Val-**Gly**	*Pantoea agglomerans* Eh335	None	Antibacterial, antitumor	[15–16]
**16**	Anguibactin	AngR+AngM	NRP	M34504 (AAA79860, AngR), AY312585 (AAR12528, AAA81775, AngR+AngM)	Cys	*Vibrio anguillarum* pJHC-A103 and 775-pJM1	CP002284 (pJHC-A103), CP002285 (775-pJM1)	Siderophore	[17]
**17**	Anikasin/Cyclic lipopeptide 5	AniA, **AniB**, AniC/ ClpA, **ClpB**, ClpC	NRP	WP_064118561, **WP_064118560**, WP_064118559	Leu-Asp-**Thr-Leu-Leu-Ser**-Leu-Ser-Leu-Ile-Asp	*Pseudomonas fluorescens* HKI0770	LVEJ00000000	Anti-protozoal	[18]
**18**	Antalid	AntB	PK-NRP	KU245058 (ANI24100)	Val-Cys-Phe	*Polyangium spumosum* MSr6761=DSM 14735	None	None	[19]
**19**	Aquimarins	AqmD/L1975_RS21650, **AqmE/ L1975_RS21655**, AqmF/L1975_ RS21660, **AqmG/L1975_RS21665**	PK-NRP	WP_108867381, **WP_108867380**, WP_234859471, **WP_234859472**	Ile-**Ala**-Leu-Ile-Val-Arg-Ala-**Val-Trp-Val-Leu-Val**	*Aquimarina* sp. Aq135	NZ_CAKLCA000000000	Antibacterial	[20]
**20**	Argyrins A–H	Arg2, **Arg3**	NRP	MK047651 (QCE43602, **QCE43603**)	Ala/Abu-Dha-*N*-Me-Gly-**Ala/ Ser-Ox-Cys-Trp-Trp-Gly**	*Cystobacter* sp. SBCb004	None	Antibacterial, antitumor, immunosuppressive	[21]
**21**	Arthrofactin	ArfA, **ArfB**, ArfC	NRP	AB107223 (BAC67534, **BAC67535**, BAC67536)	Leu-Asp-**Thr-Leu-Leu-Ser**-Leu-Ser-Ile-Ile-Asp	*Pseudomonas* sp. MIS38	JAFLXF010000000	Biosurfactant	[22–23]
**22**	Aureusimine	AusA	NRP	BBN44112	Val-Tyr	*Staphylococcus aureus* KUN1163	AP020324	Virulence factors	[24]
**23**	Auriporcine	P615_17235, **P615_17230**, P615_17225	PK-NRP	ERM18329, ERM18328, ERM18327	Pro-Leu-**Pro-Leu**-Leu-Phe	*Brevibacillus laterosporus* PE36	NZ_JXXU00000000	Siderophore	[25]
**24**	Azetidomonamide/ Azabicyclene	AzeB	NRP	AAG06715	Ser-AZC/Pro	*Pseudomonas aeruginosa* PAO1	AE004091	None	[26–27]
**25**	Azotobactin δ	AvCA_25650, **AvCA_25580**, AvCA_25570, **AvCA_25560**	NRP	AGK12767, **AGK12765**, AGK12766, **AGK16636**	Glu-Tyr-Dab-**Asp-Ser-Hse-Gly**-*β*-OH-Asp-Ser-Arg-Hse-N^{δ}-OH-N^{δ}-acetyl-**Orn-Hsl**	*Azotobacter vinelandii* CA	CP005094	Siderophore	[28–29]
**26**	Azotochelin/ Protochelin	AvCA_21190	NRP	AGK13426	Lys	*A. vinelandii* CA	CP005094	Siderophore	[28–29]

Table S1Continued

No.	Product Name	ProteinName	Type	Protein ID (GenBank)	Recruited Substrates	Bacterial sources	Genome ID (GenBank)	Biological Evaluation	References
**27**	Bacillaene	BaeJ, **BaeN**	PK-NRP	AJ634060 (CAG23957, **CAG23960**)/ABS74061, **ABS74064**	Gly-**Ala**	*Bacillus velezensis* FZB42	CP000560	Antibacterial	[30]
**28**	Bacillamide C	Bacillamide synthetase 1/UY9_14934	NRP	EIM09914/JQ687535 (AFJ42298)	Ala-Cys	*Bacillus atrophaeus* C89	AJRJ01000000	Algicidal, antiviral	[31]
**29**	Bacillibactin	DhbF, **DhbF**, DhbF	NRP	JQ073774 (AEW31033)/ AGE64792	Gly-Thr-**Thr-Gly**<br>Thr-Gly	*Bacillus subtilis* XF-1	CP004019	Siderophore	[32]
**30**	Bacillomycin D	BmyD-A, **BmyD-B** BmyD-C	NRP	JQ271536 (AFG19379, **AFG19378**, AFG19377)	Asn-**Tyr-Asn-Pro-Glu**-Ser-Thr	*Bacillus amyloliquefaciens* Q-426	None	Antibacterial, antifungal, antitumor	[33]
**31**	Bacillomycin L	BmyL-A, **BmyL-B**, BmyL-C	NRP	AIW30047, **AIW30046**, AIW30045	Asn-**Tyr-Asn-Ser-Glu**-Ser-Thr	*B. subtilis* Bs-916	CP009611	Antifungal, antitumor	[34]
**32**	Bacillothiazols	NrsC/RBAM_027450	PK-NRP	ABS75103	Cys-Cys-Cys	*B. velezensis* FZB42	CP000560	Antitumor, enzyme inhibitor	[35]
**33**	Bacilosarcin	Bacilosarcin synthetase 1 and **2**	PK-NRP	WP_069840051, **WP_069840050**	Asn-**Leu**	*Bacillus* sp. NBRC 110487=F56	NZ_BDFC00000000	Antiplasmodial, plant growth inhibition	[36]
**34–35**	Bacitracin/ Subpeptin	BatA/SubA, **BatB/SubB**, BatC/SubC	NRP	AF007865 (AAC06346, **AAC06347**, AAC06348)/ DQ272494 (ABB80123, **ABB80124**, ABB80125)	Ile-Cys-Leu-Glu-Ile-**Lys-Orn**-Ile-Phe-His-Asp-Asn	*Bacillus licheniformis* ATCC 10716/*B. subtilis* JM4	None	Antibacterial, antifungal, protease inhibitor	[37–39]
**36**	Bactobolin	BtaK, **BtaN**, BtaE	PK-NRP	ABC34305, **ABC34483**, ABC34137	Ala-**Ala**-Val	*Burkholderia thailandensis* E264	CP000085, CP000086	Antibacterial	[40]
**37**	Bananamides A–C	Ban(BW11P2)A, **Ban(BW11P2)B**, Ban(BW11P2)C	NRP	KX437753 (AOA33121, **AOA33122**, AOA33123)	Leu-Asp-**Thr-Leu-Leu-Gln**-Leu-Ile	*P. fluorescens* BW11P2	LRUN00000000	Antifungal	[41]
**38**	Bananamides D–G	Ban(COW3)A, **Ban(COW3)B**, Ban(COW3)C	NRP	MN480426 (QGQ63518, **QGQ63519**, QGQ63520)	Leu-Asp-**Thr-Leu-Leu-Ser**-Leu-Ile	*Pseudomonas* sp. COW3	JAHTKI000000000	Antifungal	[42]
**39**	Barnesin A	BrnE/Sulba_0748	PK-NRP	AFL68053	Tyr-Arg	*Sulfurospirillum barnesii* SES-3	CP003333	Protease inhibitor	[43]
**40**	Bengamide	BenD	PK-NRP	KP143770 (AJY78094)	*N*-Me-Pip	*Myxococcus virescens* ST200611=DSM 15898	None	Antitumor	[44]
**41**	Bicornutin A1	BicA	NRP	JX424818 (AFP87549)	Arg-Leu-Arg-Arg-Arg	*Xenorhabdus budapestensis* DSM 16342	NZ_NIBS00000000	None	[45]
**42**	Bleomycin-like	BlmVI, **BlmX**, BlmIX, **BlmVII**, BlmIV	PK-NRP	AFV87743, **AFV87758**, AFV87757, **AFV87753**, AFV87750	Ser-Asn-**Asn-His**-Phe-**Gln**-Cys	*Alteromonas mediterranea* AltDE1-pAMDE1	CP003918	Antitumor	[46]
**43**	Bogorol A	BogA, **BogB**, BogC, **BogD**, BogE	NRP	KY810814(ATY37588, **ATY37589**, ATY37590, **ATY37591**, ATY37592)	Dhb-**Leu-Orn**-Ile-Val-Val-Lys-**Val-Leu-Lys-Tyr**-Leu-Val	*B. laterosporus* DSM 25	None	Antibacterial	[47]

Table S1 Continued

No.	Product Name	Protein Subunit Name	Type	Protein ID (GenBank)	Recruited Substrates	Bacterial sources	Genome ID (GenBank)	Biological Evaluation	References
**44**	Bolagladins A and B	BolO, **BolH**	PK-NRP	MT844061 (QNH85847, **QNH85840**)	Asp-**Ser-Val/Ile-Hse-Ser**	*Burkholderia gladioli* HKI052	None	Antibacterial	[48]
**45**	Bovienimides=Lipocitides	XBJ1_2367	NRP	CBJ81493	Leu-Ala-Arg	*X. bovienii* SS-2004	FN667741	None	[49]
**46**	Brabantamides A–C	BraB/BN844_0706	NRP	CDF96614	Ser-Pro	*Pseudomonas* sp. SH-C52	CBLV000000000	Antibacterial, phospholipase inhibitor	[50]
**47**	Brasamide	BraA, **BraB**	NRP	QBZ89968, **QBZ89969**	Val-Ala-Glu/Gln-**Ala-Val-Ala-Pro-Thr**	*Pseudomonas* sp. 11K1	CP035088	Antifungal	[51]
**48**	Brasmycin	BrmB, **BrmC**, BrmA	NRP	QBZ90211, **QBZ90212**, QBZ90205	Ser-Orn-Asp-Hse-His-**Thr-Thr-*β*-OH-Asp**-*γ*-Cl-Thr	*Pseudomonas* sp. 11K1	CP035088	Antifungal	[51]
**49**	Braspeptin	BrpA, **BrpB**, BrpC	NRP	QBZ90202, **QBZ92828**, QBZ90201	Dhb-Pro-Ala-Ile-Ala-Val-Ile-Dhb-Hse-**Val-Ile-Ser-Ala**-Ala-Dab-Val-Thr-Thr-Ala-Dab-Ser-Val	*Pseudomonas* sp. 11K1	CP035088	Antifungal	[51]
**50**	Brevibacillin	BrvA, **BrvB**, BrvC, **BrvD**, BrvE	NRP	MF526970 (ASV51722, **ASV51721**, ASV51723, **ASV51724**, ASV51725)	Dhb-**Leu-Orn**-Ile-Ile-Val-Lys-**Val-Val-Lys-Tyr**-Leu-Val	*B. laterosporus* OSY-I1	NOLX00000000	Antibacterial	[52]
**51**	Brevicidine	BreC, **BreD**	NRP	KY810815 (ATY37608, **ATY37609**)	Asn-Tyr-Trp-Orn-Orn-Gly-Orn-Trp-Thr-**Ile-Gly-Ser**	*B. laterosporus* DSM 25	None	Antibacterial	[53]
**52**	BT1583	BtA, **BtB**, BtC, **BtD**, BtE, **BtF**	NRP	AY953371 (AAY29578, **AAY29579**, AAY29580, **AAY29581**, AAY29582, **AAY29583**)	Dhb-**Leu-Orn**-Ile-Val-Val-Lys-**Val-Leu**-Lys-Tyr-**Leu-Val**	*Brevibacillus texasporus* E58=ATCC PTA-5854	None	Antibacterial	[54]
**53**	Burkholdacs/ Thailandepsins	BhcD/TdpDE1/ BTH_I2364	PK-NRP	ABC36785	Met-Cys-Val/Ile	*B. thailandensis* E264	CP000085, CP000086	HDAC inhibition, antiproliferative	[55–56]
**54–55**	Burkholdines 1215 and 1119/Occidiofungins A–D	BksC/Bamb_6476/OcfH, **BksE/Bamb_6474/OcfF**, BksF/Bamb_6473/OcfE, **BksG/Bamb_6472/OcfD**	NRP	ABI92020, **ABI92018**, ABI92017, **ABI92016/** EU938698 (ADT64846, **ACN32489**, ACN32487, **ACL81528**)	Ser-**β-OH-Tyr/β-Cl-β-OH-Tyr**-Dab-Gly-**Asn-Ser**-*β*-OH-Asn/Asn	*Burkholderia ambifaria* AMMD/*Burkholderia contaminans* MS14	CP000440,CP000441, CP000442/CP009743, CP009744, CP009745	Antifungal	[57–58]
**56**	Burkhomycin	Bkm1, **Bkm2**, Bkm3	NRP	ABA53167, **ABA51457**, ABA52818	Ser-Glu-Ser-**Arg-Thr-Leu-Dab**-Thr-Thr-Glu-Gly-Val	*Burkholderia pseudomallei* 1710b	CP000124, CP000125	Siderophore	[59]
**57**	Burriogladin	BgdA, **BgdB**	NRP	MH170348 (AWC68474), MH170349 (**AWC68475**)	Dhb-Pro-Gln-**Ala-Hpg-Phe-Pro**	*B. gladioli* Lv-StA=HKI0739	None	Biosurfactant	[60]
**58**	Burriogladiodins A–G	BgddA/BM43_4722	NRP	CP009322 (AJW94963)	Dhb-Pro-Gln-Ala-Val/Leu/Ile-Phe-[c] (Pro)	*B. gladioli* ATCC 10248	CP009322, CP009323	Biofilm formation, swarming motility	[61]
**59**	Burrioplantin A	BptE	NRP	WP_045678759	Dhb-Pro-Ser-Ala-Hpg-Phe-Pro	*Burkholderia glumae* LMG 2196=ATCC 33617	CP009434, CP009435	None	[62]

[c]: denoting protein subunits have special adenylation domains and they are active in some products, whereas are inactive in others from the same family;

Table S1 Continued

No.	Product Name	ProteinName	Type	Protein ID (GenBank)	Recruited Substrates	Bacterial sources	Genome ID (GenBank)	Biological Evaluation	References
**60**	Cadasides A and B	CdeI, **CdeJ**, CdeK	NRP	MK060022 (QBC75021, **QBC75022**, QBC75023)	Gly-Glu-Tyr-Thr-Ile-**Asp-*β*-OH-Asp-Pro-Gly-Glu**-*β*-OH-Glu-*N*-Me-Gly	Uncultured bacterium EPI300	None	Antibacterial	[63]
**61**	Cepaciachelin	CphC/Bamb_1686	NRP	ABI87243	Lys	*B. ambifaria* AMMD	CP000440, CP000441, CP000442	Siderophore	[59]
**62**	Cephabacin	CpbK, **CpbI**	PK-NRP	DQ278493 (ABB80393), DQ278492 (**ABB80392**)	Ala-**Ala-Ala-Arg**	*Lysobacter lactamgenus* IFO14288	None	Antibacterial	[64]
**63**	Cereulide	CesA, **CesB**, CesA, **CesB**, CesA, **CesB**	NRP	DQ360825 (ABD14711, **ABD14712**)	Ala-**Val**-Ala-**Val**-Ala-**Val**	*Bacillus cereus* F4810/72	None	Antibacterial	[65]
**64**	Chivosazol	ChiD	PK-NRP	DQ065771 (AAY89051)/ CAN94298	Ser	*Sorangium cellulosum* So ce56	AM746676	Antifungal	[66]
**65**	Chloromyxamide	CmxB	NRP	MH168382 (AXM43052)	Val-Pro/Pip-Pro	*Myxococcus* sp. MCy10608	None	None	[67]
**66**	Chondramide	CmdC, **CmdD**	PK-NRP	AM179409 (CAJ46691, **CAJ46692**)	Ala-***N*-Me-Trp-Tyr**	*C. Crocatus*Cmc5	CP012159	Cytostatic, antifungal	[68]
**67**	Chondrochloren	CndF	PK-NRP	AM988861 (CAQ43084)	*β*-Cl-Tyr	*C. crocatus* Cmc5	CP012159	Antibacterial	[69]
**68**	Cichofactins A and B	CifA, **CifB**	NRP	KJ513093 (AHZ34232, **AHZ34233**)	Leu-Leu-Gln-**Leu-Gln-Val-Leu-Leu**	*Pseudomonas cichorii* SF1-54	None	Swarming, antibacterial, antifungal, phytotoxic	[70]
**69**	Cichopeptins A and B	CipA, **CipB**, CipC, **CipD**, CipE, **CipF**	NRP	KJ513094 (AHZ34238, **AHZ34239**, AHZ34240, **AHZ34241**, AHZ34242, **AHZ34243**)	Dhb-Pro-Ala-Ala-**Ala-Ala-Val**-Dhb-Gly-**Val-Ile-Gly-Ala**-Val-Ala-**Val-Dhb-Thr-Ala-Dab-Ser-Ile/Leu/Val**	*P. cichorii* SF1-54	None	Antibacterial, phytotoxic	[71]
**70**	Colibactin	ClbN, **ClbB**, ClbH, **ClbJ**	PK-NRP	AM229678 (CAJ76286, **CAJ76298**, CAJ76292, **CAJ76290**)	Asn-**Ala**-Ser-**Gly-Cys**	*Escherichia coli* IHE3034	CP001969	Tumor promoting	[72]
**71**	Colistin/Polymyxin E	PE-PmxE, **PE-PmxA**, PE-PmxB	NRP	KP262070 (AJM89738, **AJM89735**, AJM89734)	Dab-Thr-Dab-Dab-Dab-**Leu-Leu-Dab-Dab**-Thr	*Paenibacillus alvei* LR	None	Antibacterial	[73–74]
**72**	Corallopyronin A	CorI	PK-NRP	HM071004 (ADI59531)	Gly	*Corallococcus coralloides* B035	CP034669	Antibacterial	[75]
**73**	Coronatine	CmaA	PK-NRP	AY391839 (AAQ93484)	Ile	*Pseudomonas savastanoi* PG4180	None	Phytotoxic	[76]
**74**	Corpeptin	CrpA/SAMN04490183_1527, **CrpB/SAMN04490183_1528**, CrpC/SAMN04490183_1529	NRP	SDU91457, **SDU91464**, SDU91469/KF192265 [AHY86403(Partial)]	Dhb-Pro-Ala-Ala-Ala-Val-Val-Dhb-Hse-**Val-Ile-Dha-Ala**-Ala-Ala-Val-Dhb-Thr-Ala-Dab-Ser-Ile	*Pseudomonas corrugata* BS3649/ *P. corrugata* CFBP 5454	LT629798 BS3649)	Antibacterial, antifungal	[71,77]
**75**	Crocacin	CroI	PK-NRP	FN547928 (CBD77746)	Gly	*C. crocatus* Cmc5	CP012159	Antibacterial, antifungal, cytotoxic	[78]

Table S1 Continued

No.	Product Name	ProteinName	Type	Protein ID (GenBank)	Recruited Substrates	Bacterial sources	Genome ID (GenBank)	Biological Evaluation	References
**76**	Crocapeptins A and B	CpnD	NRP	AKT40027	Gln-Thr-Leu-Pro-Phe-*N*-Me-Tyr-Val/Ile	*C. crocatus* Cmc5	CP012159	Protease inhibitor	[79]
**77**	Crochelin A	CroD/Achr_38920, **CroF/Achr_38900**, CroG/Achr_38890, **CroH/Achr_38880**	PK-NRP	AJE23278, **AJE23276**, AJE23275, **AJE23274**	Ser-**Ser**-*β*-OH-Asp-Dab-**N^{δ}-OH-N^{δ}-formyl-Orn**	*Azotobacter chroococcum* NCIMB 8003	CP010415	Siderophore	[80]
**78**	Cupriachelin	CucF, **CucG**, CucJ, **CucH**	NRP	CAJ96472, **CAJ96471**, CAJ96468, **CAJ96470**	Asp-Dab-**Asp**-Gly-**N^{δ}-OH-N^{δ}-butyryl-Orn**	*Cupriavidus necator* H16	CP039288, CP039287	Siderophore	[81]
**79**	Cystobactamids 919-1 and 919-2	CysH	NRP	KP836244 (AKP45396)	Asn	*Cystobacter* sp. Cbv34	None	Antibacterial	[82]
**80**	Cystomanamide	CtmA, **CtmC**, CtmD	PK-NRP	KJ710244 (AID65222, **AID65224**, AID65225)	Asn-**β-OH-Asn-β-OH-Phe**-Tyr	*Cystobacter fuscus* MCy9118	None	None	[83]
**81**	Cystothiazole A	CtaC, **CtaD**, CtaG	PK-NRP	AY834753 (AAW03326, **AAW03327**, AAW03330)	Cys-**Ox-Cys**-MOX-Gly	*C. fuscus* AJ-13278	None	Antifungal, antitumor	[84]

Table S1 Continued

No.	Product Name	Protein Subunit Name	Type	Protein ID (GenBank)	Recruited Substrates	Bacterial sources	Genome ID (GenBank)	Biological Evaluation	References
**82**	Delftibactin A	Daci_4756, **Daci_4754**, Daci_4753	NRP	ABX37385, **ABX37383**, ABX37382	Ser-***β*-OH-Asp-Thr-Gly**-Dhb-*N*$^{\delta}$-OH-*N*$^{\delta}$-formyl-Orn-Ser-Arg-cyclo-*N*$^{\delta}$-OH-Orn	*Delftia acidovorans* SPH-1	CP000884	Siderophore, antibacterial	[85]
**83**	Didemnins B, X and Y	DidA, **DidC**, DidD, **DidH**, DidI, **DidJ**	PK-NRP	AFK57212, **AFK57214**, AFK57215, **AFK57219**, AFK57220, **AFK57221**	(Gln-Gln-Gln-Gln)-**Pro**-*N*-Me-Leu-Thr-Ile-**Leu**-Pro-***N*-Me-Tyr**	*Tistrella mobilis* KA081020-065	CP003236–CP003240	Antitumor	[86]
**84**	Disorazole	DszC	PK-NRP	DQ013294 (AAY32966)	Ox-Ser-Ox-Ser	*S. cellulosum* So ce12	None	Antitumor	[87]
**85**	Dkxanthene	DkxA, **DkxG**, DkxJ	PK-NRP	BN001209 (CAQ34914, **CAQ34918**, CAQ34921)	Pro-**Thr**-Asn	*Stigmatella aurantiaca* DW4/3-1	AAMD01000000	Antifungal	[88]
**86**	Ecteinascidin-743/ ET-743	EtuA3, **EtuA2**	NRP	HQ609499 (ADQ55476, **ADQ55475**)	Cys-***β*-OH-*γ*-Me-*δ*-*O*-Me-Tyr-*β*-OH-*γ*-Me-*δ*-*O*-Me-Tyr**	*Candidatus Endoecteinascidia frumentensis*	None	Antitumor	[89]
**87**	Edeine	EdeI, [d] **EdeJ and EdeP**, EdeK	PK-NRP	KC771276 (AHH86043, **AHH86045**)	Gly-[d] **DAHAA**-DAP	*Brevibacillus brevis* Vm4	None	Antibacterial, antitumor	[90]
**88**	Empedopeptin	E7V67_01245, **E7V67_01250**, E7V67_22035	NRP	TXE48326, **TXE48328**, TXE24088	[e] Pro-Ser-Pro-Arg-*β*-OH-Asp-**Ser**-[e] Pro-*β*-OH-Asp	*Empedobacter haloabium* ATCC 31962	VPFC01000000	Antibacterial	[91]
**89**	Endopyrrole A	EpyB, **EpyD**, EpyE	NRPS	MN081797 (QIH29226, **QIH29228**, QIH29229)	Pro-**Val-Pro-Val-Val-Thr**-Ser-Tyr-Tyr	*Burkholderia* (*Paraburkholderia*) *rhizoxinica* HKI 454	FR687359	None	[92]
**90**	Enterobactin	EntF, EntF, EntF	NRP	GU361605 (ADB98044)	Ser-Ser-Ser	*E. coli* O78:K80:H9	None	Siderophore, antitumor, antifungal	[93]
**91**	Entolysin	EtlA/Pseen3332, **EtlB/Pseen3045**, EtlC/Pseen3044	NRP	CAK16086, **CAK15815**, CAK15814	Ile/Leu-Glu-**Gln-Val-Ile/Leu-Gln-Val-Ile/Leu-Gln-Ser**-Val-Ile/Leu-Ser-Ile/Leu	*Pseudomonas entomophila* L48	CT573326	Virulence factors	[94]
**92**	Epothilone	EpoB/EPOS P	PK-NRP	AF217189 (AAF62881)/ AF210843 (AAF26925)	Ox-Cys	*S. cellulosum* SMP44/ *S. cellulosum* So ce90	None	Antitumor	[95]
**93**	Equibactin	EqbE/SEQ_1242	NRP	AM909652 (CAP20364)/ FM204883 (CAW93977)	Cys-Cys-Cys	*Streptococcus equi* 4047	FM204883	Siderophore	[96]
**94**	Fabclavine Ia	FclI, **FclJ**	PK-NRP	PHM26612, **PHM26613**	Asp-Phe-Asn-Asn-**Thr-Val**	*X. budapestensis*DSM 16342	NIBS01000000	Antibacterial, antifungal, anti-protozoa	[97]
**95**	Fabrubactins A and B	FbnO, **FbnP-FbnQ**,FbnH	PK-NRP	WP_010973247, **WP_010973248-WP_010973249**, WP_010973240	Ala-Ox-Cys-**Gly-Ala-Gly**-*ε*-OH-Tyr	*Agrobacterium fabrum* C58	NC_003062, NC_003063	Siderophore	[98]

[d]: denoting that the biosynthesis of an unusual substrate 2,6-diamino-7-hydroxyazelaic acid (DAHAA) might be involved in protein subunit EdeJ and EdeP;

[e]: denoting adenylation domain/protein subunit sequences have not ever been found;

Table S1 Continued

No.	Product Name	ProteinName	Type	Protein ID (GenBank)	Recruited Substrates	Bacterial sources	Genome ID (GenBank)	Biological Evaluation	References
**96–97**	Fengycin B/ Plipastatin	FenA/PpsA, **FenB/PpsB**, FenC/PpsC, **FenD/PpsD**, FenE/PpsE	NRP	JQ271536 (AFG19385, **AFG19384**, AFG19383, **AFG19382**, AFG19381)/ CAB13717, **CAB13716**, CAB13715, **CAB13714**, CAB13713	Glu-Orn-**Tyr-Thr**-Glu-Val-**Pro-Gln-Tyr**-Ile	*B. amyloliquefaciens* Q-426/ *B. subtilis* 168	AL009126 (168)	Antifungal	[33, 99]
**98**	Fimsbactins A−F	FbsF	NRPS	ACIAD2772 (CAG69516)	Ser/Thr	*Acinetobacter baylyi* ADP1	CR543861	Siderophore	[100]
**99**	FK228/FR901228	DepA, **DepD**, DepE, **DepD**	PK-NRP	EF210776 (ABP57745, **ABP57748**, ABP57749, **ABP57748**)	Cys-**Val-Cys**-Dhb-**Val**	*Chromobacterium violaceum* 968	None	Antitumor	[101]
**100**	FR900359	FrsA/BCRE_20005, **FrsD/BCRE_20007**, FrsE/BCRE_20004, **FrsF/BCRE_20003**, FrsG/BCRE_20002	NRP	KNE75168, **KNE75171**, KNE75167, **KNE75166**, KNE75165	*β*-OH-Leu-***β*-OH-Leu**-*N*-Me-Dha-**Ala-*N*-Me-Ala**-*β*-OH-Leu-*N*,*O*-diMe-Thr	*Candidatus Burkholderia crenata* UZHbot9	LGTG00000000	Gq protein inhibitor	[102]
**101–102**	FR901464/ Spliceostatin	Fr9D/Fr9DEF	PK-NRP	HM047288(ADH01485)/ KJ461964 (AIC32693)	Thr	*Pseudomonas* sp. 2663/ *Burkholderia* sp. FERM BP-3421	None	Antitumor	[103–104]
**103**	Fragin	HamD/I35_4194	PK-NRP	HG938371 (CDN62030)	Val	*Burkholderia cenocepacia* H111	HG938370−HG938372	Antifungal	[105]
**104**	Frederiksenibactin	FreF, **FreF**, FreF	NRPS	WP_050504533	Lys-Ser-**Ser-Lys**<br>Ser-Lys	*Yersinia frederiksenii* ATCC 33641	NZ_AALE00000000	Siderophore	[106]
**105**	Fulvuthiacene	FtaB, **FtaE**	PK-NRP	MH069655 (AXM42949, **AXM42952**)	Ox-Cys-**MOX-Gly**	*Myxococcus fulvus* SBMx132	None	None	[107]
**106**	Fusaricidin/LI-F type	FusA	NRP	ABX38811	Thr-Val-Tyr-Thr-Asn-Ala	*Paenibacillus polymyxa* E681	CP000154	Antibacterial, Antifungal	[108]

Table S1 Continued

No.	Product Name	ProteinName	Type	Protein ID (GenBank)	Recruited Substrates	Bacterial sources	Genome ID (GenBank)	Biological Evaluation	References
**107**	Gacamide A	GamA/Pfl01_2211, **GamB/Pfl01_2212**, GamC/Pfl01_2213	NRP	ABA73954, **ABA73955**, ABA73956	Leu-Asp-Gln-**Ile-Leu-Gln-Ser**-Leu-Leu-Ser-Ile	*P. fluorescens* Pf0-1	CP000094	Antibacterial	[109]
**108**	Gladiochelins	GcnH/H9D26_RS17495	NRP	WP_126241403	Ser-Val/Ile-Hse-Ser	*B. gladioli* BCC0238	NZ_CADEVO000000000	Siderophore	[110]
**109**	Glidobactin A	GlbF, **GlbC**	PK-NRP	AKJ29077, **AKJ29080**	Thr-**γ-OH-Lys-Ala**	*Schlegelella brevitalea* K481-B101=ATCC 53080=DSM 7029	CP011371	Antifungal	[111]
**110**	Glidomides	GlmG/AAW51_2776, **GlmH/AAW51_2774**, GlmI/AAW51_2773	NRP	AKJ29466, **AKJ29465**, AKJ29464	β-OH-His-Dab-**β-OH-Asp-Thr**-Ser-Arg-Dab	*S. brevitalea* K481-B101= ATCC 53080=DSM 7029	CP011371	Antitumor, anti-inflammatory	[112]
**111**	Glidopeptin A	GlpC, **GlpD**, GlpE	NRP	AKJ29410, **AKJ29411**, AKJ29412	Ser-Glu-Dab-**Lys-Dhb-Leu-Dab**-Ser-Dhb-Asn-Gly-Dhv	*S. brevitalea* K481-B101= ATCC 53080=DSM 7029	CP011371	Antitumor	[113]
**112**	Gramibactin	GrbI, **GrbJ**	NRP	EDT08697, **EDT08696**	β-OH-Asp-Thr-Thr-N^{δ}-OH-N^{δ}-nitroso-Orn-Gly-**N^{δ}-OH-N^{δ}-nitroso-Orn**	*Paraburkholderia graminis* C4D1M	ABLD00000000	Siderophore	[114]
**113**	Gramibactin B	CUJ87_10420, **CUJ87_10415**	NRP	AXF16146, **AXF14775**	β-OH-Asp-Thr-Thr-N^{δ}-OH-N^{δ}-nitroso-Orn-Gly-**N^{δ}-OH-N^{δ}-nitroso-Orn**	*Paraburkholderia caledonica* PHRS4	CP024905, CP024906	Siderophore	[115]
**114**	Gramicidin S	GrsA,**GrsB**, GrsA, **GrsB**	NRP	D00519 (BAA00406), D29676 (**BAA06146**)	Phe-**Pro-Val-Orn-Leu**-Phe-**Pro-Val-Orn-Leu**	*B. brevis* Nagano	None	Antibacterial, Antifungal	[116]

Table S1 Continued

No.	Product Name	ProteinName	Type	Protein ID (GenBank)	Recruited Substrates	Bacterial sources	Genome ID (GenBank)	Biological Evaluation	References
**115**	Haereogladins A−E	HgdA	NRP	MH170356 (AWC68482)	Dhb-Dhb-*β*-OH-Tyr/Tyr-Hpg	*B. gladioli* Lv-StA=HKI0739	None	Biosurfactant	[60]
**116**	Haereogladiodins A and B	HgddC/BM43_5325	NRP	CP009322 (AJW96749)	Dhb-Dhb-Tyr-(Leu)	*B. gladioli* ATCC 10248	CP009322, CP009323	Biofilm formation, swarming motility	[61]
**117**	Haereomegapolitanin B	HrmC/FNZ07_11705	NRP	CP041743 (QDQ81765)	Dhb-Ser-*β*-OH-Leu-Thr	*Paraburkholderia megapolitana* LMG 23650=DSM23488	CP041743, CP041745	Biosurfactant	[117]
**118**	Haereoplantin A	HptC	NRP	AJK45701	Dhb-Dhb-*β*-OH-Leu-Hpg	*Burkholderia plantarii* PG1=DSM9505	CP002580, CP002581	None	[62]
**119**	Haliamide	HlaA	PK-NRP	ALX87677	Ala	*Haliangium ochraceum* SMP-2=DSM 14365	CP001804	Antitumor	[118]
**120**	Heptarhizin/ Rhizomide A	HepA/RzmA	NRP	CBW76463	Leu-Thr-Tyr-Ala-Ala-Ala-Val	*B. rhizoxinica* HKI 454	FR687359−FR687361	None	[119]
**121**	Heterobactins A, S1 and S2	HtbG	NRP	BAH33409	Arg-Gly-cyclo-N^{δ}-OH-Orn	*Rhodococcus erythropolis* PR4	AP008957	Siderophore	[120]
**122**	Histicorrugatin	HcsF, **HcsG**, HcsH, **HcsI**	NRP	OAB49614, **OAB49613**, OAB49612, **OAB49611**	*β*-OH-His-**Dab-Orn-Ser-Ser**-*β*-OH-Asp-Dab-***β*-OH-His**	*Pseudomonas thivervalensis* LMG 21626	LRSO01000000	Siderophore	[121]
**123**	HM-SA03	SidI, **SidK**, SidM, **SidP**	PK-NRP	PAY02504, **PAY02506**, PAY02508, **PAY02511**	Cys-**Thr**-Ox-Cys-**Thr**	*Pseudoalteromonas* sp. HM-SA03	NSDG00000000	Siderophore	[7]
**124**	Holomycin	Hmo7/Yruck0001_11740	NRP	EEP98516	Cys	*Yersinia ruckeri* ATCC 29473	ACCC01000000	Antibacterial	[122]
**125**	Holrhizin A	HolA/RBRH_01792	NRP	CBW76913	Val-Phe-Glu-Ile-Ala-Ile	*B. rhizoxinica* HKI 454	FR687359−FR687361	None	[119]
**126**	HSAF (heat-stable antifungal factor)/ Lysobacteramide B	HSAF/GLE_3215	PK-NRP	EF028635 (ABL86391)/ ALN58561	Orn	*Lysobacter enzymogenes* C3	CP013140	Antibacterial, antifungal	[123]
**127**	Hypeptin	HynA, **HynB**	NRP	MW759775 (UTQ93744, **UTQ93745**)/CP072597 (QWP75305, **QWP75304**)	Ala-Leu-Arg-*β*-OH-Asn-*β*-OH-Asn-*β*-OH-Tyr-***β*-OH-Leu-Ile**	*Lysobacter* sp. K5869	CP072597	Antibacterial	[124]

Table S1 Continued

No.	Product Name	ProteinName	Type	Protein ID (GenBank)	Recruited Substrates	Bacterial sources	Genome ID (GenBank)	Biological Evaluation	References
**128**	Icosalide	IcoS	NRP	MH790860 (AYA44686)	Leu-Ser-Ser-Leu	*B. gladioli* Lv-StA=HKI0739	None	Antibacterial	[125]
**129**	Indigoidine	IgiD	NRP	AF088856 (AAD54007)	Ox-Gln-Ox-Gln	*Vogesella indigofera* ATCC 19706	None	Pigment	[126]
**130**	Iturin A	ItuA-A, **ItuA-B**, ItuA-C	NRP	AB050629 (BAB69698, **BAB69699**, BAB69700)	Asn-**Tyr-Asn-Gln-Pro**-Asn-Ser	*B. subtilis* RB14	None	Antifungal	[127]
**131**	Iturin A-like/ Bacillorin	BacA, **BacB**, BacC	NRP	FJ194462 (ACI22671, **ACI22672**, ACI22673)/ AIW30047, **AIW30046**, AIW30045	Asn-**Tyr-Asn-Ser-Glu**-Ser-Thr	*B. subtilis* Bs-916	CP009611	Antifungal	[128]
**132**	Jagaricin	JagA, **JagB**, JagC, **JagD**	NRP	HE967328 (CCJ67645, **CCJ67646**, CCJ67646, **CCJ67646**)/CDG80824, **CDG80825**,CDG80826, **CDG80827**	Dhb-Thr-**Thr-Tyr**-Dhb-Gln-Gly-**Thr-His**	*Janthinobacterium agaricidamnosum* NBRC 102515=DSM 9628	HG322949	Antifungal	[129]
**133**	Jessenipeptin	JesA, **JesB**, JesC	NRP	MT505440 (QMS47799, **QMS47798**, QMS47800)	Thr-Pro-Ile-Val-Ala-**Ala-Ala-Val-Ser-Ala**-Val-Ala-Leu-Thr-Thr-Ala-Dab-Ser-Phe	*Pseudomonas* sp. QS1027	PHSU01000000	Antibacterial	[130]
**134**	Kalimantacin A/ Batumin	Bat2	PK-NRP	GU479979 (ADD82940)	Gly	*P. fluorescens* BCCM_ID9359	None	Antibacterial	[131]
**135**	Kleboxymycin	NpsB-Kleboxymycin	PK-NRP	MF401554 (ATJ04411)	Pro	*Klebsiella oxytoca* MH43-1	None	Cytotoxicity	[132]
**136**	Kolossin A	PluTT01m_13700/ KolA/Plu2670	NRP	CP024901 (AXG47721)/ BX571868 (CAE15044)	Leu-Ala-Leu-Val-Tyr-Leu-Val-Thr-Val-Leu-Val-Thr-Leu-Val-Val	*Photorhabdus luminescens* TT01	CP024901/BX571859−BX571875	None	[133]
**137**	Koranimine	KorA, **KorB**, KorD, **KorC**, KorD	NRP	JF828091 (AEC14346, **AEC14347**, AEC14349, **AEC14348**, AEC14349)	Thr-Leu-Leu-**Phe**-Val-**Phe**-Val	*Bacillus* sp. NK2003	None	None	[134]
**138**	Kurstakins	KrsA/bthur0010_59520, **KrsB/bthur0010_59530**, KrsC/bthur0010_59540	NRP	EEM74022, **EEM74023**, EEM74024	Thr-**Gly-Ala**-Ser-His-Gln-Gln	*Bacillus thuringiensis* BGSC 4BA1	CM000755/ ACNH00000000	Antifungal	[135]

Table S1 Continued

No.	Product Name	ProteinName	Type	Protein ID (GenBank)	Recruited Substrates	Bacterial sources	Genome ID (GenBank)	Biological Evaluation	References
**139**	Labrenzin	FJ695_03635	PK-NRP	QDG75033	Gly	*Labrenzia* sp. PHM005	CP041191	None	[136]
**140**	Lagriamide	LgaA, **LgaF**	PK-NRP	MH171092 (AXA20090, **AXA20091**)	Gly-**Gly**	*B. gladioli* Lv-StA=HKI0739	None	Antifungal	[137]
**141**	Laterocidine	LatC, **LatD**	NRP	AIG26884,**AIG26883**	Ser-Tyr-Trp-Orn-Orn-Gly-Orn-Trp-Thr-**Ile-Asn-Gly-Gly**	*B. laterosporus*LMG 15441	CP007806	Antibacterial	[138]
**142**	Le-pyrrolopyrazines A−C	LedE, **LedF**	NRP	MF495862 (ATD51279, **ATD51280**)	Val/Ile/Leu-**Pro**	*L. enzymogenes* OH11	RCTY01000000	None	[139]
**143**	Leupyrrin	Leu5, **LeuA**, LeuE	PK-NRP	HM639990 (ADZ24989, **ADZ24995**, ADZ24999)	Pro-**Thr/*β*-OH-Thr**-Pro	*S. cellulosum* So ce690	None	Antifungal	[140]
**144**	Lichenysin D	LicA, **LicB**, LicC	NRP	U95370 (AAD04757, **AAD04758**, AAD04759)	Gln-Leu-Leu-**Val-Asp-Leu**-Ile	*B. licheniformis* ATCC 10716	None	Biosurfactant, Antibacterial, Antifungal	[141]
**145**	Linear gramicidins A−D	LgrA, **LgrB**, LgrC, **LgrD**	NRP	AJ566197 (CAD92849, **CAD92850**, CAD92851, **CAD92852**)	Val/Ile-Gly-**Ala-Leu-Ala-Val**-Val-Val-Trp-Leu-Trp/Phe/Tyr-Leu-**Trp-Leu-Trp-Gly**	*B. brevis* ATCC 8185	None	Antifungal	[142]
**146**	Locillomycin	LocA, **LocB**, LocC	NRP	KF866134 (AHI59108, **AHI59109**, AHI59110)	Thr-**Gln-Asp-Gly-Asn-Asp-Gly**-Tyr-Val	*B. subtilis* Bs-916	CP009611	Antibacterial, antiviral	[143]
**147**	Lokisin	LokC, **LokB,** LokA	NRP	MK534107 (QDF82254, **QDF82255**, QDF82259)	Leu-Asp-**Thr-Leu-Leu-Ser**-Leu-Ser-Leu-Ile-Asp	*Pseudomonas koreensis* COR10	None	Antifungal	[144]
**148**	Lugdunin	LugD/SLUG_08150, **LugA/SLUG_08100**, LugB/SLUG_08120, **LugC/SLUG_08130**	NRP	CCB53264, **CCB53266**, CCB53267,**CCB53269**	Cys-**Val-Trp**-Leu-**Val-Val-Val**	*Staphylococcus lugdunensis* N920143	FR870271	Antibacterial	[145]
**149**	Luminmides/ GameXPeptide	Plu3263/GxpS	NRP	CP024901 (AXG48275)/ BX571870 (CAE15637)	Val-Leu-Phe/Leu-Leu-Leu	*P. luminescens* TT01	CP024901/BX571859−BX571875	Anti-protozoa	[146–147]
**150**	Luminmycins A−C	Plu1878, **Plu1880**	PK-NRP	AXG47007/CAE14171, **AXG49819/CAE14173**	Thr-**Lys-Ala**	*P. luminescens* TT01	CP024901/BX571859−BX571875	Antitumor	[146]
**151**	Lysobactin	LybA, **LybB**	NRP	JF412274 (AEH59099, **AEH59100**)	Leu-Leu-*β*-OH-Phe-*β*-OH-Leu-**Leu-Arg-Ile-Thr-Gly-*β*-OH-Asn-Ser**	*Lysobacter* sp. ATCC53042	None	Antibacterial	[148]
**152**	Lysocins A−I	LesA, **LesB**	NRP	LC128664 (BAV56270, **BAV56271**)	Thr-Arg-Ser-Gly-*N*-Me-Phe-Leu-Arg-**Glu-Gln-Trp-Val/Ile-Thr**	*Lysobacter* sp. RH2180-5	None	Antibacterial	[149–150]

Table S1 Continued

No.	Product Name	Protein Subunit Name	Type	Protein ID (GenBank)	Recruited Substrates	Bacterial sources	Genome ID (GenBank)	Biological Evaluation	References
**153**	Macyranone	MynB/CYFUS_005573, **MynD/CYFUS_005575**, MynF/CYFUS_005577	PK-NRP	ATB40125,**ATB40127**, ATB40129	Thr-**Thr-Leu**-His	*C. fuscus*MCy9118=DSM 52655	CP022098 (DSM 52655)	Anti-protozoa	[151]
**154**	Malleobactins A−C	MbaI, **MbaJ**	NRP	CAH35777, **CAH35776**	N^{δ}-OH-Orn/ANPA-*β*-OH-Asp-Ser-**N^{δ}-OH-N^{δ}-formyl-Orn**	*B. pseudomallei*K96243	BX571965, BX571966	ECF sigma factor, Siderophore	[152]
**155**	Marthiapeptide A	MarA/ASG16_028345, **MarB/ASG16_028340**	NRP	RAT94091, **RAT94090**	Phe-Ile-**Ala-Cys-Cys-Cys-Cys**	*Brevibacillus* sp. Leaf182	LMPN02000000	Antibacterial, antitumor	[153]
**156**	Massetolide	MassA, **MassB**, MassC	NRP	EU199080 (ABH06367), EU199081 (**ABH06368**, ABH06369)	Leu-Glu-**Thr-Ile-Leu-Ser**-Leu-Ser-Ile	*P. fluorescens* SS101	None	Antimycobacterial	[154]
**157**	Massiliachelin	ACZ75_02210	PK-NRP	AKU20507	*N*-Me-Cys-*N*-Me-Cys	*Massilia* sp. NR 4-1	CP012201	Siderophore	[155]
**158**	Megapolibactins	MegH/MFNZ07_17560, **MegI/FNZ07_17565**, MegJ/FNZ07_17570	NRP	QDQ83031,**QDQ83032**, QDQ83033	Gly-**β-OH-Asp-Ser-Thr-N^{δ}-OH-N^{δ}-nitroso-Orn-Ala/ Ser**-N^{δ}-OH-N^{δ}-nitroso-Orn	*P. megapolitana* LMG 23650=DSM23488	CP041743, CP041745	Siderophore	[115]
**159**	Melithiazol	MelC, **MelD**, MelG	PK-NRP	AJ557546 (CAD89774, **CAD89775**, CAD89778)	Cys-**Ox-Cys**-MOX-Gly	*Melittangium lichenicola*Me l46	None	Antifungal	[156]
**160**	Micacocidin	MicC/RSc1806, **MicH/RSc1811**	PK-NRP	CAD15508, **CAD15513**	*N*-Me-Cys-*N*-Me-Cys-***N*-Me-Cys**	*Ralstonia solanacearum* GMI1000/OE1-1	AL646052, AL646053	Antimycoplasma, siderophore	[157]
**161**	Microsclerodermin M	MscF, **MscH**, MscI	PK-NRP	KF657738 (AHB82056, **AHB82058**, AHB82059)	Asn-***N*-Me-Gly-Trp-Gly**-Gly	*S. cellulosum* So ce38	None	Antifungal	[158]
**162**	Mutanobactins	SMU_1342, **SMU_1341c**, SMU_1340, **SMU_1339**	PK-NRP	AAN59015,**AAN59014**, AAN59013, **AAN59012**	Leu-Ala-**Pro**-Val/Ile-Cys-**Gly**	*Streptococcus mutans*UA159	AE014133	Antifungal	[159]
**163**	Mycosubtilin	MycA, **MycB**, MycC	NRP	AF184956 (AAF08795, **AAF08796**, AAF08797)	Asn-**Tyr-Asn-Gln-Pro**-Ser-Asn	*B. subtilis* ATCC 6633	CP034943/ ADGS01000000	Antifungal	[160−161]
**164**	Myxalamid	MxaA	PK-NRP	AF319998 (AAK57184)	Ala	*S. aurantiaca* Sga15	None	Antibacterial, antifungal	[162]
**165**	Myxochelin	MxcG	PK-NRP	AF299336 (AAG31130)	Lys	*S. aurantiaca* Sga15	None	Siderophore	[163−164]
**166**	Myxochromide A	MchA-B, **MchA-C**	NRP	KX622592 (APZ78743, **APZ78744**)	*N*-Me-Thr-Ala-**Leu-Pro-Ala-Gln**	*M. fulvus* HW-1/*M. xanthus*	None	Biosurfactant	[165]
**167**	Myxochromide B	MchB-B, **MchB-C**	NRP	KX622591 (APZ78728, **APZ78729**)	*N*-Me-Thr-Ala-**Leu-Leu-Pro-Ala-Gln**	Myxococcus sp. 171	None	Biosurfactant	[165]
**168**	Myxochromide C	MchC-B, **MchC-C**	NRP	KX622594 (AQM37583, **AQM37584**)	*N*-Me-Thr-Ala-**Leu-Pro-Gln**	*M.virescens* ST200611=DSM 15898	None	Biosurfactant	[165]
**169−170**	Myxochromide D	MchD1-B, **MchD1-C**/ MchD2-B, **MchD2-C**	NRP	KX622588 (APZ78691, **APZ78692**)/KX622587 (APZ78679, **APZ78680**)	*N*-Me-Thr-Ala-**Leu-Ala-Gln**	*Hyalangium minutum*DSM 14724/ *Cystobacterineaebacterium* CcG34	JMCB00000000 (DSM 14724)	Biosurfactant	[165]

Table S1 Continued

No.	Product Name	ProteinName	Type	Protein ID (GenBank)	Recruited Substrates	Bacterial sources	Genome ID (GenBank)	Biological Evaluation	References
**171**	Myxochromide S	MchS-B, **MchS-C**	NRP	KX622600 (APZ78833, **APZ78834**)	*N*-Me-Thr-Leu-**Ala/Abu-Ala-Gln**	*S. aurantiaca* Sga15	None	Biosurfactant	[165]
**172**	Myxoprincomide-c506	MXAN_3779	PK-NRP	ABF87031	*N*-Me-Ser-Leu-*β*-OH-Val-Ser-Val-Ser-Tyr-Ala	*M. xanthus* DK 1622	CP000113	Predation	[166]
**173**	Myxopyronin	MxnI	PK-NRP	KF356280 (AGS77289)	Gly	*M. fulvus* Mx f50	None	Antibacterial	[167]
**174**	Myxothiazol	MtaC, **MtaD**, MtaG	PK-NRP	AF188287 (AAF19811, **AAF19812**, AAF19815)	Cys-**Ox-Cys**-MOX-Gly	*S. aurantiaca* DW4/3-1	AAMD00000000	Antifungal	[168]
**175**	Myxovirescin A	Ta1	PK-NRP	AJ006977 (CAB38084)	Gly	*M. xanthus* ER-15	None	Antibacterial	[169]
**176**	Nannocystin A	NcyE, **NcyF**	PK-NRP	KT067736 (ALD82525, **ALD82526**)	*N*-Me-Ile-**Tyr-*β*-OH-Val**	*Nannocystis* sp. MB1016	None	Antitumor	[170]
**177**	Nevaltophin	Pb62B	NRP	KR871222 (ANG60378)	*N*-Me-Val	*Xenorhabdus* sp. PB62.4	NKHR00000000	Anti-protozoa	[171]
**178**	Ngercheumicins	EA58_10630	NRP	KDM91475	Leu-Thr-Ser-Thr-Leu-Leu	*Photobacterium galatheae* S2753	JMIB00000000	None	[172]
**179**	*N*-octanoyl-Met-Phe-H	BN712_00198	NRP	FR902169 (CDE97356)	Met-Phe	*Clostridium* sp. CAG:567	CBJZ000000000	Enzyme Inhibitor	[173]
**180**	Nunamycin	NunD, **NunE**, NunB1	NRP	KC880158 (AHL29289, **AHL29290**, AHL29299)/ LIRD01000002 (KPN93065, **KPN90369**, KPN90374)	Ser-Dab-Gly-Hse-Dab-**Thr-Thr-*β*-OH-Asp**-*γ*-Cl-Thr	*P. fluorescens* In5	LIRD01000000	Antifungal, synergistic antitumor	[174]
**181**	Nunapeptin	NupA, **NupB**, NupC	NRP	KC880158 (AHL29288, **AHL29287**, AHL29286)/ LIRD01000000 (KPN93063, **KPN93064**, KPN90376)	Dhb-Pro-Ala-Ala-Ala-Val-Ala-Dhb-Ser-**Val-Ile-Dha-Ala**-Val-Ala-Val-Dhb-Thr-Ala-Dab-Ser-Ile	*P. fluorescens* In5	LIRD01000000	Antifungal, synergistic antitumor	[174]
**182**	Obafluorin	ObiF	NRP	KX134687 (ARJ35751)/ KX931446 (AQZ26587)	*β*-OH-*p*-NO_2-homoPhe	*P. fluorescens* ATCC 39502	None	Antibacterial	[175]
**183**	Octapeptin C4	OctA, **OctB**, OctC	NRP	LN999013 (CUX79060, **CUX79061**, CUX79062)	Dab-Dab-Dab-**Phe-Leu-Dab-Dab**-Leu	*Bacillus circulans* ATCC 31805	None	Antibacterial	[176]
**184**	Odilorhabdins	Odl1, **Odl2**, Odl3, **Odl4**	NRP	LT966404 (SOV25619, **SOV25620**, SOV25621, **SOV25622**)	Lys-*β*-OH-Dab-*β*-OH-Dab-**Gly-Orn-Pro**-His-**β-OH-Lys/Lys-α, β-dehydro-Arg-β-OH-Lys/Lys**	*Xenorhabdus nematophila* K102	None	Antibacterial	[177]
**185**	Onnamide	FJ695_03635, **FJ695_03625**	PK-NRP	QDG75035, **QDG75033**	Gly-**Arg**	*Labrenzia* sp. PHM005	CP041191	None	[136]
**186**	Orfamide	OfaA, **OfaB**, OfaC	NRP	KT613918 (ALG76234, **ALG76235**, ALG76236)/ CP027705 (AZC17620, **AZC17621**, AZC17622)	Leu-Glu-**Thr-Ile/Val-Leu-Ser**-Leu-Leu-Ser-Val	*Pseudomonas* sp. CMR5c	CP027705	Antifungal	[178−179]
**187**	Ornibactin	OrbI, **OrbJ**	NRP	KY523076 (AUD11994, **AUD11993**)/KAG8153120, **KAG8153007**	N^{δ}-OH-Orn-*β*-OH-Asp-Ser-**N^{δ}-OH-N^{δ}-formyl-Orn**	*Burkholderia catarinensis* 89	MDEQ02000000	Siderophore	[180]

Table S1 Continued

No.	Product Name	ProteinName	Type	Protein ID (GenBank)	Recruited Substrates	Bacterial sources	Genome ID (GenBank)	Biological Evaluation	References
**188**	Pacifibactin	PfbG/S7S_05305, **PfbI/S7S_05315**, PfbJ/S7S_05320, **PfbK/S7S_05325**	PK-NRP	AJD47481, **AJD47483**, AJD47484, **AJD47485**	Ser-***β*-OH-Asp**-Arg-*β*-OH-Asp-Ser-**N^{δ}-OH-N^{δ}-acety-Orn-cyclo-N^{δ}-OH-Orn**	*Alcanivorax pacificus* W11-5	CP004387	Siderophore	[181]
**189**	Paenibacterin	PbtA, **PbtB**, PbtC	NRP	JX899679 (AGM16412, **AGM16413**, AGM16414)	Orn-Val-Thr-Orn-Ser-**Val-Lys-Ser-Ile-Pro**-Val-Lys-Ile	*Paenibacillus thiaminolyticus* OSY-SE	NZ_ALKF00000000	Antibacterial	[182]
**190**	Paenibactin/ Paebacillibactin	PaeF, **PaeF**, PaeF	NRP	HQ668144 (AEI70245)	Ala-Thr-**Thr-Ala** (branch: Thr-Ala)	*Paenibacillus elgii* B69	NZ_AFHW00000000	Siderophore	[183]
**191**	Paenilamicins A1, B1, A2 and B2	PamA, **PamB**, PamC, **PamD**, PamE, **PamH**	PK-NRP	AHD05614, **AHD05615**, AHD05616, **AHD05617**, AHD05618, **AHD05621**	Arg/Lys-Ala-*N*-Me-Dap-**Orn/Lys**-Ser-***N*-Me-Dap-Gly**	*Paenibacillus larvae* DSM25430	CP003355	Antifungal, antibacterial	[184]
**192**	Paenilarvins A−C	ERIC2_c18770, **ERIC2_c18760**, ERIC2_c18750	NRP	AHD05679, **AHD05678**, AHD05677	Asn-**Tyr-Asn-Gln-Pro**-Asn-Asn	*P. larvae* DSM 25430	CP003355	Antifungal	[185]
**193**	Paenilipoheptin	PhnC, **PhnD**, PhnE	NRP	AUW27332, **ADM70190**, AUW27333	Ser-**Dab**-Phe-Val-Phe-Tyr-Glu	*P. polymyxa* E681	CP000154	None	[108]
**194**	Patellazole	PtzF	PK-NRP	AFX98752	Cys	*Candidatus Endolissoclinum faulkneri* L2	CP003539	Antitumor	[186]
**195**	PAX-peptides	PaxA/XNC1_2783, **PaxB/XNC1_2782**, PaxC/XNC1_2781	NRP	CBJ90837, **CBJ90836,** CBJ90835	Gly-**Lys-Lys-Lys**-Lys-Lys-Lys	*X. nematophila*ATCC 19061	FN667742	Antibacterial, antifungal	[187]
**196**	Pederin	PedF, **PedH**	PK-NRP	AX768043 (CAE01106, **CAE01108**)	Gly-**Arg**	*P. aeruginosa*-like (Bacterial symbiont of *Paederus fuscipe)*	None	Antitumor	[188−189]
**197**	Pelgipeptins	PlpD, **PlpE**, PlpF	NRP	JQ745271 (AFJ14793, **AFJ14794**, AFJ14795)	Dab-**Val/Ile-Dab-Phe-Leu-Dab-Val-Leu**-Ser	*P. elgii* B69	NZ_AFHW00000000	Antifungal, antibacterial	[190]
**198**	Pellasorens	PelA	PK-NRP	HE616533 (CCE88377)	Ala	*S. cellulosum* So ce38	None	Antitumor	[191]
**199**	Pentacitidins	SAMN04488128_101 1792	NRP	SJZ83675	His-Ile-Val-Pro-Phe	*Chitinophaga eiseniae* DSM 22224	NZ_FUWZ00000000	Protease inhibitor	[192]
**200**	Phenalamides/ Stipiamides	MYSTI_04320	PK-NRP	AGC45618	Ala	*Myxococcus stipitatus* DSM 14675	CP004025	Antiviral, antitumor	[193]
**201**	Photoditritide	PdtS/MEG1_RS04325	NRP	KER04351	Har-Har-Tyr-Trp-Tyr-Trp	*Photorhabdus temperata* Meg1	JGVH00000000	Antiprotozoal, antibacterial	[194]
**202**	Photohexapeptide	PhpS	NRP	MN099046 (QDJ95742)	Ile-Ile-Ile-Ile-Leu-Ile	*Photorhabdus asymbiotica* PB68.1	None	None	[195]
**203**	Phototemtide	PttB/MEG1_RS04970, **PttC/EG1_RS04975**	NRP	KER04254, **KER04255**	Gly-Val-Phe-**Thr-Ile**	*P. temperata* Meg1	JGVH00000000	Antiprotozoal	[196]
**204**	Photoxenobactins	PxbF/Xsze_03167, **PxbG/Xsze_03168**	PK-NRP	PHM32421, **PHM32422**	Cys-Cys-**Ox-Cys-Cys**	*Xenorhabdus szentirmaii* DSM 16338	NIBV00000000	None	[197]
**205**	Phymabactin	PhmA, **PhmB**	NRP	ACC73162, **ACC73161**	Asp-Asp-Ser-**Cys**	*Paraburkholderia phymatum* STM815	CP001043, CP001044	Siderophore	[198]

Table S1 Continued

No.	Product Name	ProteinName	Type	Protein ID (GenBank)	Recruited Substrates	Bacterial sources	Genome ID (GenBank)	Biological Evaluation	References
**206**	Piscibactin	Irp2, **Irp1**	PK-NRP	KP100338 (AKQ52531, **AKQ52532**)	*N*-Me-Cys-Cys-**Cys**	*Photobacterium damselae* DI21	KB405046–KB405055	Siderophore	[199]
**207**	Plantaribactin	PblM/BGL_2c16910, **PblL/BGL_2c16900**, PblD/BGL_2c16990, **PlbE/BGL_2c16980**	NRP	AJK49758, **AJK49757**, AJK49766, **AJK49765**	*β*-OH-Asp-Ser-**Ser-Orn**-N^{δ}-OH-N^{δ}-nitroso-Orn-Gln-Gly-**N^{δ}-OH-N^{δ}-nitroso-Orn**	*B. plantarii* PG1=DSM9505	CP002580, CP002581	Siderophore	[115]
**208**	Poaeamide	PoaA, **PoaB**, PoaC	NRP	AGE26255, **AGE25479**, AGE25480	Leu-Glu-**Thr-Leu-Leu-Ser**-Leu-Leu-Ser-Ile	*Pseudomonas poae* RE1-1-14	CP004045	Antifungal	[200]
**209**	Polymyxin A	PA-PmxE, **PA-PmxA**, PA-PmxB	NRPS	ADM71964, **ADM71968**, ADM71967	Dab-Thr-Dab-Dab-Dab-**Leu-Thr-Dab-Dab**-Thr	*P. polymyxa* E681	CP000154	Antibacterial	[201]
**210**	Polymyxin B	PB-PmxE, **PB-PmxA**, PB-PmxB	NRPS	JN660148 (AEZ51520, **AEZ51516**, AEZ51517)	Dab-Thr-Dab-Dab-Dab-**Phe-Leu-Dab-Dab**-Thr	*P. polymyxa* PKB1	None	Antibacterial	[202]
**211**	Polymyxin D	PD-PmxE/PPYC1_03510, **PD-PmxA/PPYC1_03490**, PD-PmxB/PPYC1_03500	NRPS	APB69496, **APB69492**, APB69494	Dab-Thr-Ser-Dab-Dab-**Leu-Thr-Dab-Dab**-Thr	*P. polymyxa* YC0136	CP017967	Antibacterial	[203]
**212**	Polymyxin P	PP-PmxE, **PP-PmxA**, PP-PmxB	NRPS	FR727736 (CBY05535, **CBY05531**, CBY05532)/ CCI71204, **CCI71208**, CCI71207	Dab-Thr-Dab-Dab-Dab-**Phe-Thr-Dab-Dab**-Thr	*P. polymyxa* M-1	HE577054	Antibacterial	[204]
**213**	Prezeamine	Zmn16, **Zmn17**	PK-NRP	HE995400 (CCM44336, **CCM44337**)	AMA-Phe-Asn-Asn-**Thr-Val**	*Serratia plymuthica* RVH1	ARWD00000000	Antibacterial	[205]
**214**	Pristinamycin	PxnM, **PxnG**, PxnH	PK-NRP	CBJ89817, **CBJ89823**, CBJ89822	Gly-**Ser**-Pro	*X. Nematophila* ATCC 19061	FN667742	Antibacterial	[206]
**215**	Prodigiosin	HapI	NRP	DQ266254 (ABB69081)/ CP000155 (ABC32682)	Pro	*Hahella chejuensis* KCTC 2396	CP000155	Antifungal,antibacterial, antiprotozoal, antitumor, immunosuppressant	[207]
**216**	Promysalin	PpgJ	NRP	GU211010 (ADQ74618)/ CP077094 (QXI32361)	Pro	*Pseudomonas promysalinigenes* RW10S1	CP077094	Antibacterial	[208]
**217**	Prosekin	PekA/SAMN05216222_4719, **PekB/SAMN05216222_4720**, PekC/SAMN05216222_4721	NRPS	SDT49871, **SDT49885**, SDT49904	Leu-Glu-**Thr-Leu-Leu-Ser**-Ile-Ile	*Pseudomonas proseki* LMG 26867^{T}	LT629762	None	[209]
**218**	Protegomycin 945	XPG1_1613, **XPG1_1612**	NRPS	CDG21268, **CDG21267**	Phe-Trp-Tyr-**Tyr-Trp**	*Xenorhabdus poinarii* G6	FO704551	None	[187]
**219**	Protegomycin 1085	XDD1_1908, **XDD1_1908-XDD1_1907**, XDD1_1906	NRPS	CDG17607, **CDG17607-CDG17606**, CDG17605	Phe-**Tyr-Tyr**-Tyr-Tyr-Trp	*Xenorhabdus doucetiae* FRM16	FO704550	None	[187]
**220**	Pseudoalterobactins	PabB, **PabF**, PabI, **PabG**, PabI, **PabJ**	PK-NRP	PAY00812, **PAY00808**, PAY00805, **PAY00807**, PAY00805, **PAY00804**	Lys-**Asn**-*β*-OH-Asp-**Lys-Arg/Lys**-*β*-OH-Asp-**Gly**	*Pseudoalteromonas* sp.HM-SA03	NSDG00000000	Siderophore	[7]
**221**	Pseudodesmin	PdmA, **PdmB**, PdmC	NRPS	MT577358 (QLY89262, **QLY89263**, QLY89264)	Leu-Gln-**Thr-Val-Leu-Ser**-Leu-Ser-Ile	*Pseudomonas* sp. COR52	None	Antifungal	[210]
**222**	Pseudomonine	PmsG	NRP	EF484930 (ABS50184)	Thr	*P. fluorescens* WCS374	CP007638	Siderophore	[211]

Table S1 Continued

No.	Product Name	ProteinName	Type	Protein ID (GenBank)	Recruited Substrates	Bacterial sources	Genome ID (GenBank)	Biological Evaluation	References
223	Pseudovibriamides A and B	PppA/KGB56_24770, **PppB/KGB56_24765**, PppC/KGB56_24760, **PppD/KGB56_24755**	PK-NRP	CP074128 (QUS58939, **QUS58938**, QUS58937, **QUS58936**)	Tyr-Dhb-Gly/Ala-**Arg-Pro-Cys**-Gln-(**Pro-Val/Ile/Leu**)	*Pseudovibrio brasiliensis* Ab134	CP074126	Promoting motility, reducing biofilm formation	[212]
224	Putisolvins I and II	PsoA, **PsoB**, PsoC	NRP	DQ151887 (ABW17375, **ABW17376**, ABW17377)	Leu-Glu-**Leu-Ile-Gln-Ser-Val-Ile-Ser**-Leu-Val/Ile-Ser	*Pseudomonas putida* PCL1445	None	Inhibiting biofilm formation, biosurfactant	[213]
225	Pyochelin	PchE-PA14, **PchF-PA14**	NRP	AF184621 (AAD55800), AF184622 (**AAD55801**)	Cys-**N-Me-Cys**	*P. aeruginosa*PA14	ASWV01000000	Siderophore	[214]
226	Pyoluteorin	PltF	PK-NRP	AAD24881	Pro	*P. protegens* Pf-5	CP000076	Antifungal	[215]
227–228	Pyoverdine DC3000= Pyoverdine 1448A (Type III)	PSPPH_1911/PSPTO_2135, **PSPPH_1923/PSPTO_2147**, PSPPH_1924/PSPTO_2148, **PSPPH_1925/PSPTO_2149**, PSPPH_1926/PSPTO_2150	NRP	AAZ34524/AAO55652, **AAZ34950/AAO55664**, AAZ34484/AAO55665, **AAZ36245/AAO55666**, AAZ36468/AAO55667	Glu-Tyr-Dab-**Lys**-β-OH-Asp-Thr-**Thr-Ser**-β-OH-Asp-Ser	*P. savastanoi* 1448A/ *Pseudomonas syringae* DC3000	CP000058/ AE016853	Siderophore	[216–217]
229	Pyoverdine 206-12 (Type III)	PvdL(III),**PvdI(III)**, PvdJ(III), **PvdD(III)**	NRP	AY765261 (**AAX16327**, AAX16326, **AAX16325**)	Glu-Tyr-Dab-**Ser-Dab-N^{δ}-OH-N^{δ}-formyl-Orn**-Gln-**Gln-N^{δ}-OH-N^{δ}-formyl-Orn-Gly**	*P. aeruginosa* 206-12	None	Siderophores	[218]
230	Pyoverdine 2-164 (Type II)	PvdL(II),**PvdI(II)**, PvdJ(II)	NRP	AF540993 (**AAO17442**, AAO17441)	Glu-Tyr-Dab-**Ser-N^{δ}-OH-N^{δ}-formyl-Orn-Orn**-Gly-Thr-Ser-N^{δ}-OH-N^{δ}-formyl-Orn	*P. aeruginosa* 2-164	None	Siderophores	[218]
231	Pyoverdine DSM 21245 (Type II)	PvdL/PT01550, **PvdI/PT02609**, PvdJ(1)/PT02607, **PvdJ(2)/PT02606**	NRP	KM036007 (AJW67516), **KM036023 (AJW67532)**, KM036025 (AJW67534), **KM036026 (AJW67535)**	Glu-Tyr-Dab-**Ser-Lys**-β-OH-His-**Thr-Ser-cyclo-N^{δ}-OH-Orn**	*Pseudomonas taiwanensis* DSM 21245	AUEC01000000	Siderophores	[219]
232	Pyoverdine GB-1 (CFML 90-51, Type II)	PputGB1_3809, **PputGB1_4086**, PputGB1_4085, **PputGB1_4084**, PputGB1_4083	NRP	ABY99699, **ABY99976**, ABY99975, **ABY99974**, ABY99973	Glu-Tyr-Dab-**Asp-Lys**-β-OH-Asp-Ser-**Gly**-Thr-Lys-cyclo-N^{δ}-OH-Orn	*P. putida* GB-1	CP000926	Siderophores	[220]
233	Pyoverdine KT2440 (Type II)	PP_4243, **PP_4221**, PP_4220, **PP_4219**	NRP	AAN69823, **AAN69802**, AAN69801, **AAN69800**	Glu-Tyr-Dab-**Asp-Orn**-β-OH-Asp-Dab-**Gly-Ser-cyclo-N^{δ}-OH-Orn**	*P. putida* KT2440	AE015451	Siderophores	[221]
234	Pyoverdine PAO1 (Type I)	PvdL/PA2424, **PvdI/PA2402**, PvdJ/PA2400, **PvdD/PA2399**	NRP	AAG05812, **AAG05790**, AAG05788, **AAG05787**	Glu-Tyr-Dab-**Ser-Arg-Ser-N^{δ}-OH-N^{δ}-formyl-Orn**-Lys-N^{δ}-OH-N^{δ}-formyl-Orn-**Thr-Thr**	*P. aeruginosa* PAO1	AE004091	Siderophores	[218]
235	Pyoverdine Pf0-1 (Type III)	PvdL//Pfl01_3940, **PvdI/Pfl01_1845**, PvdJ/Pfl01_1846, **PvdK/Pfl01_1847**, Pfl01_1849	NRP	ABA75677, **ABA73588**, ABA73589, **ABA73590**, ABA73592	Glu-Tyr-Dab-**Ala-N^{δ}-OH-N^{δ}-acetyl-Orn-Orn-Ser**-Ser-Ser-Arg-**β-OH-Asp**-Thr	*P. fluorescens* Pf0-1	CP000094	Siderophores	[222]
236	Pyoverdine Pf17400 (Type II)	PvdL/BG51_RS20610, **PvdI/BG51_RS09210**, PvdJ(1)/BG51_RS09205, **PvdJ(2)/BG51_RS09200,** PvdD/BG51_RS09160, **PvdK(1)/BG51_RS09165**, PvdK(2)/BG51_RS09170	NRP	WP_029298138, **WP_050492772**, WP_020289233, **WP_003179641**, WP_050492767, **WP_050492768**, WP_029293146	Glu-Tyr-Dab-**Ala-Lys-Gly**-Gly-**β-OH-Asp**-Gln-Dab-**Ser**-Ala-cyclo-N^{δ}-OH-Orn	*P. fluorescens* ATCC 17400	NZ_JENC0-1000000	Siderophores	[223]

Table S1 Continued

No.	Product Name	ProteinName	Type	Protein ID (GenBank)	Recruited Substrates	Bacterial sources	Genome ID (GenBank)	Biological Evaluation	References
**237**	Pyoverdine Pf-5 (Type I)	PvdL/PFL_4189, **PvdI/PFL_4095**, PvdJ/PFL_4094, **PvdD/PFL_4093**	NRP	AAY93445, **AAY93356**, AAY93355, **AAY93354**	Glu-Tyr-Dab-**Asp-*N*$^{\delta}$-OH-*N*$^{\delta}$-formyl-Orn-Lys**-Thr-Ala-**Ala-*N*$^{\delta}$-OH-*N*$^{\delta}$-formy-Orn-Lys**	*P. protegens* Pf-5	CP000076	Siderophores	[224]
**238**	Pyoverdine PY$_{Othi}$ (Type II)	PvdL/APS14_09630, **APS14_17630**, APS14_17625, **APS14_17620**	NRP	OAB50036, **OAB54756**, OAB54755, **OAB54754**	Glu-Tyr-Dab-**Ala-*N*$^{\delta}$-OH-*N*$^{\delta}$-Ac-Orn-Gly**-Thr-Thr-Gln-**Gly-Ser-cyclo-*N*$^{\delta}$-OH-Orn**	*P. thivervalensis* LMG 21626	LRSO01000000	Siderophores	[225]
**239**	Pyoverdine SBW25 (Type I)	Pflu6137, **PFLU2543/PvdI**, PFLU2544/PvdJ	NRP	WP_015885148, **WP_012723748**, WP_012723747	Glu-Tyr-Dab-**Ser-Lys-Gly-*N*$^{\delta}$-OH-*N*$^{\delta}$-formyl-Orn**-Lys-*N*$^{\delta}$-OH-*N*$^{\delta}$-formyl-Orn-Ser	*P. aeruginosa* SBW25	NC_012660	Siderophores	[226]
**240**	Pyoverdine SMX-1	**DCC84_23185**, DCC84_23180, **DCC84_23175**	NRP	**QBQ12465**, QBQ12464, **QBQ12463**	Glu-Tyr-Dab-**Ser-*N*$^{\delta}$-OH-*N*$^{\delta}$-Ac-Orn-Gly**-Thr-Thr-Gln-**Gly-Ser-cyclo-*N*$^{\delta}$-OH-Orn**	*Pseudomonas* sp. SXM-1	CP038001	Siderophore	[227]
**241**	Pyreudione	Pys	NRP	WP_064118616	Pro	*P. fluorescens* HKI0770	LVEJ00000000	Amoebicidal, antibacterial	[228]
**242**	Pyrrolizixenamide	PxaA/ALJ96775	PK-NRP	KR827050 (ALJ96775)/ PHM65440	Ser-Pro	*Xenorhabdus stockiae* DSM 17904	NJAJ01000000	None	[229]
**243**	Pyrronazol B	PynC, **PynH**	PK-NRP	MF817819 (ATG32071, **ATG32076**)	Pro-**Ox-Ser**	*Nannocystis pusilla* Ari7	None	None	[230]
**244**	Pyxipyrrolones A and B	PyxE	PK-NRP	KY765914 (ASA76632)	Ser	*Pyxidicoccus* sp. MCy9557	None	Antitumor	[231]
**245**	Ralsolamycin/ Ralstonins	RmyA/Rsp0641, **RmyB/Rsp0642**	PK-NRP	CAD17792, **CAD17793**	Thr-*β*-OH-Tyr-Ser-Hse-Gly-Hse-**Val/Ile-Dha-Gly-Ala**	*R. solanacearum* GMI1000/OE1-1	AL646052, AL646053	Chlamydospore-inducing	[232]
**246**	Reutericyclin	RtcN	PK-NRP	KJ659887 (AJO68335)	Leu	*Lactobacillus reuteri* TMW1.656	JOSW00000000	Antibacterial	[233−234]
**247**	Rhabdopeptides 1−6	RdpA/XNC1_2228, **RdpB/XNC1_2229**, RdpC/XNC1_2230	NRP	CBJ90287, **CBJ90288**, CBJ90289	*N*-Me-Leu/*N*-Me-Val-***N*-Me-Leu/ *N*-Me-Val-Val**-*N*-Me-Val-(*N*-Me-Val)-(*N*-Me-Val)	*X. Nematophila* ATCC 19061	FN667742	Anti-trypanosomal, antiplasmodial	[235]
**248**	Rhizopodin	RizB, **RizD**	PK-NRP	FR854394 (CCA89326, **CCA89328**)	Gly-**Ser**	*S. aurantiaca* Sg a15	None	Antitumor	[236]
**249**	Rhizoxin	RhiB/DFH15_1390	PK-NRP	AM411073 (CAL69889)	Ser	*B.rhizoxinica* B1	None	Antimitotic	[237]
**250**	Rhizoxins D, D$_1$, D$_3$, S$_1$, S$_2$, Z$_1$ and WF1360F	RzxB/PFL_2989	PK-NRP	AAY92261	Ser	*P. protegens* Pf-5	CP000076	Antimitotic	[238]
**251**	Rhodochelin	RHA1_ro02319	NRP	ABG94125	Thr--*N*$^{\delta}$-OH-*N*$^{\delta}$-formyl-Orn<br>*N*$^{\delta}$-OH-*N*$^{\delta}$-formyl-Orn	*Rhodococcus jostii* RHA1	CP000431	Siderophores	[239]
**252**	Ririwpeptides	Plu3123/PluTT01m_16070	NRP	AXG48137/CAE15497	Arg-Ile-Arg-Ile-Trp	*P. luminescens* TTO1	CP024901/ BX571859−BX571875	None	[240]

Table S1 Continued

No.	Product Name	ProteinName	Type	Protein ID (GenBank)	Recruited Substrates	Bacterial sources	Genome ID (GenBank)	Biological Evaluation	References
**253**	Safracin	SacA, **SacB,** SacC	NRP	AY061859 (AAL33756, **AAL33757**, AAL33758)	Ala-**Gly**-*β*-OH-*δ*-Me-*O*-Me-Tyr-*β*-OH-*δ*-Me-*O*-Me-Tyr	*P. fluorescen* A2-2	None	Antibacterial, antitumor	[241]
**254**	Saframycin Mx1	SafB, **SafA**	NRP	MXU24657 (AAC44128, **AAC44129**)	Ala-**Gly-*β*-OH-*δ*-Me-*O*-Me-Tyr-*β*-OH-*δ*-Me-*O*-Me-Tyr**	*M. xanthus* DM504-15	None	Antibacterial, antitumor	[242]
**255**	Seongsanamides A–D	BSL056_RS13665, **BSL056_RS13660**	NRP	APJ11946, **APJ11945**	Leu-Ala-Tyr-Thr-Leu-**Leu-Ile-Tyr**	*Bacillus safensis* KCTC 12796BP	CP018197	Antiallergic	[243]
**256**	Serobactin	Hsero_2343	NRP	ADJ63842	*β*-OH-Asp-Ser-*β*-OH-Asp-Thr-Ser-cyclo-N^{δ}-OH-Orn	*Herbaspirillum seropedicae* SmR1	CP002039	Siderophores	[244]
**257**	Serrawettin W2	SwrA/ATE40_RS18480	NRP	AOF02338	Leu-Ser-Thr-Phe-Ile	*Serratia surfactantfaciens* YD25	CP016948	Biosurfactant, antibacterial	[245]
**258**	Sessilin A	SesA, **SesB**, SesC	NRP	JQ309920 (AFH75320, **AFH75321**, AFH75322)/QXH39399/AZC26604, **QXH39398/AZC26605**, QXH39397/AZC26606	Dhb-Pro-Ser-Leu-Val-Gln-**Leu-Val-Val-Gln-Leu-Val**-Dhb-Thr-Ile-Hse-Dab-Lys	*Pseudomonas sessilinigenes* CMR12a	CP077074/CP027706	Biofilm formation, insect toxicity	[246]
**259**	Sevadicin	SevA, **SevB**	NRP	KF318360 (AGZ03650, **AGZ03651**)	Phe-**Ala-Trp**	*P. larvae* DSM 25430	CP003355	Antibacterial	[247]
**260**	Spiruchostatins A–B	SpiA, **SpiDE1**	PK-NRP	JQ045344 (AFR69331, **AFR69334**)	Cys-**Ala-Cys-Val/Ile**	*Pseudomonas* sp. Q71576	None	Histone deacetylase inhibitors	[248]
**261**	Stechlisins	SteA, **SteB**, SteC	NRP	MT080808 (QNL34616, **QNL34617**, QNL34618)	Leu-Asp-**Thr-Leu-Leu-Ser**-Leu-Gln-Leu-Ile-Glu	*Pseudomonas* sp. FhG100052	None	Antibacterial	[249]
**262**	Sulfazecin	SulI, SulM	NRP	KX757706 (AOZ21316, **AOZ21320**)	Glu-**Ala-Dap**	*Pseudomonas acidophila* ATCC 31363	MTZV00000000	Antibacterial	[250]
**263**	Surfactin	SrfA-A, **SrfA-B**, SrfA-C	NRP	AIW28683, **AIW28684**, AIW28685	Glu-Leu-Leu-**Val-Asp-Leu**-Leu	*B. subtilis* Bs-916	CP009611	Antifungal, antibacterial	[34]
**264**	Syringafactins A–F	SyfA/Pspto_2828, **SyfB/Pspto_2833**	NRPS	AAO56328, **AAO56329**	Leu-Leu-Gln-**Leu-Thr-Val/Ile-Leu-Leu**	*P. syringae* DC3000	AE016853	Bio-emulsifying, biodegradation	[251]
**265**	Syringolin A	SylC, **SylD**	PK-NRP	AJ548826 (CAD70194, CAD70195)	Val-**Lys-Val**	*P. syringae* B301D-R	JALJ00000000	Antitumor (proteasome inhibitor)	[252]
**266**	Syringomycin E	SyrE, **SyrB1**	NRP	AF047828 (AAC80285), U25130 (AAA85160)/AKF46129, AKF46132	Ser-Ser-Dab-Dab-Arg-Phe-Dhb-*β*-OH-Asp-***γ*-Cl-Thr**	*P. syringae* B301D	CP005969	Antifungal	[253]
**267**	Syringopeptin SP22 Phv	SypA-Psyr_2614, **SypB-Psyr_2615**, SypC-Psyr_2616	NRP	AAY37653, **AAY37654**, AAY37655	Dhb-Pro-Val-Leu-Ala-**Ala-Ala-Val-Dhb-Ala**-Val-Ala-Ala-Dhb-Thr-Ser-Ala-Dhb-Ala-Dab-Dab-Tyr	*P. syringae* B728a	CP000075	Antifungal,, antibacterial	[254]
**268**	Syringopeptin SP22-A+B	SypA-PsyrB_13255, **SypB-PsyrB_13260**, SypC-PsyrB_13265	NRP	AF286216 (AAF99707, **AAO72424**, AAO72425)	Dhb-Pro-Val-Val-Ala-**Ala-Val-Val-Dhb-Ala**-Val-Ala-Ala-Dhb-Thr-Ser-Ala-Dhb-Ala-Dab-Dab-Tyr	*P. syringae* B301D	CP005969	Antifungal, antibacterial	[255]
**269**	Syringopeptin SP25	SypA-PsyrH_12990, **SypB-PsyrH_12985**, SypC-PsyrH_12980	NRP	AKF51378, **AKF51377**, AKF51376	Dhb-Pro-Val-Ala-Ala-Val-Leu-Ala-**Ala-Dhb-Val-Dhb-Ala**-Val-Ala-Ala-Dhb-Thr-Ser-Ala-Val-Ala-Dab-Dab-Tyr	*P. syringae* HS191	CP006256	Antifungal, antibacterial	[256]
**270**	Szentiamide	Xsze_03460	NRP	PHM32713	Leu-Thr-Phe-Val-Tyr-Trp	*X. szentirmaii*DSM 16338	NIBV00000000	Antiplasmodial	[187]

Table S1 Continued

No.	Product Name	ProteinName	Type	Protein ID (GenBank)	Recruited Substrates	Bacterial sources	Genome ID (GenBank)	Biological Evaluation	References
**271**	Taiwachelin	TaiE, **TaiF**, TaiG	NRP	CU633750 (CAQ71827, **CAQ71828**, CAQ71829)	β-OH-Asp-**Thr-Asp**-N^{δ}-OH-N^{δ}-butyryl-Orn-Ser-cyclo-N^{δ}-OH-Orn	*Cupriavidus taiwanensis* LMG19424	CU633749, CU633750	Siderophore	[257]
**272**	Tauramamide	BrL25_22185	NRP	CP017705 (ATO51563)	Tyr-Ser-Leu-Trp-Arg	*B. laterosporus* DSM 25	None	Antifungal, antibacterial	[258]
**273**	Taxlllaids	TxlA/XBJ1_0775, **TxlB/XBJ1_0774**	NRP	CBJ79916, **CBJ79915**	Thr-Ala-Leu-Leu-**Leu-Leu-Ala**	*X. bovienii* SS-2004	FN667741	Anti-protozoa	[259]
**274**	Teixobactin	Txo1, **Txo2**	NRP	KP006601 (AJF34463, **AJF34464**)	*N*-Me-Phe-Ile-Ser-Gln-Ile-Ile-**Ser-Thr-Ala-End-Ile**	*Eleftheria terrae* ISO18629	None	Antibacterial	[260]
**275**	Thailandamides A and B	ThaiH	PK-NRP	ABC35522	Ala	*B. thailandensis* E264	CP000085, CP000086	Antibacterial	[261]
**276**	Thailanstatins A−C	TstDEF	PK-NRP	JX307851 (AGN11881)	Thr	*B. thailandensis* MSMB43	AJXB01000000	Antitumor, pre-mRNA splicing inhibitors	[262]
**277**	Thalassospiramides B-like, C-like and F	TtcA, **TtcB**, TtcC	PK-NRP	KC181864 (AGC65513, **AGC65514**, AGC65515)	Phe/Tyr-**Val-Ser-(Val-Ser)-(Val-Ser)-Val**-Tyr	*Thalassospira* sp. CNJ-328	None	Immunosuppressive	[263]
**278–279**	Thalassospiramides A4 and E	TtmA/TttA, **TtmB/TttB**	PK-NRP	AFK56006, **AFK55404**/ KC181865 (AGC65516, **AGC65517**)	(Val)-Val-Ser-Val-Ser-Val-**Tyr**	*T. mobilis* KA081020-065/ *Tistrella bauzanensis* TIO7329	CP003236−CP003240 (KA081020-065)	Immunosuppressive	[263]
**280**	Thanafactin A	ThfA, **ThfB**	NRP	MT431590 (QKM21619, **QKM21620**)	Val-Ala-Gln-**Ala-Val-Ala-Pro-Thr**	*Pseudomonas* sp. SH-C52	CBLV000000000	Enzyme Inhibitor	[264]
**281**	Thanamycin	ThaA, **ThaB**, ThaC1	NRP	HQ888764 (AED90002, **AED90003**, AED90004)	Ser-Orn-Asp-Lys-His-**Thr-Dhb-β-OH-Asp**-γ-Cl-Thr	*Pseudomonas* sp. SH-C52	CBLV010000000	Antifungal	[265−266]
**282**	Thanapeptin	TnpA, **TnpB**, TnpC	NRP	WP_084213811, **WP_052435185**, WP_041024117	Dhb-Pro-Ala-Ala-Ala-Val-Val-Dhb-Hse-**Val-Ile-Dha-Ala**-Ala-Ala-Val-Dhb-Thr-Ala-Dab-Ser-Ile/Leu	*Pseudomonas* sp. SH-C52	CBLV010000000	Antibacterial, antifungal	[267]
**283**	Thaxteramides A and B	ThxA2	PK-NRP	MK551162 (QDA77059)	Ser-Thr-Tyr-Gly-Tyr	*Jahnella thaxteri* MSr9139	None	Antibacterial	[268]
**284**	Thaxteramide C	ThxC2	PK-NRP	MK551161 (QDA77045)	Asn-Tyr-Gly-Tyr	*J. thaxteri* MSr9139	None	Antibacterial	[268]
**285**	Thiomarinol BGC	HolA	PK-NRP	FN689524 (CBK62746)	Cys-Cys	*Pseudoalteromonas* sp. SANK 73390	None	Antibacterial	[269]
**286–287**	Thuggacin	TugD/TgaC	PK-NRP	GQ981381 (ADH04660)/ GQ981380 (ADH04641)	Ox-Cys	*C. crocatus* Cmc5/ *S. cellulosum* So ce895	CP012159 (Cmc5)	Antibacterial	[270]
**288**	Tilivalline	NpsB-Tilivalline	NRP	HG425356 (CDG76959)	Pro	*K. oxytoca* AHC-6	None	Activation of mucosal immune cells	[271]
**289**	Tolaasins I and F	TaaA, **TaaB**, TaaC, **TaaD**, TaaE	NRP	HE967327 (CCJ67636, **CCJ67637**, CCJ67638, **CCJ67639**, CCJ67640)	Dhb-Pro-Ser-**Leu-Val-Ser**-Leu-Val-Val-**Gln-Leu-Val**-Dhb-Thr-Ile/Leu-Hse-Dab-Lys	*Pseudomonas costantinii* DSM 16734	None	Antibacterial, antifungal	[272]
**290**	Trichrysobactin	CbsF	NRP	WP_221853741	Lys-Ser-Ser-Lys<br>Lys-Ser	*Dickeya chrysanthemi* EC16	NZ_JAFCAF-00000000	Siderophore	[106]

Table S1 Continued

No.	Product Name	ProteinName	Type	Protein ID (GenBank)	Recruited Substrates	Bacterial sources	Genome ID (GenBank)	Biological Evaluation	References
**291**	Tridecaptins A1, A3, A4	Tri-D, **Tri-E**	NRP	KF111342 (AHF21228, **AHF21229**)/ KJD42169, KJD42170	Val-Dab-Gly-Ser-Trp/Phe-Ser-Dab-Dab-Phe-Glu-**Val-Ile-Ala**	*Paenibacillus terrae* NRRL B-30644	JTHP00000000	Antibacterial	[273]
**292**	Tubulysin	TubB, **TubC**, TubD	PK-NRP	GU002154 (ADH04678, **ADH04679**, ADH04680)	*N*-Me-Pip-**Ile-*N*-Me-Val**-Cys-Tyr/Phe	*Cystobacter* sp. SBCb004	None	Antifungal, antitumor	[274]
**293**	Turnerbactin	TnbF/ TERTU_RS18085	NRP	WP_041590315	Orn-Ser-Ser-Orn / Orn-Ser	*Teredinibacter turnerae* T7901	NC_012997	Siderophore	[275]
**294**	Tyrocidines	TycA, **TycB**, TycC	NRP	AF004835 (AAC45928, **AAC45929**, AAC45930)	Phe-**Pro-Phe/Trp-Phe**-Asn-Gln-Tyr-Val-Orn-Leu	*B. brevis* ATCC 8185	None	Antibacterial	[276]
**295**	Ulbactins F and G	BGP74_RS13430, **BGP74_RS13415**	NRP	WP_069848010, **WP_069848004**	*N*-Me-Cys-*N*-Me-Cys-**Cys**	*B. brevis* TP-B0800= NBRC 110488	NZ_BDFB00000000	Antitumor	[277]
**296**	Vacidobactin	Vapar_3743, **Vapar_3746**, Vapar_3744, **Vapar_3742**	PK-NRP	ACS20359, **ACS20362**, ACS20360, **ACS20358**	N^{δ}-OH-N^{δ}-formyl-Orn-**Thr**-N^{δ}-OH-Orn/Orn-Ser-**β-OH-Asp**	*Variovorax paradoxus* S110	CP001635	None	[278]
**297**	Vanchrobactin	VabF	NRP	AM168450 (CAJ45639)	Arg-Ser	*V. anguillarum* RV22	None	Siderophore	[279]
**298**	Variobactin	Var3, **Var7**, Var5, **Var6**	PK-NRP	KT362218 (ALG65336, **ALG65342**, ALG65340, **ALG65341**)	Arg-**N^{δ}-OH-N^{δ}-acetyl-Orn-N^{δ}-OH-N^{δ}-formyl-Orn**-β-OH-Asp-**Ser-Pro**	*V. paradoxus* P4B	None	Siderophore	[278]
**299**	Variochelin	VarF, **VarH**, VarI, **VarJ**	PK-NRP	KT900023 (AMR00556, **AMR00554**, AMR00553, **AMR00552**)	Arg-**β-OH-Asp**-Ser-Pro-**N^{δ}-OH-N^{δ}-acetyl-Orn-N^{δ}-OH-N^{δ}-acetyl-Orn**	*Variovorax boronicumulans* NBRC 103145	BCUS01000000	Siderophore	[280]
**300**	Vibriobactin	VibF	NRP	AF287255 (AAG00566)/ QGF32328	Thr	*Vibrio cholerae* O395	CP000626, CP000627, CP045719	Siderophore	[281]
**301**	Vicibactin	VbsS/RHE_PF00457, VbsS/RHE_PF00457, VbsS/RHE_PF00457,	NRP	CP000138 (ABC94347)	N^{δ}-OH-Orn-N^{δ}-OH-Orn-N^{δ}-OH-Orn	*Rhizobium etli* CFN 42	None	Siderophore	[282]
**302**	Vioprolide	VioA, **VioB**, VioC, **VioD**	NRP	MH108942 (AWI62626, **AWI62627**, AWI62628, **AWI62629**)	Ala-Leu-**AZC/Pro-Cys**-Dhb-Pip/Pro-Thr-***N*-Me-Val**	*Cystobacter* sp. Cb vi35	None	Antifungal, antitumor, anti-inflammatory	[283]
**303**	Viscosin	ViscA, **ViscC**, ViscB	NRP	WP_015884800, **WP_012723756**, WP_012723757	Leu-Glu-**Thr-Val-Leu-Ser**-Leu-Ser-Ile	*P. aeruginosa* SBW25	NC_012660	Antibacterial	[284]
**304**	Viscosinamide	VsmA, **VsmB**, VsmC	NRP	MT771986 (QLY89362), MT749673 (**QLY89268**, QLY89269)	Leu-Gln-**Thr-Val-Leu-Ser**-Leu-Ser-Ile	*Pseudomonas* sp. U2W1.5	None	Antifungal	[210]
**305**	Vrginiafactins A−D	VifA, **VifB**	NRP	WP_100939442, **WP_100939443**	Leu-Leu-Gln-**Leu-Ser-Val/Ile-Leu-Leu**	*Pseudomonas* sp. QS1027	PHSU01000000	None	[285]
**306**	WAP-8294A1, A2 and A4	WAPS1, **WAPS2**	NRP	JN596952 (AEP18656, **AEP18655**)/ ROU07253, **ROU07252**	Ser-Asn-Ser-Gly-*N*-Me-Phe-Leu-Orn-**Glu-Asn-Trp-Orn-*N*-Me-Val**	*L. enzymogenes* OH11	RCTY00000000	Antibacterial	[286]
**307**	WBP-29479A1	WbpA, **WbpB**	NRP	ALN63820, **ALN64398**	Val-Arg-Ser-Gly-*N*-Me-Phe-Leu-Arg-**Glu-Val-Trp**	*Lysobacter antibioticus* ATCC 29479	CP013141	Antibacterial	[287]
**308**	WLIP	WlpA, **WlpB**, WlpC	NRP	JN982332 (AFJ23819), JN982333 (**AFJ23825**, AFJ23826)	Leu-Glu-**Thr-Val-Leu-Ser**-Leu-Ser-Ile	*P. putida* RW10S2	None	Antibacterial, swarming, biofilm formation	[288]

Table S1 Continued

No.	Product Name	ProteinName	Type	Protein ID (GenBank)	Recruited Substrates	Bacterial sources	Genome ID (GenBank)	Biological Evaluation	References
**309**	Xantholysins A−C	XtlA, **XtlB**, XtlC	NRP	KC297505 (AGM14925), KC297506 (**AGM14933**, AGM14934)	Leu-Glu-**Gln-Val-Leu-Gln-Ser-Val-Leu-Gl**n-Leu-Leu-Gln-Val/Ile	*P. putida* BW11M1	LSLE00000000	Insecticidal, antifungal, antibacterial	[289]
**310**	Xefoampeptides A−G	Xbed_01550	NRP	OTA20325	Leu-Leu-Val/Leu/Ala	*Xenorhabdus beddingii* DSM 4764	MUBK00000000	Antiprotozoal	[290−291]
**311**	Xenematide	XNC1_2713	NRP	CBJ90767	Thr-Trp/Phe-Trp/Phe	*X. nematophila* ATCC 19061	FN667742	Antibacterial, insecticidal	[291−292]
**312**	Xeneprotides A−C	Xekj_RS17945, **Xekj_RS17950**, Xekj_RS17955	NRP	WP_153044786, **WP_015835475**, WP_099111429	Thr-**Trp-Trp**-Pro	*Xenorhabdus* sp. KJ12.1	NZ_NJCW00000000	Antiprotozoal	[291]
**313**	Xenoamicins	XabAB/XDD1_2280, **XabC/XDD1_2281**, XabD/XDD1_2282	NRP	CDG17980, **CDG17981**, CDG17982	Pro-Gly/Ala-Val-Leu-Ile/Val-Thr-Val-**Val//Leu/Ile-Val**-Ala-Pro-Val	*Xenorhabdus mauleonii* DSM17909=FRM16	FO704550	Antiprotozoal	[293]
**314**	Xenoamicin III$_A$	Xekj_00203, **Xekj_00204**, Xekj_00205	NRP	PHM72992, **PHM72993**, PHM72994	Thr-Ala-Val-Leu-Leu-Thr-Thr-**Leu-Leu/Val**-Pro	*Xenorhabdus* sp. KJ12.1	NJCW01000000	Antiprotozoal	[187]
**315**	Xenocoumacin I	XcnA/XNC1_1711, **XcnK/XNC1_1701**	PK-NRP	CBJ89771, **CBJ89761**	Arg-**Leu**	*X. nematophila* ATCC 19061	FN667742	Antibacterial, antifungal	[294]
**316**	Xenortides A−D	XndA/XNC1_2300, **XndB/XNC1_2299**	NRP	CBJ90359, **CBJ90358**	*N*-Me-Leu/*N*-Me-Val-***N*-Me-Phe**	*X. nematophila* ATCC 19061	FN667742	Antibacterial, Antiprotozoal	[295]
**317**	Xenotetrapeptide	XtpS/XNC1_2022	NRP	CBJ90082	Val-Leu-Val-Val	*X. nematophila* ATCC 19061	FN667742	None	[296]
**318**	Xentrivalpeptides	Xekk_02473, **Xekk_02474**	NRP	PHM54490, **PHM54491**	Val-Thr-Phe-**Pro-Val-Val-Val**	*Xenorhabdus* sp. KK7.4	NJAH01000000	None	[187, 297]
**319**	Yersiniabactin	HMWP2/CH49_2040	PK-NRP	AJJ24457	Cys-Cys	*Yersinia enterocolitica* 8081	CP009846	Siderophore	[298]
**320**	YM-254890	YtfA, **YtfG**, YtfD, **YtfE**, YtfF	NRP	BBD84689, **BBD84695**, BBD84692, **BBD84693**, BBD84694	*β*-OH-Leu-***β*-OH-Leu-*N*,*O*-diMe-Thr**-Thr-***N*-Me-Dha**-Ala-*N*-Me-Ala	*Chromobacterium* sp. QS3666	None	Gq protein inhibitor	[299]
**321**	Zwittermicin A	ZmaO, **ZmaK**, ZmaB, **ZmaQ**	PK-NRP	FJ430564 (ACM79810, **AAR87760**, ACM79806, **ACM79812**)	Asn-**Ser**-Ala-**Leu-Met**	*B. cereus* UW85	JALJWA010000000	Antifungal	[300]

Table S2 Occurrence frequency of 36 proteinogenic and non-proteinogenic α-AAMs

Serial number	α-AAM-activating domains	Occurrence frequency	Frequency (Clustering)	Frequency (NRPSs/NRPS-PKSs)	Frequency (SNSs)
1	Leucine	224		/	
2	Isoleucine	104	525	/	117
3	Valine	197		/	
4	dehydrovaline	1	1	1	1
5	γ-hydroxyphenyl glycine	4	4	4	2
6	Alanine	155	155	73	46
7	α-aminobutyrate	2	2	2	2
8	Methionine	3	3	3	3
9	Glycine	93	93	72	21
10	Cysteine	74	74	42	21
11	Ornithine	89			28
12	Lysine	54	147	71	25
13	Cyclic lysine	4			4
14	α-amino-δ-nitropentanoic acid	1	1	1	1
15	Proline	71	71	63	34
16	Azetidine α-carboxylic acid	2	2	2	2
17	Arginine	37	37	31	29
18	Enduracididine	1	1	1	1
19	Homoarginine	2	2	1	1
20–21	Glutamate and glutamine	108	227	108	30
22–23	Aspartic acid and asparagine	119			24
24	β-cyano-alanine	1	1	1	1
25	α-aminoadipic acid	1	1	1	1
26–27	Threonine and dehydrobutyrine	213	213	127	12
28–29	Serine and dehydroalanine	201	201	132	16
30	Homoserine and homoserine lactone	13	13	11	2
31	Tyrosine	84			35
32	Phenylalanine	54	169	114	36
33	Tryptophan	31			25
34	α,γ-diaminobutyric acid	86	86	43	10
35	α,β-diaminopropionic acid	4	4	3	3
36	Histine	14	14	14	4

Table S3 SNSs located in Leu-activating domains and their NRPS/NRPS-PKS subunits

Substrates (Serial number)	Residues at positions within the GrsA Phe-activating domain										Subunit Name (Frequency)
	235	236	239	278	299	301	322	330	331	517	
Leu(1)	D	A	F	F	H	G	Q	V	C	K	TxlA(2), TxlB(2)
Leu(2)	D	A	F	F	L	G	C	V	F	K	SrfA-C
Leu(3)	D	A	F	F	L	G	I	T	F	K	PppD
Leu(4)	D	A	F	F	N	G	A	V	Y	K	AltM(2)
Leu(5)	D	A	F	I	L	G	A	V	C	K	Xbed_01550
Leu(6)	D	A	F	L	I	G	A	V	M	K	LugB
Leu(7)	D	A	F	M	L	G	M	V	F	K	LicA
Leu(8)	D	A	F	M	M	G	M	V	F	K	SrfA-A
Leu(9)	D	A	F	T	I	G	A	I	C	K	OctB
Leu(10)	D	A	I	Y	L	G	V	V	L	K	LedE
Leu(11)	D	A	L	F	M	G	A	T	F	K	CipF
N-Me-Leu(12)	D	A	L	L	M	G	A	V	C	K	XndA
N-Me-Leu(13)	D	A	L	V	L	A	V	S	I	K	RdpA, RdpB
Leu(14)	D	A	M	F	I	G	A	A	Y	K	EA58_10630
Leu(15)	D	A	M	F	L	G	C	T	F	K	EtlC
Leu(16)	D	A	M	H	L	G	C	T	F	K	HgddC
Hpg(1)	D	A	M	H	L	G	C	T	F	K	HgdA, HptC
Leu(17)	D	A	M	L	I	G	A	I	C	K	LesA,WAPS1
Leu(18)	D	A	M	L	I	G	A	V	C	K	WbpA
β-OH-Leu(19)	D	A	M	L	V	G	A	A	C	K	YtfA(2)
β-OH-Leu(20)	D	A	M	L	V	G	A	V	C	K	FrsD, FrsG, FrsA
Leu(21)	D	A	M	M	A	G	C	V	C	K	Xbed_01550
Leu(22)	D	A	Q	D	M	G	A	V	Q	K	AmiJ, XcnK
Leu(23)	D	A	Q	D	M	G	N	V	Q	K	UY9_14934
Leu(24)/Phe(13)	D	A	W	C	I	A	A	V	C	K	Plu3263/GxpS
Leu(25)	D	A	W	C	I	G	A	V	C	K	(11)*
Leu(26)	D	A	W	F	I	G	A	V	C	K	Xsze_03460
Leu(27)	D	A	W	F	L	G	A	T	F	K	Xekj_00203
Leu(28)	D	A	W	F	L	G	A	T	I	K	Xekj_00203
Leu(29)	D	A	W	F	L	G	H	V	V	K	(5)**
N-Me-Leu(29)	D	A	W	F	L	G	H	V	V	K	DidD
Leu(30)	D	A	W	F	L	G	M	T	F	K	XabC, Xekj_00204
Leu(31)	D	A	W	F	L	G	N	V	D	K	Ban(COW3)B
Leu(32)	D	A	W	F	L	G	N	V	V	K	(130)***

*: BicA, KolA(5), Plu3263/GxpS(3), PhpS, XtpS;

**: CpnD, BSL056_RS13665(2), BSL056_RS13660, VioA;

***: AniA/ClpA, AniB/ClpB(2), AniC/ClpC(2), ArfA, ArfB(2), ArfC, BacA, SubA, Ban(COW3)A, Ban(COW3)B, Ban(COW3)C, Ban(BW11P2)A, Ban(BW11P2)B(2), Ban(BW11P2)C, Bkm2, BogE, BrvE,BtD, BtF, CifA(2), CifB(3), DidH, EA58_10630(2), EtlA, EtlB(2), EtlC, GamA, GamB, GamC(2), GlpD, HptC(β-OH), HynA, IcoS(2), JesC, KorA(2), LicA, LicB, LokC, LokB(2), LokA(2), LybA(3), LybB, MassA, MassB, MassC, MchA-C, MchB-C(2), MchC-C, MchD1-C, MchD2-C, OfaA, OfaB, OfaC(2), P615_17235, P615_17230, PdmA, PdmB, PdmC,PekA, PekB(2),PoaA, PoaB(2), PoaC(2), PsoA, PsoB, PsoC, SwrA, SesA, SesB(2), SMU_1342, SrfA-A, SrfA-B, SteA, SteB(2), SteC(2), SyfA(2), SyfB(3), SypA, SypA, TaaB, TaaC, TaaD, TaaE, TnpC, ViscA, ViscC, ViscB, VifA(2), VifB(3), VsmA, VsmB, VsmC, WlpA, WlpB, WlpC, XtlA, XtlB(2), XtlC(2);

Table S3 Continued

Substrates	Residues at positions within the GrsA Phe-activating domain										Subunit Name
(Serial number)	235	236	239	278	299	301	322	330	331	517	(Frequency)
Leu(33)	D	A	W	F	L	G	Q	V	V	K	(5)*
Leu(34)	D	A	W	F	M	I	A	V	V	K	MynD
Leu(35)	D	A	W	I	I	G	A	I	V	K	OctC, PlpE(2)
Leu(36)	D	A	W	I	V	G	A	I	V	K	PE/PA/PD-PmxA
Leu(37)	D	A	W	L	I	G	A	I	C	K	MchS-B
Leu(38)	D	A	W	L	I	G	A	V	C	K	XBJ1_2367
Leu(39)	D	A	W	L	L	G	A	V	C	K	XabAB
Leu(40)	D	A	W	L	L	G	A	T	I	K	Xekj_00204
Leu(41)	D	A	W	Y	I	G	V	V	C	K	AmbS
Leu(42)	D	A	W	Y	L	G	N	V	V	K	AqmF, AqmG
Leu(43)	D	G	A	Y	T	G	E	V	V	K	GrsB(2), TycC
Leu(44)	D	G	F	F	L	G	V	V	Y	K	PE-PmxA
Leu(45)	D	G	F	F	V	G	G	V	F	K	BogD
Leu(46)	D	G	F	L	L	G	E	V	C	K	BogB, BrvB
Leu(47)	D	G	F	L	L	G	G	V	F	K	BtB
Leu(48)	D	G	F	L	L	G	L	V	Y	K	PB-PmxA, P615_17225
β-OH-Leu(49)	D	G	W	F	L	G	N	V	V	K	HrmC, HynB
Leu(50)	D	I	L	H	L	G	C	T	F	K	BgddA
Hpg(2)	D	I	L	H	L	G	C	T	F	K	BgdB, BptE
Leu(51)	D	L	Y	N	L	S	G	V	W	K	Xbed_01550
Leu(52)	D	V	F	Y	F	G	L	V	C	K	ZmaQ
Leu(53)	D	V	V	L	M	G	A	T	M	K	MXAN_3779
Leu(54)	D	V	W	L	L	G	A	V	I	K	BrL25_22185
Leu(55)	D	Y	F	T	F	G	L	I	I	K	RtcN

*: HepA/RzmA, LgrB, LgrC(2), LgrD

Table S4 SNSs located in Ile-activating domains and their NRPS/NRPS-PKS subunits

Substrates	Residues at positions within the GrsA Phe-activating domain										Subunit Name
(Serial number)	235	236	239	278	299	301	322	330	331	517	(Frequency)
Ile(1)	D	A	F	F	L	G	I	T	F	K	(4)*
Ile(2)	D	A	F	F	L	G	V	T	F	K	TubC, HynB
Ile(3)	D	A	F	F	L	G	V	T	Y	K	HolA(2)
Ile(4)	D	A	F	F	Y	G	I	T	F	K	PpsE, FenE
Ile(5)	D	A	F	T	Y	G	G	V	F	K	CmaA
Ile(6)	D	A	F	W	I	G	A	T	F	K	PhpS(5), Plu3123(2)
Ile(7)	D	A	I	Y	L	G	V	V	L	K	LedE
Ile(8)	D	A	L	F	I	G	G	T	Y	K	PsoC
Ile(9)	D	A	L	F	I	G	G	V	F	K	SMU_1340
Ile(10)	D	A	L	F	L	G	C	T	F	K	XtlC
Ile(11)	D	A	L	F	M	G	C	T	Y	K	CipD
Ile(12)	D	A	L	F	M	G	A	T	F	K	CipF
Ile(13)	D	A	L	W	M	G	G	T	F	K	RmyB
Ile(14)	D	A	L	W	M	G	G	V	F	K	BolH, GcnH
Ile(15)	D	A	L	W	W	G	G	V	F	K	CdeI
Ile(16)	D	A	M	F	I	G	A	T	F	K	SwrA
Ile(17)	D	A	M	F	I	G	G	T	F	K	SyfB
Ile(18)	D	A	M	F	L	G	C	T	F	K	CrpC, EtlC
Ile(19)	D	A	M	F	L	G	C	T	Y	K	(30)**
Ile(20)	D	A	M	F	L	G	G	T	F	K	OfaB
Ile(21)	D	A	M	F	M	G	C	T	F	K	NupC
Ile(22)	D	A	S	T	I	A	A	V	C	K	BSL056_RS13660
Ile(23)	D	A	W	F	L	G	M	T	F	K	XabAB, XabC
Ile(24)	D	A	W	F	L	G	N	V	V	K	(9)***
Ile(25)	D	A	W	F	L	G	V	T	F	K	VifB
Ile(26)	D	A	Y	F	F	G	I	T	Y	K	PttC
Ile(27)	D	A	Y	F	W	G	V	T	Y	K	LesB1
Ile(28)	D	A	Y	W	W	G	G	T	F	K	CpnD
Ile(29)	D	F	L	C	L	G	V	V	I	K	SpiDE1
N-Me-Ile(30)	D	G	A	F	L	G	I	I	L	K	NcyE
Ile(31)	D	G	F	F	F	G	V	V	Y	K	BacC
Ile(32)	D	G	F	F	L	G	V	V	F	K	(5)****
Ile(33)	D	G	F	F	L	G	V	V	Y	K	(10)*****
Ile(34)	D	G	F	Y	L	G	V	V	F	K	BreD
Ile(35）	D	G	L	Y	I	G	G	I	M	K	LgrA
Ile(36)	D	G	Y	F	L	G	V	V	Y	K	AqmD, AqmF
Ile(37)	D	I	L	H	L	G	C	T	F	K	BgddA
Ile(38)	D	Y	L	C	V	G	V	V	I	K	BhcD/TdpDE1

*: DidD, PppD, SAMN04488128_1011792, TriE

**: AniC/ClpC, ArfC(2), Ban(COW3)C, Ban(BW11P2)C, BrpA, BrpB, CrpB, GamB, GamC, JesA, LokA, LybB, MassB, MassC, NupB, PdmC, PekC(2), PoaC, SesC, SteC, TnpB, Txo1(3), Txo2, ViscB, VsmC, WlpC;

***: BrpA, EtlA, EtlB(2), EtlC, PsoB(2), TaaE, TnpC;

****: LatD, LicC, PbtB, PbtC, PlpE;

*****: BacA(2), SubA(2), SubC, BogC, BrvC(2), BtC, MarA

Table S5 SNSs located in Val-activating domains and their NRPS/NRPS-PKS subunits

Substrates (Serial number)	Residues at positions within the GrsA Phe-activating domain										Subunit Name (Frequency)
	235	236	239	278	299	301	322	330	331	517	
Val(1)	D	A	F	F	F	G	G	T	F	K	LugA
Val(2)	D	A	F	F	I	G	A	T	F	K	Bkm3
Val(3)	D	A	F	F	L	G	I	T	F	K	PppD
Val(4)	D	A	F	F	M	G	G	T	F	K	TtcB
Val(5)	D	A	F	F	V	G	G	T	F	K	KorD(2)
Val(6)	D	A	F	W	F	G	G	T	F	K	LugC(3)
Val(7)	D	A	F	W	I	G	A	T	F	K	KolA, LocC, GxpS
Val(8)	D	A	F	W	I	G	G	T	F	K	(20)*
Val(9)	D	A	F	W	I	G	G	V	F	K	AdmK
Val(10)	D	A	F	W	L	G	A	T	F	K	AltL(2)
Val(11)	D	A	F	W	L	G	C	T	F	K	FusA
Val(12)	D	A	F	W	L	G	C	V	F	K	SylC
Val(13)	D	A	F	W	L	G	G	T	F	K	(19)**
N-Me-Val(13)	D	A	F	W	L	G	G	T	F	K	TubC, VioD
Val(14)	D	A	I	Y	L	G	V	V	L	K	LedE
Val(15)	D	A	L	F	I	G	G	T	F	K	Zmn17
Val(16)	D	A	L	F	I	G	G	T	Y	K	PsoC
Val(17)	D	A	L	F	I	G	G	V	F	K	SMU_1340
Val(18)	D	A	L	F	L	G	C	T	F	K	XtlC
Val(19)	D	A	L	F	M	G	A	T	F	K	CipF
Val(20)	D	A	L	F	V	G	I	V	L	K	HamD
N-Me-Val(21)	D	A	L	L	M	G	A	V	C	K	XndA
N-Me-Val(22)	D	A	L	V	L	A	V	S	I	K	(7)***
Val(23)	D	A	L	W	I	G	G	T	F	K	(52)****
Val(24)	D	A	L	W	L	G	G	T	F	K	(5)*****
Val(25)	D	A	L	W	M	G	G	T	F	K	(11)******
Val(26)	D	A	L	W	M	G	G	T	L	K	TtmA(4)
Val(27)	D	A	L	W	M	G	G	V	F	K	BolH, GcnH
Val(28)	D	A	M	F	I	G	G	T	F	K	SyfB
Val(29)	D	A	M	F	M	G	G	T	F	K	CifB
Val(30)	D	A	M	F	L	G	G	T	F	K	OfaB, OfaC
Val(31)	D	A	W	F	I	G	G	T	F	K	FclJ
Val(32)	D	A	W	F	L	G	M	T	F	K	XabAB, 35XabC
Val(33)	D	A	W	F	L	G	V	T	F	K	VifB
Val(34)	D	A	W	L	L	G	A	T	I	K	Xekj_00204

*: EpyD(3), GrsB(2), HepA/RzmA, HolA,KolA/Plu2670(5), LicB, PlpE, SrfA-B, TycC, Xsze_03460, XtpS(3);

**: AqmF, AqmG(2), AusA, BtaE, CesB(3), DepD(2), LgrB, LgrC(2), PbtA, PbtB, PbtC, SAMN04488128_1011792, TriD, TriE;

***: RdpA, RdpB(2), Pb62B, RdpC(3);

****: BrpA, BrpB, BrpC, BrpC, CipB, CipD, CipE(2), CrpA(2), CrpB, CrpC, JesA, JesB, JesC, NupA, NupB, PdmB, NupC(2), PsoB, SesA, SesB(3), SypA-Psyr_2614, SypB-Psyr_2615, SypC-Psyr_2616, SypA-PsyrB_13255(2), SypB-PsyrB_13260(2), SypC-PsyrB_13265, SypA-PsyrH_12990(2), SypB-PsyrH_12985, SypC-PsyrH_12980(2), TttA(4), TnpA(2), TnpB, TnpC, TaaB, TaaC(2), TaaD, ViscC, VsmB;

*****: CmxB, MXAN_3779, TtcB(3)

******: BraA, BraB, EtlB(2), EtlC, RmyB, ThfA(2), WlpB, XtlB(2);

Table S5 Continued

Substrates	Residues at positions within the GrsA Phe-activating domain										Subunit Name
(Serial number)	235	236	239	278	299	301	322	330	331	517	(Frequency)
Dhv(1)	D	A	W	W	I	G	G	T	F	K	GlpE
Val(35)	D	A	W	W	L	G	G	T	F	K	(9)*
Val(36)	D	A	Y	F	W	G	V	T	Y	K	LesB
Val(37)	D	A	Y	W	L	G	G	T	F	K	PttB, WbpB
Val(38)	D	A	Y	W	W	G	G	T	F	K	CpnD, WbpA
N-Me-Val(38)	D	A	Y	W	W	G	G	T	F	K	WAPS2
Val(39)	D	F	L	C	L	G	V	V	I	K	SpiDE1
Val(40)	D	F	L	Q	V	G	I	I	F	K	AntB
Val(41)	D	G	F	F	A	G	G	V	F	K	BogE, BrvE, BtF
Val(42)	D	G	F	F	L	G	V	V	F	K	PlpE
Val(43)	D	G	F	F	L	G	V	V	Y	K	BogC
Val(44)	D	G	F	F	V	G	G	V	F	K	(8)**
Val(45)	D	G	G	Q	I	A	G	V	Y	K	SylD
Val(46)	D	G	L	Y	I	G	G	I	M	K	LgrA
Val(47)	D	G	M	H	N	V	G	I	I	K	MXAN_3779
Va(48)	D	G	Y	F	L	G	V	V	F	K	AqmG
Val(49)	D	I	L	H	L	G	C	T	F	K	BgddA
Val)(50)	D	L	Y	N	L	S	G	V	W	K	Xbed_01550
Val(51)	D	V	F	W	I	G	G	T	F	K	FenC, PpsC
Val(52)	D	V	F	W	L	G	G	T	F	K	PhnE
Val(53)	D	Y	L	C	V	G	V	V	I	K	BhcD/TdpDE1
Val(54)	D	Y	L	S	N	A	I	T	Y	K	NcyF

*: XabAB(2), XabC, XabD, Xekj_00203, Xekk_02473, Xekk_02474(3);

**: BogC, BogD, BrvC, BrvD(2), BtC(2), BtD;

Table S6 SNSs located in Ala- and Met-activating domains and their NRPS/NRPS-PKS subunits

Substrates	Residues at positions within the GrsA Phe-activating domain										Subunit Name
(Serial number)	235	236	239	278	299	301	322	330	331	517	(Frequency)
Ala(1)	D	A	W	H	I	S	L	M	D	K	CpbI
Ala(2)	D	F	P	N	F	C	I	V	Y	K	FusA
Ala(3)	D	I	A	Q	S	T	L	V	W	K	HlaA
Ala(4)/Gly(3)	D	I	F	N	N	A	L	I	Y	K	XabAB
Ala(5)	D	I	L	E	L	A	I	L	C	K	UY9_14934
Ala(6)	D	I	L	Q	I	G	Q	I	Y	K	PaeF(3)
Ala(7)	D	I	L	Q	L	G	V	I	W	K	BtaK
Ala(8)/Ser(11)	D	I	N	Q	L	S	M	I	W	K	MegI
Ala(9)/Gly(17)	D	I	V	Q	L	G	L	V	Y	K	PppA
Ala(10)	D	L	F	N	C	A	L	T	Y	K	FbnP-FbnQ
Ala(11)	D	L	F	N	N	A	L	T	Y	K	(18)*
Ala(12)	D	L	F	N	N	A	L	V	Y	K	BraB, FbnO, ThfB
Ala(13)/Ser(15)	D	L	W	N	M	G	E	V	W	K	Arg3
Ala(14)	D	L	W	N	N	A	L	T	Y	K	(6)**
Ala(15)/Leu(51)/Val(50)	D	L	Y	N	L	S	G	V	W	K	Xbed_01550
Ala(16)	D	L	Y	N	N	A	L	I	Y	K	HepA/RzmA
Ala(17)	D	L	Y	N	N	A	L	T	Y	K	(73)***
Ala(18)	D	L	Y	N	N	G	L	V	Y	K	Xekj_00203
Ala(19)	D	M	P	Q	L	G	M	V	W	K	ThaiH
Ala(20)	D	T	F	W	L	G	G	T	F	K	SpiDE1
Ala(21)	D	V	A	N	F	A	I	I	Y	K	LgrB
Ala(22)	D	V	A	N	I	A	L	I	Y	K	CesA(3)
Ala(23)	D	V	D	D	L	A	A	V	Y	K	MXAN_3779
Ala(24)	D	V	F	H	F	S	L	I	H	K	MarB
Ala(25)	D	V	F	S	V	A	I	V	Y	K	FrsF, YtfF
N-Me-Ala(25)	D	V	F	S	V	A	I	V	Y	K	FrsF, YtfF
Ala(26)	D	V	F	V	N	A	L	T	Y	K	PelA1
Ala(27)	D	V	F	W	L	G	G	T	F	K	TriE
Ala(28)	D	V	F	W	T	G	G	I	F	K	ClbB
Ala(29)	D	V	G	W	I	A	G	I	V	K	GlbC
Ala(30)	D	V	G	W	I	T	G	I	V	K	Plu1880
Ala(31)/Abu(1)	D	V	L	F	L	G	V	V	A	K	Arg2
Ala(32)	D	V	L	N	L	A	M	V	I	K	ZmaB
Ala(33)/Abu(2)	D	V	M	F	I	G	I	V	A	K	MchS-C
Ala(34)	D	V	P	N	F	A	L	V	F	K	SulM
Ala(35)	D	V	S	N	M	A	I	I	Y	K	BaeN

*: AqmE, BtaN, BraA, BraB, CpbK, CmdC, HynA, KolA, KrsB, MxaA, MYSTI_04320, RmyB, SacA, SafB, BSL056_RS13665, Txo2, ThfA, ThfB;

**: PvdI/Pfl01_1845, PvdI/BG51_RS09210, PvdK(2)/BG51_RS09170, PvdJ/PFL_4094, PvdD/PFL_4093, APS14_ 17630;

***: AmbB, XBJ1_2367, BrpA(2), BrpB, BrpC(2), BgdA-BgdB, BptE, CipA(2), CipA-CipB(2), CipD, CipE, CipF, CrpA(3), CrpB, CrpC(3), HepA/RzmA(2), HolA, JesA, JesB(3), JesC(2), LgrB, NupA(4), NupB, NupC(2), SypA-Psyr_2614, SypB-Psyr_2615(3), SypC-Psyr_2616(4), SypA-PsyrB_13255, SypB-PsyrB_13260(2), SypC-PsyrB_13265(4), SypA-PsyrH_12990(3), SypB-PsyrH_12985(2), SypC-PsyrH_12980(2), SypC-PsyrH_12980(2), TnpA(3), TnpB3, TnpC(3), XabD, BgddA

Table S6 Continued

Substrates	Residues at positions within the GrsA Phe-activating domain										Subunit Name
(Serial number)	235	236	239	278	299	301	322	330	331	517	(Frequency)
Ala(36)	D	V	T	N	F	A	L	I	Y	K	SevB
Ala(37)	D	V	W	C	T	A	A	I	I	K	MchD1-B
Ala(38)	D	V	W	C	T	A	A	V	I	K	MchD2-B
Ala(39)	D	V	W	H	F	S	L	I	E	K	SMU_1342, TxlA, TxlB
Ala(40)	D	V	W	H	I	S	L	I	E	K	CpbI
Ala(41)	D	V	W	H	L	S	L	I	E	K	VioA
Ala(42)	D	V	W	M	S	A	G	I	I	K	MchB-C
Ala(43)	D	V	W	V	I	A	A	V	I	K	(3)*
Ala(44)	D	V	W	V	L	A	A	I	I	K	(3)**
Ala(45)	D	V	W	V	T	A	A	I	I	K	MchA-C
Ala(46)	D	V	Y	N	M	A	I	V	Y	K	AqmF
Met(1)	D	V	F	Y	L	G	G	V	F	K	BhcD/TdpDE1
Met(2)	D	V	F	Y	L	G	G	V	C	K	ZmaQ
Met(3)	D	V	W	Y	L	G	G	I	C	K	BN712_00198

*: MchA-B, MchB-B, MchC-B;

**: MchD1-C, MchD2-C, MchS-C

Table S7 SNSs located in Gly-activating domains and their NRPS/NRPS-PKS subunits

Substrates	Residues at positions within the GrsA Phe-activating domain										Subunit Name
(Serial number)	235	236	239	278	299	301	322	330	331	517	(Frequency)
Gly(1)	D	I	A	N	L	C	I	I	Y	K	LgrA
Gly(2)	D	I	F	H	M	G	M	I	W	K	PamH
Gly(3)/Ala(4)	D	I	F	N	N	A	L	I	Y	K	XabAB
Gly(4)	D	I	L	L	L	G	C	V	W	K	AmoH
Gly(5)	D	I	L	Q	I	G	L	I	W	K	(4)***
Gly(6)	D	I	L	Q	I	G	M	V	W	K	LybB, Odl2
Gly(7)	D	I	L	Q	I	G	V	I	W	K	PttB
Gly(8)	D	I	L	Q	L	G	L	I	W	K	(28)*
N-Me-Gly(8)	D	I	L	Q	L	G	L	I	W	K	MscH
Gly(9)	D	I	L	Q	L	G	L	V	W	K	(7)**
Gly(10)	D	I	L	Q	L	G	M	I	W	K	BaeJ, CroI, FJ695_03635, Ta-1
N-Me-Gly(10)	D	I	L	Q	L	G	M	I	W	K	AjuL
MOX-Gly(10)	D	I	L	Q	L	G	M	I	W	K	CtaG, FtaE, MelG, MtaG
Gly(11)	D	I	L	Q	L	G	M	V	W	K	PabJ
N-Me-Gly(11)	D	I	L	Q	L	G	M	V	W	K	Arg2
Gly(12)	D	I	L	Q	L	G	V	I	W	K	(13)[†‡]
N-Me-Gly(12)	D	I	L	Q	L	G	V	I	W	K	CdeK
Gly(13)	D	I	L	Q	L	G	V	V	W	K	(6)*****
Gly(14)	D	I	L	Q	M	G	M	V	W	K	(6)****
Gly(15)	D	I	L	Q	W	G	L	I	W	K	JagC
Gly(16)	D	I	L	Q	Y	G	M	I	W	K	LocB(2)
Gly(17)/Ala(9)	D	I	V	Q	L	G	L	V	Y	K	PppA
Gly(18)	D	I	W	Q	L	I	A	D	S	K	BksF, OcfE
Gly(19)	D	L	W	N	N	C	L	T	Y	K	PputGB1_4084
Gly(20)	D	L	Y	N	N	C	L	V	Y	K	LgrD
Gly(21)	D	V	W	Q	F	G	F	I	Y	K	KrsB

*: AvCA_25580, DhbF(3), ClbJ, CorI, Daci_4754, EdeI, FbnP-FbnQ, FbnP-FbnQ, GlpE, Bat2, LgaA, LgaF, LesA, MscH, MscI, MxnI, PedF, PblD, PvdJ(II), PvdI/BG51_RS09210, PvdJ(1)/BG51_RS09205, APS14_17630, APS14_17620, PFLU2543/PvdI, DCC84_23185, SacB;

**: Bkm3, CdeI, CdeJ, SMU_1339, DCC84_23175, SafA, WbpA;

***: GrbI, CUJ87_10420, PaxA, PP_4219;

****: BreC, BreD, LatC, LatD(2), TriD;

*****: AlmB, Alb5, Arg3, CipC, CipD, MegH, NunD, PxnM, PvdD(III), RmyB, RizB, ThxC2, ThxA2;

******: AlmA, Alb4, AdmP, CucJ, HtbG, WAPS1

Table S8 SNSs located in Cys-activating domains and their NRPS/NRPS-PKS subunits

Substrates	Residues at positions within the GrsA Phe-activating domain										Subunit Name
(Serial number)	235	236	239	278	299	301	322	330	331	517	(Frequency)
Cys(1)	D	L	Y	N	L	S	L	I	W	K	(24)*
N-Me-Cys(1)	D	L	Y	N	L	S	L	I	W	K	(3)**
Ox-Cys(1)	D	L	Y	N	L	S	L	I	W	K	(7)***
Cys(2)	D	L	Y	N	M	S	L	I	W	K	(4)****
N-Me-Cys(2)	D	L	Y	N	M	S	L	I	W	K	MicH
Ox-Cys(2)	D	L	Y	N	M	S	L	I	W	K	Arg3, FbnO, SidM
Cys(3)	D	L	Y	N	M	S	M	I	W	K	(6)*****
N-Me-Cys(3)	D	L	Y	N	M	S	M	I	W	K	BGP74_RS13430
N-Me-Cys(4)	D	L	Y	N	F	S	M	I	W	K	ACZ75_02210(2), MicC(2)
Cys(5)	D	L	Y	N	L	S	M	I	W	K	Irp2
N-Me-Cys(5)	D	L	Y	N	L	S	M	I	W	K	Irp2
Cys(6)	D	F	A	E	M	S	L	I	W	K	SpiDE1
Cys(7)	D	G	E	A	T	G	G	I	T	K	PhmB
Cys(8)	D	L	F	N	L	S	L	I	W	K	LugD, PchE-PA14
Cys(9)	D	L	F	N	M	S	L	I	W	K	Irp1
Cys(10)	D	L	F	E	M	S	L	I	W	K	BhcD/TdpDE1, DepD
Cys(11)	D	L	Q	N	M	S	L	I	W	K	TubD
Cys12)	D	L	W	N	L	S	L	I	W	K	AntB
Ox-Cys(12)	D	L	W	N	L	S	L	I	W	K	TugD,TgaC
Cys(13)	D	L	Y	D	M	S	M	I	W	K	EqbE(3)
Cys(14)	D	L	Y	N	I	S	A	I	W	K	SMU_1340
Ox-Cys(15)	D	L	Y	N	L	A	L	V	W	K	AjuD
Cys(16)	D	L	Y	N	M	S	L	V	W	K	CtaC, MelC
Ox-Cys(17)	D	L	Y	N	W	S	L	I	W	K	FtaB

*: Alb5, UY9_14934, NrsC(3), BacA, SubA, BlmIV, ClbJ, EtuA3, DepA, Hmo7, MarB(4), PtzF, Xsze_03167(2), SpiA, HolA(2), BGP74_RS13415, VioB;

**: PchF/PFL_3492, PchF-PA14, BGP74_RS13430;

***: AlmA, Alb4, CtaD, EpoB, EPOS P, MelD, MtaD;

****: PchE/PFL_3493, SidI, MtaC, PppB;

*****: AlmB, AngR+AngM, Xsze_03168(2), HMWP2(2)

Table S9 SNSs located in Orn- and ANPA--activating domains and their NRPS/NRPS-PKS subunits

Substrates	Residues at positions within the GrsA Phe-activating domain										Subunit Name
(Serial number)	235	236	239	278	299	301	322	330	331	517	(Frequency)
N^{δ}-OH-Orn(1)	D	A	E	A	A	G	G	I	S	K	OrbI
N^{δ}-OH-N^{δ}-acetyl-Orn(2)	D	G	E	A	C	G	G	V	T	K	(4)*
N^{δ}-OH-N^{δ}-formyl-Orn(2)	D	G	E	A	C	G	G	V	T	K	PvdJ(II)
cyclo-N^{δ}-OH-Orn(2)	D	G	E	A	C	G	G	V	T	K	PP_4219
cyclo-N^{δ}-OH-Orn(3)	D	G	E	A	V	G	G	V	T	K	Daci_4753, PfbK
cyclo-N^{δ}-OH-Orn(4)	D	G	E	C	C	G	G	V	T	K	(5)**
N^{δ}-OH-N^{δ}-acetyl-Orn(5)	D	G	E	C	T	G	G	I	T	K	PfbK, Var7, VarJ(2)
N^{δ}-OH-N^{δ}-formyl-Orn(5)	D	G	E	C	T	G	G	I	T	K	Var7
N^{δ}-OH-N^{δ}-acetyl-Orn(6)	D	G	E	C	T	G	G	V	T	K	AbsE(2), AbsF
Orn(7)	D	G	E	D	H	G	T	V	T	K	(4)***
N^{δ}-OH-N^{δ}-butyryl-Orn(8)	D	G	E	G	S	G	G	V	T	K	CucH, TaiG
N^{δ}-OH-N^{δ}-formyl-Orn(8)	D	G	E	G	S	G	G	V	T	K	Vapar_3743
cyclo-N^{δ}-OH-Orn(8)	D	G	E	G	S	G	G	V	T	K	TaiG
N^{δ}-OH-Orn(9)	D	G	E	S	S	G	G	M	T	K	VbsS(3)
N^{δ}-OH-N^{δ}-formyl-Orn(10)	D	G	E	V	C	G	G	V	T	K	(9)****
cyclo-N^{δ}-OH-Orn(11)	D	G	E	V	V	G	G	V	T	K	Hsero_2343
N^{δ}-OH-N^{δ}-formyl-Orn(12)	D	G	E	Y	T	G	G	I	T	K	MbaJ, OrbJ
N^{δ}-OH-N^{δ}-formyl-Orn(13)	D	L	W	G	M	G	A	V	N	K	RHA1_ro02319(2)
Orn(14)/Lys(20)	D	M	E	D	V	G	S	V	D	K	PamD
Orn(15)	D	M	E	N	M	G	A	V	N	K	BrmB, ThaA
cyclo-N^{δ}-OH-Orn(16)	D	M	E	N	M	G	L	I	N	K	HtbG
Orn(17)	D	M	E	N	S	G	A	V	N	K	Odl2
Orn(18)	D	S	A	E	L	G	T	V	D	K	HcsG
Orn(19)	D	S	D	D	G	G	L	V	D	K	TnbF(3)
Orn(20)	D	S	G	P	S	G	A	V	D	K	BogB, BrvB, BtB
Orn(21)	D	T	E	D	M	G	L	V	D	K	WAPS2
Orn(22)	D	T	E	D	M	G	Y	V	D	K	WAPS1
N^{δ}-OH-Orn(23)/ANPA(1)	D	V	E	T	L	G	G	I	S	K	MbaI
Orn(24)	D	V	G	E	I	G	S	I	D	K	(10)*****
Orn(25)	D	V	G	E	I	G	S	V	D	K	BacB, SubB, PbtA(2)
Orn(26)	D	V	G	E	V	G	S	I	D	K	FenA, HASF
N^{δ}-OH-N^{δ}-nitroso-Orn(27)	D	V	H	R	T	G	L	V	A	K	(8)******
N^{δ}-OH-N^{δ}-formyl-Orn(28)	D	V	W	N	I	G	L	I	H	K	CroH, Daci_4753
N^{δ}-OH-Orn(28)	D	V	W	N	I	G	L	I	H	K	Vapar_3746

*: PvdI/Pfl01_1845, APS14_17630, AvCA_25560, DCC84_23185

**: DCC84_23175, PvdJ(2)/PT02606, PputGB1_4083, PvdK(2)/BG51_RS09170, APS14_17620;

***: PblL, PvdI(II), PP_4221, PvdI(14)/Pfl01_1845;

****: PvdI(III), PvdD(III), PvdI(II), PvdI/PA2402, PvdJ/PA2400, PvdI/PFL_4095, PvdD/PFL_4093, PFLU2543/PvdI, PFLU2544/PvdJ;

*****: BreC(3), PpsA, GrsB(2), LatC(3), TycC;

******: GrbI, GrbJ, CUJ87_10420, CUJ87_10415, MegI, MegI, PblD, PblE

Table S10 SNSs located Lys (including Pip)-activating domains and their NRPS/NRPS-PKS subunits

Substrates	Residues at positions within the GrsA Phe-activating domain										Subunit Name
(Serial number)	235	236	239	278	299	301	322	330	331	517	(Frequency)
Lys(1)	D	A	E	D	H	G	T	V	E	K	PabB, PabG
Lys(2)	D	A	E	D	I	G	T	I	I	K	CphC
Lys(3)	D	A	E	D	I	G	T	V	I	K	AvCA_21190
Lys(4)	D	A	E	D	I	G	T	V	V	K	AmoF(2), MxcG
Lys(5)	D	A	E	D	I	G	T	V	S	K	GlpD
Lys(6)	D	A	E	D	N	G	T	V	S	K	PvdI/PFL_4095, PvdD/PFL_4093
Lys(7)	D	A	E	S	I	G	S	V	C	K	BacB, SubB
Lys(8)	D	A	E	S	I	G	T	I	I	K	SesC, TaaE
Lys(9)	D	A	E	S	V	G	T	V	I	K	AmbS
Lys(10)/Arg(11)	D	A	G	D	I	G	A	I	T	K	PabG
Lys(11)	D	A	G	P	S	G	A	V	D	K	(6)*
Lys(12)	D	G	E	D	H	G	T	V	V	K	(9)**
β-OH-Lys/Lys(13)	D	I	E	S	I	G	T	I	V	K	Odl4
β-OH-Lys/Lys(14)	D	I	E	S	I	G	T	V	L	K	Odl4
Lys(15)	D	I	E	T	I	G	T	V	T	K	Odl1
N-Me-Pip(16)	D	I	Q	Y	I	A	Q	V	V	K	TubB
Pip(17)/Pro(1)	D	I	Q	Y	Y	A	Q	V	V	K	VioC
γ-OH-Lys(18)	D	L	G	D	V	G	S	I	D	K	GlbC
Lys(18)	D	L	G	D	V	G	S	I	D	K	Plu1880
Lys(19)	D	L	K	N	V	G	S	D	V	K	ThaA
Lys(20)/Orn(14)	D	M	E	D	V	G	S	V	D	K	PamD
N-Me-Pip(21)	D	N	E	S	G	G	T	V	A	K	BenD
Lys(22)/Arg(16)	D	P	E	D	I	G	G	V	E	K	PamA
Lys(23)	D	S	E	D	M	G	L	I	D	K	FreF(3), CbsF(3)
Lys(24)	D	T	S	D	I	G	S	V	I	K	PaxB, PaxC(3)
Lys(25)	D	T	S	D	I	G	S	V	T	K	PaxB
Lys(26)	D	V	G	D	V	G	S	I	D	K	PbtB, PbtC
Pip(27)/Pro(21)	D	V	Q	F	V	A	Q	V	V	K	CmxB
Lys(28)	D	V	S	E	T	G	V	C	E	K	SylD
Lys(29)	D	T	S	D	I	G	T	V	I	K	PaxB

*: PSPPH_1923, PSPTO_2147, PvdI/PT02609, PputGB1_4086, PputGB1_40830, PvdJ/PA2400, PvdI/BG51_RS09210, PFLU2543/PvdI, PFLU2544/PvdJ;

**: BogC, BogD, BrvC, BrvD, BtC, BtE

Table S11 SNSs located Pro- and AZC-activating domains and their NRPS/NRPS-PKS subunits

Substrates (Serial number)	Residues at positions within the GrsA Phe-activating domain										Subunit Name (Frequency)
	235	236	239	278	299	301	322	330	331	517	
Pro(1)/Pip(17)	D	I	Q	Y	Y	A	Q	V	V	K	VioC
Pro(2)	D	I	W	Y	I	S	L	L	G	K	EpyD
Pro(3)	D	L	F	Y	I	A	F	V	C	K	HapI
Pro(4)	D	L	F	Y	L	A	L	V	C	K	EpyB
Pro(5)	D	L	L	Y	L	A	L	V	C	K	DkxA, Leu5, PltF, PynC
Pro(6)	D	M	E	N	V	S	H	V	V	K	Odl2
Pro(7)	D	M	L	V	M	G	V	F	A	K	Pys
Pro(8)/AZC(1)	D	M	Q	L	V	S	Q	Q	V	K	AzeB
Pro(9)	D	V	H	H	I	A	C	V	Y	K	PxnH
Pro(10)/AZC(2)	D	V	Q	C	L	S	E	V	T	K	VioB
Pro(11)	D	V	Q	F	A	A	H	V	A	K	CpnD
Pro(12)	D	V	Q	F	A	A	Q	V	V	K	DidC, DidI
Pro(13)	D	V	Q	F	I	A	H	V	I	K	MchB-C
Pro(14)	D	V	Q	F	I	A	H	V	V	K	(15)*
Pro(15)	D	V	Q	F	I	A	H	V	T	K	TaaA
Pro(16)	D	V	Q	F	I	A	Q	V	V	K	(2)***
Pro(17)	D	V	Q	F	L	A	Q	V	V	K	Var6, VarI
Pro(18)	D	V	Q	F	S	A	H	V	V	K	BraB
Pro(19)	D	V	Q	F	V	A	H	V	I	K	P615_17235
Pro(20)	D	V	Q	F	V	A	H	V	V	K	(4)**
Pro(21)/Pip(27)	D	V	Q	F	V	A	Q	V	V	K	CmxB
Pro(22)	D	V	Q	H	A	A	H	V	A	K	LedF
Pro(23)	D	V	Q	H	I	A	H	V	T	K	BptE
Pro(24)	D	V	Q	H	I	A	Q	V	V	K	PppB
Pro(25)	D	V	Q	S	I	A	H	V	V	K	GrsB(2), TycB
Pro(26)	D	V	Q	Y	A	A	H	V	V	K	CdeJ
Pro(27)	D	V	Q	Y	I	A	H	V	A	K	BrpA
Pro(28)	D	V	Q	Y	I	A	H	V	C	K	BptE
Pro(29)	D	V	Q	Y	I	A	H	V	T	K	(6)****
Pro(30)	D	V	Q	Y	I	A	H	V	V	K	(6)*****
Pro(31)	D	V	Q	Y	I	S	Q	V	I	K	PxaA
Pro(32)	D	V	Q	Y	V	A	H	V	T	K	CipA
Pro(33)	D	V	Q	Y	V	A	H	V	V	K	BgddA, PppD
Pro(34)	D	V	Q	Y	Y	T	L	V	C	K	NpsB(2)

*: BmyD-B, BraB, FenD, PpsD, ItuA-B, SMU_1341c, MycB, MchA-C, MchC-C, ERIC2_c18760, ThfB7, Xekj_RS17955, XabAB, XabD, Xekk_02474;

**: P615_17230, CmxB, E7V67_01245, PpgJ;

***: LeuE, SAMN04488128_1011792;

****: BgdA, BgddA, CrpA, NupA, SesA, TnpA;

*****: BgdB, JesA, PbtB, SypA-Psyr_2614, SypA-PsyrB_13255, SypA-PsyrH_12990

Table S12 SNSs located Arg-, End- and Har- activating domains and their NRPS/NRPS-PKS subunits

Substrates	Residues at positions within the GrsA Phe-activating domain										Subunit Name
(Serial number)	235	236	239	278	299	301	322	330	331	517	(Frequency)
Ar(1)	D	A	D	D	S	G	A	V	T	K	GlmI
Arg(2)	D	A	D	D	V	G	L	V	D	K	HtbG
Arg(3)	D	A	E	D	I	G	A	I	T	K	PedH, PvdI/PA2402
Arg(4)	D	A	E	D	I	G	A	I	S	K	PfbJ
Arg(5)	D	A	E	D	L	G	A	I	T	K	VarF, Var3
Arg(6)	D	A	E	D	L	G	F	V	D	K	VabF
Arg(7)	D	A	E	D	V	G	T	V	S	K	Bkm2
Arg(8)	D	A	E	D	V	G	V	I	G	K	FJ695_03625
Arg(9)	D	A	E	N	I	G	A	I	T	K	AvCA_25570
Arg(10)	D	A	E	S	V	G	A	V	D	K	CpbI
Arg(11)/Lys(10)	D	A	G	D	I	G	A	I	T	K	PabG
Arg(12)	D	G	E	D	H	G	A	V	T	K	Daci_4753
α, *β*-dehydro-Arg(13)	D	I	G	D	L	G	I	I	D	K	Odl4
Arg(14)	D	I	S	N	I	G	A	I	T	K	XBJ1_2367
Arg(15)	D	P	E	N	I	G	H	V	D	K	XcnA
Arg(16)/Lys(22)	D	P	E	D	I	G	G	V	E	K	PamA
Arg(17)	D	P	M	E	S	G	A	I	Q	K	BrL25_22185
Arg(18)	D	S	E	D	V	G	A	V	D	K	PvdJ/Pfl01_1846
Arg(19)	D	T	E	D	I	G	A	V	D	K	WbpA
Arg(20)	D	T	E	D	V	G	A	V	D	K	LesA
Arg(21)	D	T	E	D	V	G	C	V	D	K	LesA, WbpA
Arg(22)	D	V	A	D	V	G	A	I	D	K	PppB, SyrE
Arg(23)	D	V	E	E	I	G	A	I	T	K	HynA
Arg(24)	D	V	E	F	I	G	A	V	T	K	BicA(2)
Arg(25)	D	V	E	L	I	G	A	V	T	K	BicA(2)
Arg(26)	D	V	E	N	V	G	A	I	N	K	E7V67_01245, LybB
Arg(27)	D	V	E	S	I	G	G	V	T	K	Plu3123(2)
Har(1)	D	V	E	S	I	G	G	V	T	K	PdtS(2)
Arg(28)	D	V	G	D	I	G	A	V	T	K	AqmF
Arg29)	D	V	K	D	L	G	C	V	E	K	BrnE
End(1)	D	A	E	D	V	A	A	M	I	K	Txo2

Table S13 SNSs located Glu-activating domains and their NRPS/NRPS-PKS subunits

Substrates	Residues at positions within the GrsA Phe-activating domain										Subunit Name
(Serial number)	235	236	239	278	299	301	322	330	331	517	(Frequency)
Glu(1)	D	A	M	H	L	G	G	T	A	K	SulI
Glu(2)	D	A	K	D	I	G	V	V	D	K	BacA, SubA, PhnE
Glu(3)	D	A	K	D	L	G	V	V	D	K	(5)*
Glu(4)	D	A	Q	D	L	G	V	V	D	K	GlpC
Glu(4)/Gln(2)	D	A	Q	D	L	G	V	V	D	K	BraA
Glu(5)	D	A	W	H	F	G	G	V	D	K	FenA, FenC, PpsA, PpsC
Glu(6)	D	G	W	K	F	G	V	V	D	K	(6)**
Glu(7)	D	G	W	K	L	G	V	V	D	K	MassA, PoaA, ViscA
Glu(8)	D	L	T	K	L	A	A	V	T	K	CdeI, CdeJ
β-OH-Glu(8)	D	L	T	K	L	A	A	V	T	K	CdeK
Glu(9)	D	P	R	S	L	S	Q	M	A	K	HolA
Glu(10)	D	S	K	D	L	G	L	V	N	K	AmbE
Glu(11)	D	S	K	D	L	G	V	V	D	K	Bkm1, Bkm3
Glu(12)	D	V	W	H	F	G	R	I	N	K	(12)***
Glu(13)	D	V	W	H	V	G	S	I	G	K	LesB, WAPS2, WbpB

*: BmyD-B, BmyL-B, BacB, SrfA-A, Tri-D;

**: EtlA, OfaA, PsoA, SteC, WlpA, XtlA;

***: AvCA_25650, PSPPH_1911, PSPTO_2135, PvdL/PT01550, PputGB1_3809, PP_4243, PvdL/PA2424, PvdL/Pfl01_3940, PvdL/BG51_RS20610, PvdL/PFL_4189, PvdL/APS14_09630, Pflu6137;

Table S14 SNSs located Gln-activating domains and their NRPS/NRPS-PKS subunits

Substrates	Residues at positions within the GrsA Phe-activating domain										Subunit Name
(Serial number)	235	236	239	278	299	301	322	330	331	517	(Frequency)
Gln(1)	D	A	E	Y	L	G	T	V	T	K	PblD
Gln(2)	D	A	Q	D	L	G	V	V	D	K	(8)*
Gln(2)/Glu(4)	D	A	Q	D	L	G	V	V	D	K	BraA
Gln(3)	D	A	V	Q	M	G	C	V	D	K	LocB
Gln(4)	D	A	W	H	F	G	S	V	E	K	FenD, PpsD
Gln(5)	D	A	W	Q	C	G	F	I	E	K	MchC-C
Gln(6)	D	A	W	Q	C	G	L	I	D	K	(7)**
Gln(7)	D	A	W	Q	F	G	L	I	D	K	(11)***
Ox-Gln(8)	D	A	W	Q	F	G	L	I	N	K	IgiD(2)
Gln(9)	D	A	W	Q	F	G	L	I	Q	K	LesB
Gln(10)	D	A	W	Q	F	G	L	V	D	K	PppC
Gln(11)	D	A	W	Q	F	G	V	V	D	K	(5)****
Gln(12)	D	A	W	Q	L	G	L	I	D	K	(3)*****
Gln(13)	D	A	W	Q	V	G	V	I	H	K	BlmVII
Gln(14)	D	A	W	Q	V	G	V	V	D	K	(12)******
Gln(15)	D	G	W	Q	V	G	V	V	D	K	PekA, VsmA
Gln(16)	D	P	S	H	M	G	S	I	T	K	CpnD
Gln(17)	D	S	W	D	I	G	L	I	G	K	EdeJ
Gln(18)	D	V	Q	D	L	G	V	V	D	K	KrsC

*: AmbS, ItuA-B, KrsC, LicA, MycB, ERIC2_c18760, Txo1, ThfA;

**: MchA-C, MchB-C, MchD1-C, MchD2-C, MchS-C, PvdJ(III), PvdD(III);

***: BgdA, BgddA, DidA(4), JagC, SesA, SesB, TycC, TaaD;

****: Ban(BW11P2)B, GamB(2), PdmA, SteC;

*****: PvdD/BG51_RS09160, APS14_17625, DCC84_23180;

******: CifA, CifB, EtlB(3), PsoB, SyfA, VifA, XtlB(3), XtlC

Table S15 SNSs located Asp-/AMA-and Asn/Cya-3-activating domains and their NRPS/NRPS-PKS subunits

Substrates	Residues at positions within the GrsA Phe-activating domain									Subunit Name
(Serial number)	235	236	239	278	299	301	322	330	331	517	(Frequency)
Asp(1)	D	A	S	T	A	T	M	R	S	K	BolO
β-OH-Asp(2)	D	I	T	K	I	G	H	V	G	K	PabI(2)
Asp(3)	D	L	E	A	A	G	G	I	S	K	PhmA
Asp(4)	D	L	T	K	I	G	H	I	G	K	CucG, LocB(2)
β-OH-Asp(5)	D	L	T	K	I	G	H	V	G	K	(10)*
β-OH-Asp(6)	D	L	T	K	L	G	H	V	G	K	HcsH, MegI
Asp(7)	D	L	T	K	V	G	A	V	N	K	CdeJ
β-OH-Asp(7)	D	L	T	K	V	G	A	V	N	K	CdeJ
Asp(8)	D	L	T	K	V	G	H	I	G	K	(4)**
Asp(9)	D	L	T	K	V	G	H	V	G	K	(7)***
β-OH-Asp(9)	D	L	T	K	V	G	H	V	G	K	(15)****
Asp(10)	D	M	K	D	L	G	M	V	D	K	BrmB, ThaA
β-OH-Asp(10)	D	M	K	D	L	G	M	V	D	K	BrmC, NunE, SyrE, ThaB
β-OH-Asp(11)	D	M	K	E	L	G	M	V	D	K	E7V67_01245, E7V67_22035
AMA(1)	D	P	R	H	L	A	L	L	A	K	Zmn16
Asp(12)	D	P	R	H	V	S	L	L	A	K	FclI
Asp(13)	D	S	W	K	L	G	V	V	D	K	(10)*****
Asn(1)	D	A	T	K	I	G	E	V	G	K	MscF
Asn(2)	D	A	T	K	V	G	E	V	G	K	FclI(2), Zmn16(2), PabF
β-OH-Asn(2)	D	A	T	K	V	G	E	V	G	K	HynA(2), LybB
Asn(3)	D	A	V	Q	M	G	C	V	D	K	LocB
β-OH-Asn(4)	D	F	T	K	I	G	E	V	G	K	BksG, OcfD
Asn(5)	D	G	T	K	L	G	E	V	G	K	WAPS1, WAPS2
Asn(6)	D	G	T	K	V	G	E	V	A	K	DkxJ
Asn(7)	D	L	T	K	I	G	E	V	G	K	(26)[#]
β-OH-Asn(7)	D	L	T	K	I	G	E	V	G	K	CtmC
Cya-3(1)	D	L	T	K	I	G	E	V	G	K	AlbIV
Asn(8)	D	L	T	K	M	G	E	V	G	K	(4)[##]
Asn(9)	D	L	T	K	V	G	E	V	G	K	(4)[###]
Asn(10)	D	L	T	K	V	G	H	V	G	K	GlpE
Asn(11)	D	M	T	K	V	G	E	V	G	K	CtmA
Asn(12)	D	M	T	T	L	G	E	V	G	K	BlmVI

*: AvCA_255708, PfbJ, PblM, PSPPH_1924, PSPTO_2148, PSPPH_1926, PSPTO_2150, PputGB1_4085, PP_4220, PvdJ(2)/BG51_RS09200;

**: BacC, SubC, LicB, SrfA-B;

***: AvCA_25580, CucF, PhmA, PputGB1_4086, PP_4221, PvdI/PFL_4095, TaiF;

****: CroG, Daci_4754, GlmH, GrbI, CUJ87_10420, MbaI, OrbI, PfbI, PvdK/Pfl01_1847, Hsero_2343(2), TaiE, Vapar_3742, Var5, VarH;

*****: AniA/ClpA, AniC/ClpC, ArfA, ArfC, Ban(BW11P2)A, Ban(COW3)A, GamA, LokC, LokA, SteA;

[#]: AmiI-1, BmyD-A, BmyD-B, BmyL-A, BmyL-B, Bacilosarcin synthetase 1, BacC, SubC, BreC, CysH, FusA, EdeP, ItuA-A, ItuA-B, ItuA-C, BacA(2), LatD, MycA, MycB, MycC, ERIC2_c18770, ERIC2_c18760, ERIC2_c18750, TycC5, ZmaO;

[##]: AltL/PPIS_a0416, AltL/CKO50_23365, AltM/PPIS_a0414, AltM/CKO50_23030;

[###]: AmiA, BlmX, ClbN, ThxC2

Table S16 SNSs located Thr-/Dhb-activating domains and their NRPS/NRPS-PKS subunits

Substrates (Serial number)	Residues at positions within the GrsA Phe-activating domain 235	236	239	278	299	301	322	330	331	517	Subunit Name (Frequency)
Thr(1)	D	F	W	N	I	G	M	V	H	K	(113)*
N-Me-Thr(1)	D	F	W	N	I	G	M	V	H	K	(6)**
N,*O*-diMe-Thr(1)	D	F	W	N	I	G	M	V	H	K	FrsG, YtfG
Dhb(1)	D	F	W	N	I	G	M	V	H	K	(40)***
Thr(2)	D	F	W	N	V	G	M	V	H	K	(25)****
Dhb(2)	D	F	W	N	V	G	M	V	H	K	(9)*****
Thr(3)	D	F	W	S	I	G	M	V	H	K	JagA, JagB
Dhb(3)	D	F	W	S	I	G	M	V	H	K	JagA, SypA-PsyrH_12985
Thr(3)	D	F	W	S	V	G	M	V	H	K	JagD
γ-Cl-Thr(3)	D	F	W	S	V	G	M	V	H	K	BrmA, NunB1, SyrB1, ThaC1
Dhb(3)	D	F	W	S	V	G	M	V	H	K	JagC
Thr(5)	D	M	F	C	A	G	L	I	W	K	PmsG
Thr(6)	D	M	F	S	A	G	L	I	W	K	VibF
Thr(7)	D	M	F	V	A	G	L	I	W	K	BasA
β-OH-Thr/Thr(8)	D	M	F	N	F	G	V	L	W	K	LeuA
Ser(16)/Thr(9)	D	M	F	C	N	G	I	I	W	K	FbsF
Thr(10)	D	F	F	N	I	G	T	V	F	K	MynB
Dhb(5)	D	F	W	S	F	G	L	V	H	K	DepE
Thr(11)	D	F	Y	W	M	G	V	L	W	K	DkxG

*: AltK(2), AniB/ClpB, ArfB, DhbF(3), BmyD-C, BmyL-C, Ban(BW11P2)B, Ban(COW3)B, BraB, BrmC(2), BrpC, BreC, Bkm2, Bkm3(2), CipF, PE-PmxE, PE-PmxB, CrpC, CpnD, Daci_4754, DidD, EpyD-EpyE, FclJ, PpsB, FusA(2), GlbF, GlmH, GrbI(2), CUJ87_10420(2), HepA/RzmA, SidK, SidP, BacC, JesA, JesC(2), Plu2670/KolA(2), KorA, KrsA, LatC, LocA, LokB, Plu1878, MynD, MassB, MegI, EA58_10630, NunE(2), NupC, OfaB, PbtA, PoaB, PA-PmxE, PA-PmxA, PA-PmxB, PB-PmxE, PB-PmxB, PD-PmxE, PD-PmxA, PD-PmxB, PP-PmxE, PP-PmxA, PP-PmxB, Zmn17, PekB, PdmB, PSPPH_1924, PSPTO_2148, PSPPH_1925, PSPTO_2149, PvdJ(2)/PT02606, PvdD/PA2399(2), Pfl01_1849, RmyA, BSL056_RS13665, Hsero_2343, SesC, SteB, SyfB/Pspto_2833, SypC-Psyr_2616, SypC-PsyrB_13265, SypC-PsyrH_12980, Xsze_03460, TaiF, TxlA, ThaB, TnpC, ThxA2, TaaE, Vapar_3743, VioC, ViscC, VsmB, WlpB, XNC1-2713, Xekj_RS17945, XabAB, Xekj_00203(3), Xekk_02473, YtfD;

**: MchA-B, MchB-B, MchC-B, MchD1-B, MchD2-B, MchS-B;

***: BogA, BrpA(2), BrvA, BtA, BgdA, BgddA, CipA, CipB-CipC, CipF, CrpA(2), CrpC, Daci_4753, HrmC, HptC(2), NupA(2), NupC, SesA, SesC, SypA-Psyr_2614, SypB-Psyr_2615, SypC-Psyr_2616, SypC-Psyr_2616, SypA-PsyrB_13255, SypB-PsyrB_13260, SypC-PsyrB_13265(2), SypA-PsyrH_12990, SypB-PsyrH_12985, SypC-PsyrH_12980, ThaB, TnpA(2), TnpC, TaaA, TaaE, VioC;

****: BrpC, CdeI, FenB, Fr9D, Fr9DEF, LybB, LesA, LesB, EA58_10630, PaeF(3), PttC, PvdJ(II), PputGB1_4083, PvdJ/PFL_4094, APS14_17625(2), DCC84_23180(2), RHA1_ro02319, SwrA, Txo2, TstDEF, ThfB;

*****: BptE, GlpD, GlpE, HgdA(2), HgddC(2), PppA, SyrE

Table S17 SNSs located Ser-/Dha and Hse-/Hsl-activating domains and their NRPS/NRPS-PKS subunits

Substrates (Serial number)	Residues at positions within the GrsA Phe-activating domain										Subunit Name (Frequency)
	235	236	239	278	299	301	322	330	331	517	
Ser(1)	D	V	W	H	F	S	L	I	D	K	(15)*
Ser(2)	D	V	W	H	F	S	L	V	D	K	(45)**
Ox-Ser(2)	D	V	W	H	F	S	L	V	D	K	DszC(2)
Ser(3)	D	V	W	H	I	S	L	I	D	K	(9)***
Ser(4)	D	V	W	H	I	S	L	V	D	K	GlpC, GlpE
Ser(5)	D	V	W	H	L	S	L	I	D	K	(12)****
Ser(6)	D	V	W	H	L	S	L	V	D	K	(9)*****
N-Me-Dha(1)	D	V	W	H	L	S	L	V	D	K	YtfE
Ser(7)	D	V	W	H	M	S	L	I	D	K	EA58_10630
Ser(8)	D	V	W	H	M	S	L	V	D	K	(43)#
Dha(2)	D	V	W	H	M	S	L	V	D	K	(5)##
N-Me-Dha(2)	D	V	W	H	M	S	L	V	D	K	FrsE
Ser(8)	D	V	W	H	V	S	L	I	D	K	(35)###
Ser(9)	D	V	W	H	V	S	L	V	D	K	(10)####
N-Me-Ser(10)	D	V	W	H	V	S	L	V	D	K	MXAN_3779
Ser(11)/Ala(8)	D	I	N	Q	L	S	M	I	W	K	MegI
Ser(12)	D	I	W	H	I	S	L	I	E	K	Daci_4756, PfbG
Ser(13)	D	L	W	H	L	S	L	I	D	K	SyrE
Ser(14)	D	L	W	N	I	A	S	I	W	K	PynH
Ser(15)/Ala(13)	D	L	W	N	M	G	E	V	W	K	Arg3
Ser(16)/Thr(9)	D	M	F	C	N	G	I	I	W	K	FbsF
Hse(1)	D	L	K	N	V	G	S	D	V	K	(10)#####
Hse(2)	D	L	K	N	L	G	T	D	V	K	AvCA_25580, AvCA_25570
Hsl(1)	D	L	K	N	L	G	T	D	V	K	AvCA_25560

*: AlmB, Alb5, AmbS, BmyD-C, BmyL-C, ClbH, CroD, CroF, HcsG(2), ItuA-C, BacC, MycC, PyxE, ThxA2;

**: AebF(4), AzeB, BmyL-B, BreD, ChiD, EntF(3), FreF(3), HrmC, BacB, KrsC, LatC, PbtA, PbtB, PamE, PhnC, PlpF, PD-PmxE, PxaA, RizD, RhiB, RzxB, BrL25_22185, TtcB(3), TtmA(2), TttA(2), CbsF(3), TriD(2), TnbF(3), VabF;

***: AbsE, BlmVI, GlmI, IcoS(2), PxnG, PvdJ(2)/PT02606, Hsero_2343(2);

****: BraB, BrmB, Daci_4753, NunD, NupA, PfbJ, RmyA, SyrE, SypC-Psyr_26166, SypC-PsyrB_13265, SypC-PsyrH_12980, ThaA;

*****: EtlB, EtlC, PsoB(2), PsoC, WbpA, WlpB, WlpC, XtlB;

#: ClpB/AniB, ClpC/AniC, ArfB, ArfC, Ban(COW3)B, BolH, BrpB, BrpC, Bkm1(2), CipF, CrpC, E7V67_01245, E7V67_01250, GamB, GamC, GcnH, JesB, JesC, LokB, LokA, LybB, MassB, MassC, NupC, OfaB, OfaC, PoaB, PoaC, PekB, PdmB, PdmC, PSPTO_2149, SwrA, SesA, SteB, TnpC, TaaB, ViscC, ViscB, VsmB, VsmC, VifB;

##: Arg2, CrpB, NupB, RmyB, TnpB;

###: AvCA_25580, AvCA_25570, BolH, EpyE, GcnH, MbaI, OrbI, PhmA, PblM, PblL, PSPPH_1925, PSPPH_19260, PSPTO_2150, PvdI(III), PvdI(II), PvdI/PT02609, PputGB1_4085, PP_4219, PvdI/PA2402, PvdI/Pfl01_1845, PvdJ/Pfl01_1846(2), PvdK(1)/BG51_RS09165, APS14_17620, PFLU2543/PvdI, PFLU2544/PvdJ, DCC84_23185, DCC84_23175, TaiG, Vapar_3744, Var6, VarI, WAPS1(2);

####: BksC, BksG, OcfH, OcfD, BptE, LesA, MXAN_3779(2), Txo1, Txo2;

#####: BolH, BrmB, BrpA, CrpA, GcnH, NunD, RmyA, SesC, TnpA, TaaE

Table S18 SNSs located Tyr-activating domains and their NRPS/NRPS-PKS subunits

Substrates	Residues at positions within the GrsA Phe-activating domain										Subunit Name
(Serial number)	235	236	239	278	299	301	322	330	331	517	(Frequency)
Tyr(1)	D	A	A	A	I	A	A	I	C	K	BreC, LatC
Tyr(2)	D	A	A	A	V	V	G	V	C	K	BogD, BrvD, BtE
Tyr(3)	D	A	A	S	I	M	A	V	C	K	ThxA2
N-Me-Tyr(4)	D	A	A	T	V	A	A	V	C	K	CpnD
Tyr(5)	D	A	E	G	I	G	A	V	M	K	(11)*
Tyr(6)	D	A	I	D	V	G	A	V	G	K	TtmB, TttB
Tyr(7)	D	A	L	A	V	G	E	V	V	K	ERIC2_c18760
Tyr(8)	D	A	L	S	V	G	E	V	V	K	(5)**
Tyr(9)	D	A	L	T	T	G	E	V	V	K	TycC
Tyr(10)	D	A	M	S	I	G	A	V	I	K	AusA
Tyr(11)	D	A	P	F	C	G	G	T	C	K	(3)***
ε-OH-Tyr(12)	D	A	P	T	L	G	A	V	N	K	FbnH
Tyr(13)	D	A	S	C	I	A	A	V	C	K	Xsze_03460
Tyr(14)	D	A	S	F	I	A	D	V	C	K	Plu2670/KolA,PdtS(2)
Tyr(15)	D	A	S	T	A	V	G	V	C	K	HgddC
Tyr(16)	D	A	S	T	I	A	A	V	C	K	(10)****
β-OH-Tyr(16)	D	A	S	T	I	A	A	V	C	K	RmyA
Tyr(17)	D	A	S	T	L	A	A	V	C	K	JagB
N-Me-Tyr(17)	D	A	S	T	L	A	A	V	C	K	DidJ
Tyr(18)	D	A	S	T	L	A	G	V	C	K	FusA
Tyr(19)	D	A	S	T	T	A	G	V	C	K	HgdA
β-Cl-Tyr(20)	D	A	S	T	V	A	A	I	G	K	CndF
Tyr(21)	D	A	S	T	V	A	A	V	C	K	(9)*****
Tyr(22)	D	G	A	C	V	A	L	I	L	K	NcyF
Tyr(23)	D	G	E	D	V	A	A	V	C	K	MXAN_3779
Tyr(24)	D	G	L	F	T	V	R	V	E	K	LocC
β-OH-Tyr/β-Cl-β-OH-Tyr(25)	D	G	M	N	I	A	S	V	C	K	BksE, OcfF
Tyr(26)	D	G	S	T	I	T	A	V	A	K	CmdD
Tyr(27)	D	G	T	I	T	A	E	V	A	K	FenD, PpsB, PpsD
Tyr(28)	D	G	T	L	T	A	E	V	A	K	FenB
Tyr29)	D	G	V	D	V	G	A	V	S	K	TtcC
Tyr(30)/Phe(28)	D	P	H	M	I	G	C	I	H	K	TtcA
β-OH-δ-Me-*O*-Me-Tyr(31)	D	P	W	G	L	G	L	I	D	K	EtuA2(2), SacC(2), SafA(2)
Tyr(32)	D	T	S	T	L	A	A	V	A	K	PhnE
Tyr(33)	D	V	K	V	F	G	A	V	Q	K	BrL25_22185
Tyr(34)/Trp(23)/Phe(35)	D	V	S	A	I	G	C	V	T	K	LgrC
Tyr(35)	D	V	W	H	F	G	R	I	N	K	PvdL/PT01550

*: AvCA_25650, PSPPH_1911, PSPTO_2135, PputGB1_3809, PP_4243, PvdL/PA2424, PvdL/Pfl01_3940, PvdL/ BG51_RS0121050, PvdL/PFL_4189, PvdL/APS14_09630, PFLU6137;

**: BmyD-B, BmyL-B, ItuA-B, BacB, MycB;

***: SypC-Psyr_2616, SypC-PsyrB_13265, SypC-PsyrH_12980

****: BrnE, EpyE(2), HepA/RzmA, HynA, BSL056_RS13665, BSL056_RS13660, ThxA2, ThxC2, ThxC2;

*****: CdeI, CtmD, XPG1_1613, XPG1_1612, XDD1_1906-XDD1_1907, XDD1_1907, XDD1_1906(2), PppA

Table S19 SNSs located Phe-activating domains and their NRPS/NRPS-PKS subunits

Substrates (Serial number)	Residues at positions within the GrsA Phe-activating domain										Subunit Name (Frequency)
	235	236	239	278	299	301	322	330	331	517	
Phe((1)	D	A	A	T	A	G	L	I	M	K	AntB
Phe(2)	D	A	F	T	I	A	A	V	C	K	HolA
Phe(3)	D	A	F	T	V	A	A	I	W	K	BgdB, BgddA, BptE, LybA
Phe(4)	D	A	F	T	V	A	A	V	C	K	BacC, SubC, CpnD, Xekk_02473
N-Me-Phe(5)	D	A	F	T	V	A	A	V	W	K	Txo11
N-Me-Phe(6)	D	A	F	T	V	A	G	V	C	K	XndB
Phe(7)	D	A	L	I	V	A	A	V	C	K	CtmC
Phe(8)/Trp(15)	D	A	L	V	M	G	A	V	M	K	TycB
Phe(9)	D	A	M	F	I	C	G	I	C	K	SAMN04488128_1011792
Phe(10)/Trp(16)	D	A	M	I	G	G	M	V	T	K	AmoG
Phe(11)	D	A	P	I	M	G	G	T	C	K	JesC, SyrE
Phe(12)/Trp(18)	D	A	W	A	F	A	G	V	A	K	PhnE, TriD
Phe(13)/Leu(24)	D	A	W	C	I	A	A	V	C	K	Plu3263/GxpS
Phe(13)	D	A	W	C	I	A	A	V	C	K	Xsze_03460
Phe(14)	D	A	W	I	I	G	A	I	V	K	OctB
β-OH-*p*-NO_2-homoPhe(15)	D	A	W	G	C	G	L	I	N	K	ObiF
Phe(16)	D	A	W	Q	L	G	L	N	D	K	XPG1_1613, XDD1_1908
Phe(17)	D	A	W	T	F	A	A	I	A	K	PhnE
Phe(18)	D	A	W	T	F	A	G	V	A	K	TriD
Phe(19)	D	A	W	T	I	A	A	I	A	K	PB-PmxA, PP-PmxA
Phe(20)	D	A	W	T	I	A	A	I	C	K	GrsA(2), PlpE, SevA, TycA
N-Me-Phe(21)	D	A	W	T	I	A	A	V	C	K	LesA, WAPS, WbpA
Phe(22)	D	A	W	T	I	A	G	V	C	K	TycB
Phe(23)	D	A	W	T	V	A	A	V	C	K	PttB
Phe(24)	D	A	W	Y	N	G	N	V	L	K	P615_17225
Phe(25)	D	A	Y	V	G	A	A	V	W	K	KorB
Phe(26)/Trp(4)	D	G	F	A	V	A	A	V	G	K	XNC1_2713(2)
Phe(27)	D	M	V	L	Q	G	G	A	C	K	BN712_00198
Phe(28)/Tyr(30)	D	P	H	M	I	G	C	I	H	K	TtcA
Phe(29)	D	P	L	V	I	G	G	T	A	K	SwrA
Phe(30)	D	S	I	I	G	A	G	V	C	K	KorC
Phe(31)	D	T	W	T	I	A	S	V	S	K	Zmn16
Phe(32)	D	T	W	T	L	A	S	V	G	K	FclI
Phe(33)	D	V	F	T	Y	A	L	V	Y	K	BlmIX
Phe(34)	D	V	S	A	C	G	C	I	I	K	AmbS
Phe(35)/Trp(23)/Tyr(34)	D	V	S	A	I	G	C	V	T	K	LgrC
Phe(36)	D	V	S	T	C	G	C	I	I	K	AmbS

Table S20 SNSs located Trp-activating domains and their NRPS/NRPS-PKS subunits

Substrates (Serial number)	Residues at positions within the GrsA Phe-activating domain										Subunit Name (Frequency)
	235	236	239	278	299	301	322	330	331	517	
Trp(1)	D	A	A	I	I	G	E	I	W	K	AqmG
Trp(2)	D	A	A	I	I	G	E	V	W	K	LesB
Trp(3)	D	A	A	I	V	G	E	I	W	K	WbpB
Trp(4)/Phe(26)	D	G	F	A	V	A	A	V	G	K	XNC1_2713(2)
Trp(4)	D	G	F	A	V	A	A	V	G	K	XPG1_1612
Trp(5)	D	G	F	A	V	A	S	V	C	K	XPG1_1613, XDD1_1906
Trp(6)	D	G	W	A	L	A	A	V	T	K	Arg3
Trp(7)	D	G	W	A	T	A	V	V	T	K	Arg3
Trp(8)	D	P	T	Q	A	G	E	V	V	K	BreC
Trp(9)	D	P	T	Q	V	G	E	V	V	K	BreC, LatC(2)
Trp(10)	D	V	S	S	E	G	C	V	G	K	LgrD
Trp(11)	D	V	S	S	I	G	C	V	C	K	LgrC, LgrD
Trp(12)	D	A	G	L	V	G	E	V	W	K	WAPS2
Trp(13)	D	A	K	A	F	G	A	I	Q	K	BrL25_22185
Trp(14)	D	A	L	A	V	G	Q	V	V	K	SevB
Trp(15)/Phe(8)	D	A	L	V	M	G	A	V	M	K	TycB
Trp(16)Phe(10)	D	A	M	I	G	G	M	V	T	K	AmoG
Trp(17)	D	A	S	L	V	G	D	V	C	K	PdtS
Trp(18)/Phe(12)	D	A	W	A	F	A	G	V	A	K	TriD
N-Me-Trp(19)	D	G	V	Q	M	A	G	V	G	K	CmdD
Trp(20)	D	P	F	S	I	G	M	I	I	K	LugA
Trp(21)	D	V	Q	C	I	G	D	V	C	K	PdtS
Trp(22)	D	V	S	A	C	G	C	I	T	K	Plu3123
Trp(23)/Phe(35)/Tyr(34)	D	V	S	A	I	G	C	V	T	K	LgrC
Trp(24)	D	V	S	Q	V	G	A	V	A	K	MscH
Trp(25)	D	V	W	S	I	G	V	I	C	K	Xsze_03460

Table S21 SNSs located Dab-, Dap- and His-activating domains and their NRPS/NRPS-PKS subunits

Substrates	Residues at positions within the GrsA Phe-activating domain										Subunit Name
(Serial number)	235	236	239	278	299	301	322	330	331	517	(Frequency)
Dab(1)	D	G	E	D	Q	T	T	V	T	K	CroG
Dab(2)	D	I	W	E	L	T	A	D	D	K	(20)*
Dab(3)	D	I	W	E	L	T	V	D	D	K	GlmI
Dab(4)	D	L	E	H	N	T	T	I	S	K	Bkm2
Dab(5)	D	L	E	H	N	T	T	V	S	K	(20)**
Dab(6)	D	V	C	E	T	G	T	I	E	K	PhnD
Dab(7)	D	V	G	E	I	S	A	I	D	K	(28)***
Dab(8)	D	V	G	E	I	S	S	I	D	K	(11)****
Dab(9)	D	V	G	E	L	S	S	I	D	K	PE-PmxE
β-OH-Dab(10)	D	V	W	Q	M	I	G	D	D	K	Odl1(2)
Dap(1)	D	I	W	E	A	T	M	D	D	K	EdeK
N-Me-Dap(2)	D	I	W	E	V	T	A	D	D	K	PamC, PamH
Dap(3)	D	V	W	E	M	N	A	D	D	K	SulI
His(1)	D	S	A	L	I	A	E	V	W	K	(7)*****
β-OH-His(1)	D	S	A	L	I	A	E	V	W	K	GlmG, HcsF, HcsI
His(2)	D	S	E	L	T	A	E	V	C	K	BacC, SubC
His(3)	D	S	P	L	I	A	E	V	W	K	Odl3
His(4)	D	S	Q	F	I	G	Q	V	V	K	KrsC

*: AvCA_25650, BksF, OcfE, CucF, GlmG, HcsG, HcsH, PSPPH_1911, PSPTO_2135, PvdI(III), PvdL/PT015503, PputGB1_3809, PP_4243, PP_4220, PvdL/PA24243, PvdL/Pfl01_3940, PvdD/BG51_RS09160, PvdL/PFL_4189, PvdL/APS14_09630, Pflu6137;

**: BrpC, CipF, CrpC, GlpC, GlpD, JesC, NunD(2), NupC, SesC, SyrE(2), SypC-Psyr_2616(2), SypC-PsyrB_13265(2), SypC-PsyrH_12980(2), TnpC, TaaE;

***: PE-PmxE(2), PE-PmxA(2), OctA(3), OctB(2), PlpD, PlpE(2), PA-PmxE(2), PA-PmxA(2), PB-PmxE(2), PB-PmxA(2), PD-PmxE(2), PD-PmxA(2), PP-PmxE(2), PP-PmxA(2);

****: PE-PmxE, PA-PmxE(2), PB-PmxE(2), PD-PmxE, PP-PmxE(2), Tri-D(3);

*****: BlmX, BrmB, JagD, MynF, SAMN04488128_1011792, PvdJ(1)/PT02607, ThaA

Table S22 Bacterial species known to synthesize NRPs through NRPS services, documented from the year 2023 to 2024, alongside their genomic information

No.	Bacterial sources	Genome ID (GenBank)	NRPS services	Known Product Name	Ref.
1	*B. amyloliquefaciens* MR14M3	OQ118400 (16S rRNA)	AntiSMASH 6.1.1, MIBiG Version 3.1	Locillomycin, fengycin/plipastatin, surfactin, bacillaene, bacillomycin D, and mycosubtilin	301
2	*B. velezensis* NDB	CP135184	AntiSMASH and BAGEL4	Fengycin, bacillomycin, bacillaene	302
3	*Bacillus halotolerans* AQ11M9	MZ619061 (16S rRNA)	AntiSMASH 6.1.1	Bacillibactin, paenibactin, surfactin and plipastin/fengycin	303
4	*Bacillus paralicheniformis* PBl 36	JAPEZR000000000	AntiSMASH 4.1.0	Bacillibactin, lichenysin, fengycin, bacitracin	304
5	*B. subtilis* BS21	None	AntiSMASH	Bacillibactin, fengycin, surfactin and zwittermicin A	305
6	*B. subtilis* PBs 12	CP110213	AntiSMASH 4.1.0	Thailanstatin A, fengycin and surfactin	304
7	Thirteen isolates of *Xenorhabdus* and *Photorhabdus*	From SAMN36278238 to SAMN36278250	AntiSMASH 4.0.2	Photoxenobactin, tilivalline, odilorhabdin, GXP, ririwpeptide, andrimid, xenoamicin, et al	306

Table S23 Subunits in an iterative manner

No.	Product Name	Subunits	No.	Product Name	Subunits
14	Amphi-enterobactin	AebF	190	Paenibactin/Paebacillibactin	PaeF
29	Bacillibactin	DhbF	204	Photoxenobactins	PxbF, PxbG
63	Cereulide	CesA, CesB	206	Piscibactin	Irp2
83	Didemnins	DidA	247	Rhabdopeptides	RdpB, RdpC
90	Enterobactin	EntF	251	Rhodochelin	RHA1_ro02319
93	Equibactin	EqbE	277	Thalassospiramides B-like, C-like and F	TtcB
99	FK228/FR901228	DepD	278–279	Thalassospiramides A4 and E	TtmA/TttA
104	Frederiksenibactin	FreF	285	Thiomarinol BGC	HolA
114	Gramicidin S	GrsA, GrsB	290	Trichrysobactin	CbsF
137	Koranimine	KorA, KorD	293	Turnerbactin	TnbF
146	Locillomycin	LocB	301	Vicibactin	VbsS
148	Lugdunin	LugC	320	Yersiniabactin	HMWP2
160	Micacocidin	MicC			

References

1. Mihara K, Tanabe T, Yamakawa Y, Funahashi T, Nakao H, Narimatsu S, Yamamoto S (2004) Identification and transcriptional organization of a gene cluster involved in biosynthesis and transport of acinetobactin, a siderophore produced by *Acinetobacter baumannii* ATCC 19606^{T}. Microbiology 150:2587–2597. https://doi.org/10.1099/mic.0.27141-0

2. Ye L, Cornelis P, Guillemyn K, Ballet S, Hammerich O (2014) Structure revision of *N*-mercapto-4-formylcarbostyril produced by *Pseudomonas fluorescens* G308 to 2-(2-hydroxyphenyl)thiazole-4-carbaldehyde [aeruginaldehyde]. Nat Prod Commun 9(6):789–794. https://doi.org/10.1177/1934578X1400900615

3. Buntin K, Rachid S, Scharfe M, Blöcker H, Weissman KJ, Müller R (2008) Production of the antifungal isochromanone ajudazols A and B in *Chondromyces crocatus* Cm c5: biosynthetic machinery and cytochrome P450 modifications. Angew Chem Int Ed Engl 47(24):4595–4599. https://doi.org/10.1002/anie.200705569

4. Patteson JB, Dunn ZD, Li B (2018) In Vitro Biosynthesis of the nonproteinogenic amino acid Methoxyvinylglycine. Angew Chem Int Ed Engl 57(23):6780–6785. https://doi.org/10.1002/anie.201713419

5. Hashimi SM (2019) Albicidin, a potent DNA gyrase inhibitor with clinical potential. J Antibiot 72:785–792. https://doi.org/10.1038/s41429-019-0228-2

6. Ross AC, Gulland LE, Dorrestein PC, Moore BS (2015) Targeted capture and heterologous expression of the *Pseudoalteromonas* alterochromide gene cluster in *Escherichia coli* represents a promising natural product exploratory platform. ACS Synth Biol 4(4):414–420. https://doi.org/10.1021/sb500280q

7. Chau R, Pearson LA, Cain J, Kalaitzis JA, Neilan BA (2021) A *Pseudoalteromonas* clade with

remarkable biosynthetic potential. Appl Environ Microbiol 87:e02604-20. https://doi.org/10.1128/AEM.02604-20

8. Cortina NS, Revermann O, Krug D, Müller R (2011) Identification and characterization of the althiomycin biosynthetic gene cluster in *Myxococcus xanthus* DK897. Chembiochem 12(9):1411–1416. https://doi.org/10.1002/cbic.201100154.

9. Gerc AJ, Song L, Challis GL, Stanley-Wall NR, Coulthurst SJ (2012) The insect pathogen *Serratia marcescens* Db10 uses a hybrid non-ribosomal peptide synthetase-polyketide synthase to produce the antibiotic althiomycin. PLoS One 7(9):e44673. https://doi.org/10.1371/journal.pone.0044673

10. Tobias NJ, Wolff H, Djahanschiri B, Grundmann F, Kronenwerth M, Shi YM, Simonyi S, Grün P, Shapiro-Ilan D, Pidot SJ, Stinear TP, Ebersberger I, Bode HB (2017) Natural product diversity associated with the nematode symbionts *Photorhabdus* and *Xenorhabdus*. Nat Microbiol 2(12):1676–1685. https://doi.org/10.1038/s41564-017-0039-9

11. Park HB, Perez CE, Perry EK, Crawford JM (2016) Activating and attenuating the amicoumacin antibiotics. Molecules 21(7):824. https://doi.org/10.3390/molecules21070824

12. Esmaeel Q, Chevalier M, Chataigné G, Subashkumar R, Jacques P, Leclère V (2016) Nonribosomal peptide synthetase with a unique iterative-alternative-optional mechanism catalyzes amonabactin synthesis in *Aeromonas*. Appl Microbiol Biotechnol 100(19):8453–8463. https://doi.org/10.1007/s00253-016-7773-4

13. Galvis F, Ageitos L, Martínez-Matamoros D, Barja JL, Rodríguez J, Lemos ML, Jiménez C, Balado M (2020) The marine bivalve molluscs pathogen *Vibrio neptunius* produces the siderophore amphibactin, which is widespread in molluscs microbiota. Environ Microbiol 22(12):5467–5482. https://doi.org/ 10.1111/1462-2920.15312

14. Zane HK, Naka H, Rosconi F, Sandy M, Haygood MG, Butler A (2014) Biosynthesis of

amphi-enterobactin siderophores by *Vibrio harveyi* BAA-1116: identification of a bifunctional nonribosomal peptide synthetase condensation domain. J Am Chem Soc 136:5615–5618. https://doi.org/10.1021/ja5019942

15. Jin M, Fischbach MA, Clardy J (2006) A biosynthetic gene cluster for the acetyl-CoA carboxylase inhibitor andrimid. J Am Chem Soc 128(33):10660–106601. https://doi.org/ 10.1021/ja063194c
16. Magarvey NA, Fortin PD, Thomas PM, Kelleher NL, Walsh CT (2008) Gatekeeping versus promiscuity in the early stages of the andrimid biosynthetic assembly line. ACS Chem Biol 3(9):542–54. https://doi.org/10.1021/cb800085g
17. Naka H, Liu M, Actis LA, Crosa JH (2013) Plasmid- and chromosome-encoded siderophore anguibactin systems found in marine vibrios: biosynthesis, transport and evolution. Biometals 26(4):537–547. https://doi.org/10.1007/s10534-013-9629-z.
18. Götze S, Herbst-Irmer R, Klapper M, Görls H, Schneider KRA, Barnett R, Burks T, Neu U, Stallforth P (2017) Structure, biosynthesis, and biological activity of the cyclic lipopeptide anikasin. ACS Chem Biol 12(10):2498–2502. https://doi.org/10.1021/acschembio.7b00589
19. Tautz T, Hoffmann J, Hoffmann T, Steinmetz H, Washausen P, Kunze B, Huch V, Kitsche A, Reichenbach H, Höfle G, Müller R, Kalesse M (2016) Isolation, structure elucidation, biosynthesis, and synthesis of antalid, a secondary metabolite from *Polyangium* species. Org Lett 18(11):2560–2563. https://doi.org/10.1021/acs.orglett.6b00810
20. Dieterich CL, Probst SI, Ueoka R, Sandu I, Schäfle D, Molin MD, Minas HA, Costa R, Oxenius A, Sander P, Piel J (2022) Aquimarins, peptide antibiotics with amino-modified C-termini from a sponge-derived *Aquimarina* sp. bacterium. Angew Chem Int Ed Engl 61(8):e202115802. https://doi.org/10.1002/anie.202115802
21. Pogorevc D, Tang Y, Hoffmann M, Zipf G, Bernauer HS, Popoff A, Steinmetz H, Wenzel SC (2019)

Biosynthesis and heterologous production of argyrins. ACS Synth Biol 8:1121–1133. https://doi.org/10.1021/acssynbio.9b00023

22. Lange A, Sun H, Pilger J, Reinscheid UM, Gross H (2012) Predicting the structure of cyclic lipopeptides by bioinformatics: structure revision of arthrofactin. Chembiochem 13(18):2671–2675. https://doi.org/10.1002/cbic.201200532
23. Roongsawang N, Hase Ki, Haruki M, Imanaka T, Morikawa M, Kanaya S (2003) Cloning and characterization of the gene cluster encoding arthrofactin synthetase from *Pseudomonas* sp. MIS38. Chem Biol 10(9):869–80. https://doi.org/10.1016/j.chembiol.2003.09.004
24. Wilson DJ, Shi C, Teitelbaum AM, Gulick AM, Aldrich CC (2013) Characterization of AusA: a dimodular nonribosomal peptide synthetase responsible for the production of aureusimine pyrazinones. Biochemistry 52(5):926–937. https://doi.org/10.1021/bi301330q
25. Theodore CM, Stamps BW, King JB, Price LS, Powell DR, Stevenson BS, Cichewicz RH (2014) Genomic and metabolomic insights into the natural product biosynthetic diversity of a feral-hog-associated *Brevibacillus laterosporus* strain. PLoS One 9(3):e90124. https://doi.org/10.1371/journal.pone.0090124
26. Hong Z, Bolard A, Giraud C, Prévost S, Genta-Jouve G, Deregnaucourt C, Häussler S, Jeannot K, Li Y (2019) Azetidine-containing alkaloids produced by a quorum-sensing regulated nonribosomal peptide synthetase pathway in *Pseudomonas aeruginosa*. Angew Chem Int Ed Engl 58:3178–3182. https://doi.org/10.1002/anie.201809981
27. Patteson JB, Lescallette AR, Li B (2019) Discovery and biosynthesis of azabicyclene, a conserved nonribosomal peptide in *Pseudomonas aeruginosa*. Org Lett 21(13):4955–4959. https://doi.org/10.1021/acs.orglett.9b01383
28. Baars O, Zhang X, Morel FM, Seyedsayamdost MR (2016) The siderophore metabolome of

Azotobacter vinelandii. Appl Environ Microbiol 82:27–39. https://doi.org/10.1128/AEM.03160-15

29. Yoneyama F, Yamamoto M, Hashimoto W, Murata K (2011) *Azotobacter vinelandii* gene clusters for two types of peptidic and catechol siderophores produced in response to molybdenum. J Appl Microbiol 111(4):932–938. https://doi.org/10.1111/j.1365-2672.2011.05109.x
30. Chen XH, Vater J, Piel J, Franke P, Scholz R, Schneider K, Koumoutsi A, Hitzeroth G, Grammel N (2006) Strittmatter AW, Gottschalk G, Süssmuth RD, Borriss R. Structural and functional characterization of three polyketide synthase gene clusters in *Bacillus amyloliquefaciens* FZB42. J Bacteriol 188(11):4024–4036. https://doi.org/10.1128/JB.00052-06
31. Zhang F, Wang Y, Jiang Q, Chen Q, Karthik L, ZhaoYL, Li Z (2018) Substrate selection of adenylation domains for nonribosomal peptide synthetase (NRPS) in bacillamide C biosynthesis by marine *Bacillus atrophaeus* C89. J Ind Microbiol Biotechnol 45:335–344. https://doi.org/10.1007/s10295- 018-2028-2
32. Guo S, Li X, He P, Ho H, Wu Y, He Y (2015) Whole-genome sequencing of *Bacillus subtilis* XF-1 reveals mechanisms for biological control and multiple beneficial properties in plants. J Ind Microbiol Biotechnol 42(6):925–937. https://doi.org/10.1007/s10295-015-1612-y
33. Zhao P, Quan C, Jin L, Wang L, Guo X, Fan S (2013) Sequence characterization and computational analysis of the non-ribosomal peptide synthetases controlling biosynthesis of lipopeptides, fengycins and bacillomycin D, from *Bacillus amyloliquefaciens* Q-426. Biotechnol Lett 35(12):2155–63. https://doi.org/10.1007/s10529-013-1320-5
34. Luo C, Liu X, Zhou H, Wang X, Chen Z (2015) Nonribosomal peptide synthase gene clusters for lipopeptide biosynthesis in *Bacillus subtilis* 916 and their phenotypic functions. Appl Environ Microbiol 81(1):422–431. https://doi.org/10.1128/AEM.02921-14
35. Shen Q, Zhou H, Dai G, Zhong G, Huo L, Li A, Liu Y, Yang M, Ravichandran V, Zheng Z, Tang YJ,

Jiao N, Zhang Y, Bian X (2022) Characterization of a cryptic NRPS gene cluster in *Bacillus velezensis* FZB42 reveals a discrete oxidase involved in multithiazole biosynthesis. ACS Catal 12(6):3371–3381. https://doi.org/10.1021/acscatal.1c05131

36. Boya CA, Herrera L, Guzman HM, Gutierrez M (2012) Antiplasmodial activity of bacilosarcin A isolated from the octocoral-associated bacterium *Bacillus* sp. collected in Panama. J Pharm Bioallied Sci 4(1):66–69. https://doi.org/10.4103/0975-7406.92739
37. Eppelmann K, Doekel S, Marahiel MA (2001) Engineered biosynthesis of the peptide antibiotic bacitracin in the surrogate host *Bacillus subtilis*. J Biol Chem 276(37):34824–34831. https://doi.org/10.1074/jbc.M104456200
38. Wu S, Zhong J, Huan L(2006) Genetics of subpeptin JM4-A and subpeptin JM4-B production by *Bacillus subtilis* JM4. Biochem Biophys Res Commun 344(4):1147–1154. https://doi.org/10.1016/j.bbrc.2006.04.022
39. Wu S, Jia S, Sun D, Chen M, Chen X, Zhong J, Huan L (2005) Purification and characterization of two novel antimicrobial peptides Subpeptin JM4-A and Subpeptin JM4-B produced by *Bacillus subtilis* JM4. Curr Microbiol 51(5):292–296. https://doi.org/10.1007/s00284-005-0004-3
40. Carr G, Seyedsayamdost MR, Chandler JR, Greenberg EP, Clardy J (2011) Sources of diversity in bactobolin biosynthesis by *Burkholderia thailandensis* E264. Org Lett 13:3048–3051. https://doi.org/10.1021/ol200922s
41. Nguyen DD, Melnik AV, Koyama N, Lu X, Schorn M, Fang J, Aguinaldo K, Lincecum TL Jr, Ghequire MG, Carrion VJ, Cheng TL, Duggan BM, Malone JG, Mauchline TH, Sanchez LM, Kilpatrick AM, Raaijmakers JM, De Mot R, Moore BS, Medema MH, Dorrestein PC (2016) Indexing the *Pseudomonas* specialized metabolome enabled the discovery of poaeamide B and the bananamides. Nat Microbiol 2:16197. https://doi.org/10.1038/nmicrobiol.2016.197

42. Omoboye OO, Geudens N, Duban M, Chevalier M, Flahaut C, Martins JC, Leclère V, Oni FE, Höfte M (2019) *Pseudomonas* sp. COW3 Produces new bananamide-type cyclic lipopeptides with antimicrobial activity against *Pythium myriotylum* and *Pyricularia oryzae*. Molecules 24(22):4170. https://doi.org/10.3390/molecules24224170

43. Rischer M, Raguž L, Guo H, Keiff F, Diekert G, Goris T, Beemelmanns C (2018) Biosynthesis, synthesis, and activities of barnesin A, a NRPS-PKS hybrid produced by an anaerobic Epsilonproteobacterium. ACS Chem Biol 13(8):1990–1995. https://doi.org/10.1021/acschembio.8b00445

44. Wenzel SC, Hoffmann H, Zhang J, Debussche L, Haag-Richter S, Kurz M, Nardi F, Lukat P, Kochems I, Tietgen H, Schummer D, Nicolas JP, Calvet L, Czepczor V, Vrignaud P, Mühlenweg A, Pelzer S, Müller R, Brönstrup M (2015) Production of the bengamide class of marine natural products in Myxobacteria: biosynthesis and structure-activity relationships. Angew Chem Int Ed Engl 54:15560–15564. https://doi.org/10.1002/anie.201508277

45. Fuchs SW, Sachs CC, Kegler C, Nollmann FI, Karas M, Bode HB (2012) Neutral loss fragmentation pattern based screening for arginine-rich natural products in *Xenorhabdus* and *Photorhabdus*. Anal Chem 84:6948–6955. https://doi.org/10.1021/ac300372p

46. Mizuno CM, Kimes NE, López-Pérez M, Ausó E, Rodriguez-Valera F, Ghai R (2013) A hybrid NRPS-PKS gene cluster related to the bleomycin family of antitumor antibiotics in Alteromonas macleodii strains. PLoS One 8(9):e76021. https://doi.org/10.1371/journal.pone.0076021

47. Barsby T, Warabi K, Sørensen D, Zimmerman WT, Kelly MT, Andersen RJ (2006) The Bogorol family of antibiotics: template-based structure elucidation and a new approach to positioning enantiomeric pairs of amino acids. J Org Chem 71(16):6031–6037. https://doi.org/10.1021/jo060667p

48. Dose B, Ross C, Niehs SP, Scherlach K, Bauer JP, Hertweck C (2020) Food-poisoning bacteria

employ a citrate synthase and a type II NRPS to synthesize bolaamphiphilic lipopeptide antibiotics. Angew Chem Int Ed Engl 59(48):21535–21540. https://doi.org/10.1002/anie.202009107

49. Li JH, Cho W, Hamchand R, Oh J, Crawford JM (2021) A conserved nonribosomal peptide synthetase in *Xenorhabdus bovienii* produces citrulline-functionalized lipopeptides. J Nat Prod 84(10):2692–2699. https://doi.org/10.1021/acs.jnatprod.1c00573

50. Schmidt Y, van der Voort M, Crüsemann M, Piel J, Josten M, Sahl HG, Miess H, Raaijmakers JM, Gross H (2014) Biosynthetic origin of the antibiotic cyclocarbamate brabantamide A (SB-253514) in plant-associated *Pseudomonas*. Chembiochem 15(2):259–266. https://doi.org/10.1002/cbic.201300527

51. Zhao H, Liu YP, Zhang LQ (2019) In silico and genetic analyses of cyclic lipopeptide synthetic gene clusters in *Pseudomonas* sp. 11K1. Front Microbio 10:544. https://doi.org/10.3389/fmicb.2019. 00544

52. Yang X, Huang E, Yuan C, Zhang L, Yousef AE (2016) Isolation and structural elucidation of Brevibacillin, an antimicrobial lipopeptide from *Brevibacillus laterosporus* that combats drug-resistant gram-positive bacteria. Appl Environ Microbiol 82:2763–2772. https://doi.org/10.1128/AEM.00315-16

53. Al Ayed K, Ballantine RD, Hoekstra M, Bann SJ, Wesseling CMJ, Bakker AT, Zhong Z, Li YX, Brüchle NC, van der Stelt M, Cochrane SA, Martin NI (2022) Synthetic studies with the brevicidine and laterocidine lipopeptide antibiotics including analogues with enhanced properties and in vivo efficacy. Chem Sci 13(12):3563–3570. https://doi.org/10.1039/d2sc00143h

54. Wu X, Ballard J, Jiang YW (2005) Structure and biosynthesis of the BT peptide antibiotic from *Brevibacillus texasporus*. Appl Environ Microbiol 71(12):8519–8530. https://doi.org/10.1128/AEM.71.12.8519-8530.2005

55. Biggins JB, Gleber CD, Brady SF (2011) Acyldepsipeptide HDAC inhibitor production induced in

Burkholderia thailandensis. Org Lett 13(6):1536–1539. https://doi.org/10.1021/ol200225v

56. Wang C, Henkes LM, Doughty LB, He M, Wang D, Meyer-Almes FJ, Cheng YQ (2011) Thailandepsins: bacterial products with potent histone deacetylase inhibitory activities and broad-spectrum antiproliferative activities. J Nat Prod 74:2031–2038. https://doi.org/10.1021/np200324x
57. Lin Z, Falkinham JO 3rd, Tawfik KA, Jeffs P, Bray B, Dubay G, Cox JE, Schmidt EW (2012) Burkholdines from *Burkholderia ambifaria*: antifungal agents and possible virulence factors. J Nat Prod 75(9):1518–1523. https://doi.org/10.1021/np300108u
58. Gu G, Smith L, Liu A, Lu SE (2011) Genetic and biochemical map for the biosynthesis of occidiofungin, an antifungal produced by *Burkholderia contaminans* strain MS14. Appl Environ Microbiol 77(17):6189–6198. https://doi.org/10.1128/AEM.00377-11
59. Esmaeel Q, Pupin M, Kieu NP, Chataigné G, Béchet M, Deravel J, Krier F, Höfte M, Jacques P, Leclère V (2016) *Burkholderia* genome mining for nonribosomal peptide synthetases reveals a great potential for novel siderophores and lipopeptides synthesis. Microbiologyopen 512–526. https://doi.org/ 10.1002/ mbo3.347
60. Thongkongkaew T, Ding W, Bratovanov E, Oueis E, Garcı A-Altares MA, Zaburannyi N, Harmrolfs K, Zhang Y, Scherlach K, Müller R, Hertweck C (2018) Two types of threonine-tagged lipopeptides synergize in host colonization by pathogenic *Burkholderia* species. ACS Chem Biol 13:1370–1379. https://doi.org/10.1021/acschembio.8b00221
61. Chen H, Sun T, Bai X, Yang J, Yan F, Yu L, Tu Q, Li A, Tang Y, Zhang Y, Bian X, Zhou H (2021) Genomics-driven activation of silent biosynthetic gene clusters in *Burkholderia gladioli* by screening recombineering system. Molecules 26(3):700. https://doi.org/10.3390/molecules26030700
62. Yoshimura A, Covington BC, Gallant É, Zhang C, Li A, Seyedsayamdost MR (2020) Unlocking

cryptic metabolites with mass spectrometry-guided transposon mutant selection. ACS Chem Biol 15(10):2766–2774. https://doi.org/10.1021/acschembio.0c00558

63. Wu C, Shang Z, Lemetre C, Ternei MA, Brady SF (2019) Cadasides, calcium-dependent acidic lipopeptides from the soil metagenome that are active against multidrug-resistant bacteria. J Am Chem Soc 141:3910–3919. https://doi.org/10.1021/jacs.8b12087
64. Demirev AV, Lee CH, Jaishy BP, Nam DH, Ryu DD (2006) Substrate specificity of nonribosomal peptide synthetase modules responsible for the biosynthesis of the oligopeptide moiety of cephabacin in *Lysobacter lactamgenus*. FEMS Microbiol Lett 255(1):121–128. https://doi.org/10.1111/j.1574-6968.2005.00067.x
65. Tempelaars MH, Rodrigues S, Abee T (2011) Comparative analysis of antimicrobial activities of valinomycin and cereulide, the *Bacillus cereus* emetic toxin. Appl Environ Microbiol 77(8):2755–2762. https://doi.org/10.1128/AEM.02671-10
66. Perlova O, Gerth K, Kaiser O, Hans A, Müller R (2006) Identification and analysis of the chivosazol biosynthetic gene cluster from the myxobacterial model strain *Sorangium cellulosum* So ce56. J Biotechnol 121:174–191. https://doi.org/10.1016/j.jbiotec.2005.10.011
67. Gorges J, Panter F, Kjaerulff L, Hoffmann T, Kazmaier U, Müller R (2018) Structure, total synthesis, and biosynthesis of chloromyxamides: Myxobacterial tetrapeptides featuring an uncommon 6-chloromethyl-5-methoxypipecolic acid building block. Angew Chem Int Ed Engl 57:14270–14275. https://doi.org/10.1002/anie.201808028
68. Rachid S, Krug D, Kunze B, Kochems I, Scharfe M, Zabriskie TM, Blöcker H, Müller R (2006) Molecular and biochemical studies of chondramide formation-highly cytotoxic natural products from *Chondromyces crocatus* Cm c5. Chem Biol 13(6):667–681. https://doi.org/10.1016/j.chembiol.2006.06.002

69. Rachid S, Scharfe M, Blöcker H, Weissman KJ, Müller R (2009) Unusual chemistry in the biosynthesis of the antibiotic chondrochlorens. Chem Biol 16:70–81. https://doi.org/10.1016/j. chembiol.2008. 11.005

70. Pauwelyn E, Huang CJ, Ongena M, Leclère V, Jacques P, Bleyaert P, Budzikiewicz H, Schäfer M, Höfte M (2013) New linear lipopeptides produced by *Pseudomonas cichorii* SF1-54 are involved in virulence, swarming motility, and biofilm formation. Mol Plant Microbe Interact 26(5):585–98. https://doi.org/10.1094/MPMI-11-12-0258-R

71. Huang CJ, Pauwelyn E, Ongena M, Debois D, Leclère V, Jacques P, Bleyaert P, Höfte M (2015) Characterization of cichopeptins, new phytotoxic cyclic lipodepsipeptides produced by *Pseudomonas cichorii* SF1-54 and their role in bacterial midrib rot disease of lettuce. Mol Plant Microbe Interact 28(9):1009–1022. https://doi.org/10.1094/MPMI-03-15-0061-R

72. Guntaka NS, Healy AR, Crawford JM, Herzon SB, Bruner SD (2017) Structure and functional analysis of ClbQ, an unusual intermediate-releasing thioesterase from the colibactin biosynthetic pathway. ACS Chem Biol 12(10):2598–2608. https://doi.org/10.1021/acschembio.7b00479

73. El-Sayed Ahmed MAE, Zhong LL, Shen C, Yang Y, Doi Y, Tian GB (2020) Colistin and its role in the era of antibiotic resistance: an extended review (2000–2019). Emerg Microbes Infect 9(1):868–885. https://doi.org/10.1080/22221751.2020.1754133

74. Tambadou F, Caradec T, Gagez AL, Bonnet A, Sopéna V, Bridiau N, Thiéry V, Didelot S, Barthélémy C, Chevrot R (2015) Characterization of the colistin (polymyxin E1 and E2) biosynthetic gene cluster. Arch Microbiol 197:521–532. https://doi.org/10.1007/s00203-015-1084-5

75. Erol O, Schäberle TF, Schmitz A, Rachid S, Gurgui C, El Omari M, Lohr F, Kehraus S, Piel J, Müller R, König GM (2010) Biosynthesis of the myxobacterial antibiotic corallopyronin A. Chembiochem 11:1253–1265. https://doi.org/10.1002/cbic.201000085

76. Couch R, O'Connor SE, Seidle H, Walsh CT, Parry R (2004) Characterization of CmaA, an adenylation-thiolation didomain enzyme involved in the biosynthesis of coronatine. J Bacteriol 186:35–42. https://doi.org/10.1128/JB.186.1.35-42.2004

77. Strano CP, Bella P, Licciardello G, Fiore A, Lo Piero AR, Fogliano V, Venturi V, Catara V (2015) *Pseudomonas corrugata* crpCDE is part of the cyclic lipopeptide corpeptin biosynthetic gene cluster and is involved in bacterial virulence in tomato and in hypersensitive response in Nicotiana benthamiana. Mol Plant Pathol 16(5):495–506. https://doi.org/10.1111/mpp.12207

78. Müller S, Rachid S, Hoffmann T, Surup F, Volz C, Zaburannyi N, Müller R (2014) Biosynthesis of crocacin involves an unusual hydrolytic release domain showing similarity to condensation domains. Chem Bio 21:855–865. https://doi.org/10.1016/j.chembiol.2014.05.012

79. Viehrig K, Surup F, Harmrolfs K, Jansen R, Kunze B, Müller R (2013) Concerted action of P450 plus helper protein to form the amino-hydroxy-piperidone moiety of the potent protease inhibitor crocapeptin. J Am Chem Soc 135:16885–16894. https://doi.org/10.1021/ja4047153

80. Baars O, Zhang X, Gibson MI, Stone AT, Morel FMM, Seyedsayamdost MR (2018) Crochelins: siderophores with an unprecedented iron-chelating moiety from the nitrogen-fixing bacterium *Azotobacter chroococcum*. Angew Chem Int Ed Engl 57(2):536–541. https://doi.org/10.1002/anie.201709720

81. Kreutzer MF, Kage H, Nett M (2012) Structure and biosynthetic assembly of cupriachelin, a photoreactive siderophore from the bioplastic producer *Cupriavidus necator* H16. J Am Chem Soc 134(11):5415–5422. https://doi.org/10.1021/ja300620z

82. Baumann S, Herrmann J, Raju R, Steinmetz H, Mohr KI, Hüttel S, Harmrolfs K, Stadler M, Müller R (2014) Cystobactamids: myxobacterial topoisomerase inhibitors exhibiting potent antibacterial activity. Angew Chem Int Ed Engl. 53(52):14605–14609. https://doi.org/10.1002/anie.201409964

83. Etzbach L, Plaza A, Garcia R, Baumann S, Müller R (2014) Cystomanamides: structure and biosynthetic pathway of a family of glycosylated lipopeptides from myxobacteria. Org Lett 16(9):2414–2417. https://doi.org/10.1021/ol500779s

84. Feng Z, Qi J, Tsuge T, Oba Y, Kobayashi T, Suzuki Y, Sakagami Y, Ojika M (2005) Construction of a bacterial artificial chromosome library for a myxobacterium of the genus *Cystobacter* and characterization of an antibiotic biosynthetic gene cluster. Biosci Biotechnol Biochem 6:1372–1380. https://doi.org/10.1271/bbb.69.1372

85. Tejman-Yarden N, Robinson A, Davidov Y, Shulman A, Varvak A, Reyes F, Rahav G, Nissan I (2019) Delftibactin-A, a non-ribosomal peptide with broad antimicrobial activity. Front Microbiol 10:2377. https://doi.org/10.3389/fmicb.2019.02377

86. Xu Y, Kersten RD, Nam SJ, Lu L, Al-Suwailem AM, Zheng H, Fenical W, Dorrestein PC, Moore BS, Qian PY (2012) Bacterial biosynthesis and maturation of the didemnin anti-cancer agents. J Am Chem Soc 134(20):8625–8632. https://doi.org/10.1021/ja301735a

87. Carvalho R, Reid R, Viswanathan N, Gramajo H, Julien B (2005) The biosynthetic genes for disorazoles, potent cytotoxic compounds that disrupt microtubule formation. Gene 359:91–98. https://doi.org/10.1016/j.gene.2005.06.003

88. Meiser P, Weissman KJ, Bode HB, Krug D, Dickschat JS, Sandmann A, Müller R (2008) DKxanthene biosynthesis--understanding the basis for diversity-oriented synthesis in myxobacterial secondary metabolism. Chem Biol 15(8):771–781. https://doi.org/10.1016/j.chembiol.2008.06.005

89. Rath CM, Janto B, Earl J, Ahmed A, Hu FZ, Hiller L, Dahlgren M, Kreft R, Yu F, Wolff JJ, Kweon HK, Christiansen MA, Håkansson K, Williams RM, Ehrlich GD, Sherman DH (2011) Meta-omic characterization of the marine invertebrate microbial consortium that produces the chemotherapeutic natural product ET-743. ACS Chem Bio 6:1244–1256. https://doi.org/10.1021/cb200244t

90. Westman EL, Yan M, Waglechner N, Koteva K, Wright GD (2013) Self resistance to the atypical cationic antimicrobial peptide edeine of *Brevibacillus brevis* Vm4 by the N-acetyltransferase EdeQ. Chem Biol 20:983–890. https://doi.org/10.1016/j.chembiol.2013.06.010

91. Müller A, Münch D, Schmidt Y, Reder-Christ K, Schiffer G, Bendas G, Gross H, Sahl HG, Schneider T, Brötz-Oesterhelt H (2012) Lipodepsipeptide empedopeptin inhibits cell wall biosynthesis through Ca^{2+}-dependent complex formation with peptidoglycan precursors. J Biol Chem 287(24):20270–20280. https://doi.org/10.1074/jbc.M112.369561

92. Niehs SP, Dose B, Scherlach K, Pidot SJ, Stinear TP, Hertweck C (2019) Genome mining reveals endopyrroles from a nonribosomal peptide assembly line triggered in fungal-bacterial symbiosis. ACS Chem Biol 14(8):1811–1818. https://doi.org/10.1021/acschembio.9b00406

93. Raymond KN, Dertz EA, Kim SS (2003) Enterobactin: an archetype for microbial iron transport. Proc Natl Acad Sci USA 100(7):3584–3588. https://doi.org/10.1073/pnas.0630018100

94. Vallet-Gely I, Novikov A, Augusto L, Liehl P, Bolbach G, Péchy-Tarr M, Cosson P, Keel C, Caroff M, Lemaitre B (20101) Association of hemolytic activity of *Pseudomonas entomophila,* a versatile soil bacterium, with cyclic lipopeptide production. Appl Environ Microbiol 76(3):910–921. https://doi.org/10.1128/AEM.02112-09

95. Molnár I, Schupp T, Ono M, Zirkle R, Milnamow M, Nowak-Thompson B, Engel N, Toupet C, Stratmann A, Cyr DD, Gorlach J, Mayo JM, Hu A, Goff S, Schmid J, Ligon JM (2000) The biosynthetic gene cluster for the microtubule-stabilizing agents epothilones A and B from *Sorangium cellulosum* So ce90. Chem Biol 7(2):97–109. https://doi.org/10.1016/s1074-5521(00)00075-2

96. Heather Z, Holden MT, Steward KF, Parkhill J, Song L, Challis GL, Robinson C, Davis-Poynter N, Waller AS (2008) A novel streptococcal integrative conjugative element involved in iron acquisition. Mol Microbiol 70(5):1274–1292. https://doi.org/10.1111/j.1365-2958.2008.06481.x

97. Fuchs SW, Grundmann F, Kurz M, Kaiser M, Bode HB (2014) Fabclavines: bioactive peptide-polyketide-polyamino hybrids from Xenorhabdus. Chembiochem 15:512–516. https://doi.org/ 10.1002/cbic.201300802

98. Vinnik V, Zhang F, Park H, Cook TB, Throckmorton K, Pfleger BF, Bugni TS, Thomas MG (2021) Structural and biosynthetic analysis of the fabrubactins, unusual siderophores from *Agrobacterium fabrum* Strain C58. ACS Chem Biol 16(1):125–135. https://doi.org/10.1021/acschembio.0c00809

99. Liu H, Gao L, Han J, Ma Z, Lu Z, Dai C, Zhang C, Bie X (2016) Biocombinatorial synthesis of novel lipopeptides by COM domain-mediated reprogramming of the plipastatin NRPS complex. Front Microbiol 7:1801. https://doi.org/10.3389/fmicb.2016.01801

100. Proschak A, Lubuta P, Grün P, Löhr F, Wilharm G, De Berardinis V, Bode HB (2013) Structure and biosynthesis of fimsbactins A–F, siderophores from *Acinetobacter baumannii* and *Acinetobacter baylyi*. Chembiochem 14:633–638. https://doi.org/10.1002/cbic.201200764

101. Cheng YQ, Yang M, Matter AM (2007) Characterization of a gene cluster responsible for the biosynthesis of anticancer agent FK228 in *Chromobacterium violaceum* No. 968. Appl Environ Microbiol 73(11):3460–3469. https://doi.org/10.1128/AEM.01751-06

102. Crüsemann M, Reher R, Schamari I, Brachmann AO, Ohbayashi T, Kuschak M, Malfacini D, Seidinger A, Pinto-Carbó M, Richarz R, Reuter T, Kehraus S, Hallab A, Attwood M, Schiöth HB, Mergaert P, Kikuchi Y, Schäberle TF, Kostenis E, Wenzel D, Müller CE, Piel J, Carlier A, Eberl L, König GM (2018) Heterologous expression, biosynthetic studies, and ecological function of the selective Gq-signaling inhibitor FR900359. Angew Chem Int Ed Eng 57:836–840. https://doi.org/10.1002/ anie.201707996

103. Zhang F, He HY, Tang MC, Tang YM, Zhou Q, Tang GL (2011) Cloning and elucidation of the FR901464 gene cluster revealing a complex acyltransferase-less polyketide synthase using glycerate

as starter units. J Am Chem Soc 133:2452–62. https://doi.org/10.1021/ja105649g

104. Eustáquio AS, Janso JE, Ratnayake AS, O'Donnell CJ, Koehn FE (2014) Spliceostatin hemiketal biosynthesis in *Burkholderia* spp. is catalyzed by an iron/α-ketoglutarate-dependent dioxygenase. Proc Natl Acad Sci USA 111:E3376–E3385. https://doi.org/10.1073/pnas.1408300111

105. Jenul C, Sieber S, Daeppen C, Mathew A, Lardi M, Pessi G, Hoepfner D, Neuburger M, Linden A, Gademann K, Eberl L (2018) Biosynthesis of fragin is controlled by a novel quorum sensing signal. Nat Commun 9:1297. https://doi.org/10.1038/s41467-018-03690-2.

106. Stow PR, Reitz ZL, Johnstone TC, Butler A (2021) Genomics-driven discovery of chiral triscatechol siderophores with enantiomeric Fe(iii) coordination. Chem Sci 12(37):12485–12493. https://doi.org/10.1039/d1sc03541j

107. Panter F, Krug D, Müller R (2019) Novel methoxymethacrylate natural products uncovered by statistics-based mining of the *Myxococcus fulvus* secondary metabolome. ACS Chem Biol 14:88–98. https://doi.org/10.1021/acschembio.8b00948

108. Vater J, Herfort S, Doellinger J, Weydmann M, Borriss R, Lasch P (2018) Genome mining of the Lipopeptide biosynthesis of *Paenibacillus polymyxa* E681 in combination with mass spectrometry: discovery of the lipoheptapeptide paenilipoheptin. Chembiochem 19(7):744–753. https://doi.org/10.1002/cbic.201700615

109. Jahanshah G, Yan Q, Gerhardt H, Pataj Z, Lämmerhofer M, Pianet I, Josten M, Sahl HG, Silby MW, Loper JE, Gross H (2019) Discovery of the cyclic lipopeptide gacamide A by genome mining and repair of the defective GacA regulator in *Pseudomonas fluorescens* Pf0-1. J Nat Prod 82:301–308. https://doi.org/10.1021/acs.jnatprod.8b00747

110. Dashti Y, Nakou IT, Mullins AJ, Webster G, Jian X, Mahenthiralingam E, Challis GL (2020) Discovery and biosynthesis of bolagladins: unusual lipodepsipeptides from *Burkholderia gladioli*

clinical isolates. Angew Chem Int Ed Engl 59(48):21553–21561. https://doi.org/10.1002/anie.202009110

111. Schellenberg B, Bigler L, Dudler R (2007) Identification of genes involved in the biosynthesis of the cytotoxic compound glidobactin from a soil bacterium. Environ Microbiol 9:1640–1650. https://doi.org/ 10.1111/j.1462-2920.2007.01278.x

112. Chen H, Zhong L, Zhou H, Sun T, Zhong G, Tu Q, Zhuang Y, Bai X, Wang X, Xu J, Xia L, Shen Y, Zhang Y, Bian X (2022) Biosynthesis of glidomides and elucidation of different mechanisms for formation of *β*-OH amino acid building blocks. Angew Chem Int Ed Engl 61:e202203591. https://doi.org/10.1002/anie.202203591

113. Wang X, Zhou H, Chen H, Jing X, Zheng W, Li R, Sun T, Liu J, Fu J, Huo L, Li YZ, Shen Y, Ding X, Müller R, Bian X, Zhang Y (2018) Discovery of recombinases enables genome mining of cryptic biosynthetic gene clusters in *Burkholderiales* species. Proc Natl Acad Sci USA 115:E4255–E4263. https://doi.org/10.1073/pnas.1720941115

114. Hermenau R, Ishida K, Gama S, Hoffmann B, Pfeifer-Leeg M, Plass W, Mohr JF, Wichard T, Saluz HP, Hertweck C (2018) Gramibactin is a bacterial siderophore with a diazeniumdiolate ligand system. Nat Chem Biol 14(9):841–843. https://doi.org/10.1038/s41589-018-0101-9

115. Hermenau R, Mehl JL, Ishida K, Dose B, Pidot SJ, Stinear TP, Hertweck C (2019) Genomics-driven discovery of NO-donating diazeniumdiolate siderophores in diverse plant-associated bacteria. Angew Chem Int Ed Engl 58:13024–13029. https://doi.org/10.1002/anie.201906326

116. Mogi T, Kita K (2009) Gramicidin S and polymyxins: the revival of cationic cyclic peptide antibiotics. Cell Mol Life Sci 66(23):3821–3826. https://doi.org/10.1007/s00018-009-0129-9

117. Zheng W, Wang X, Zhou H, Zhang Y, Li A, Bian X (2020) Establishment of recombineering genome editing system in *Paraburkholderia megapolitana* empowers activation of silent biosynthetic gene

clusters. Microb Biotechnol 13(2):397–405. https://doi.org/10.1111/1751-7915.13535

118. Sun Y, Tomura T, Sato J, Iizuka T, Fudou R, Ojika M (2016) Isolation and biosynthetic analysis of haliamide, a new PKS-NRPS hybrid metabolite from the marine myxobacterium *Haliangium ochraceum*. Molecules 21:59. https://doi.org/10.3390/molecules21010059

119. Zhong L, Diao X, Zhang N, Li F, Zhou H, Chen H, Bai X, Ren X, Zhang Y, Wu D, Bian X (2021) Engineering and elucidation of the lipoinitiation process in nonribosomal peptide biosynthesis. Nature Commun 12:296. https://doi.org/10.1038/s41467-020-20548-8

120. Bosello M, Zeyadi M, Kraas FI, Linne U, Xie X, Marahiel MA (2013) Structural characterization of the heterobactin siderophores from *Rhodococcus erythropolis* PR4 and elucidation of their biosynthetic machinery. J Nat Prod 76(12):2282–2290. https://doi.org/10.1021/np4006579

121. Matthijs S, Brandt N, Ongena M, Achouak W, Meyer JM, Budzikiewicz H (2016) Pyoverdine and histicorrugatin-mediated iron acquisition in *Pseudomonas thivervalensis*. Biometals 29(3):467–485. https://doi.org/10.1007/s10534-016-9929-1

122. Qin Z, Baker AT, Raab A, Huang S, Wang T, Yu Y, Jaspars M, Secombes CJ, Deng H (2013) The fish pathogen *Yersinia ruckeri* produces holomycin and uses an RNA methyltransferase for self-resistance. J Biol Chem 288(21):14688–14697. https://doi.org/10.1074/jbc.M112.448415

123. Xu L, Wu P, Wright SJ, Du L, Wei X (2015) Bioactive polycyclic tetramate macrolactams from *Lysobacter enzymogenes* and their absolute configurations by theoretical ECD calculations. J Nat Prod 7:1841–1847. https://doi.org/10.1021/acs.jnatprod.5b00099

124. Wirtz DA, Ludwig KC, Arts M, Marx CE, Krannich S, Barac P, Kehraus S, Josten M, Henrichfreise B, Mueller Anna, Koenig GM, Peoples AJ, Nitti Anthony, Spoering AL, Ling LL, Lewis K, Cruesemann M, Schneider T (2021) Biosynthesis and mechanism of action of the cell wall targeting antibiotic hypeptin. Angew Chem Int Ed Engl 60:13579–13586. https://doi.org/10.1002/anie.

202102224

125. Dose B, Niehs SP, Scherlach K, Flórez LV, Kaltenpoth M, Hertweck C (2018) Unexpected bacterial origin of the antibiotic icosalide: two-tailed depsipeptide assembly in multifarious *Burkholderia* symbionts. ACS Chem Biol 13(9):2414–2420. https://doi.org/10.1021/acschembio.8b00600

126. Pang B, Chen Y, Gan F, Yan C, Jin L, Gin JW, Petzold CJ, Keasling JD (2020) Investigation of indigoidine synthetase reveals a conserved active-site base residue of nonribosomal peptide synthetase oxidases. J Am Chem Soc 142:10931–10935. https://doi.org/10.1021/jacs.0c04328

127. Tsuge K, Akiyama T, Shoda M (2001) Cloning, sequencing, and characterization of the iturin A operon. J Bacteriol 183(21):6265–6273. https://doi.org/10.1128/JB.183.21.6265-6273.2001

128. Wang X, Luo C, Chen Z (2012) Genome sequence of the plant growth-promoting rhizobacterium *Bacillus* sp. strain 916. J Bacteriol 194(19):5467–5468. https://doi.org/10.1128/JB.01266-12

129. Fischer D, Gessner G, Fill TP, Barnett R, Tron K, Dornblut K, Kloss F, Stallforth P, Hube B, Heinemann SH, Hertweck C, Scherlach K, Brunke S (2019) Disruption of membrane integrity by the bacterium-derived antifungal jagaricin. Antimicrob Agents Chemother 63:e00707-19. https://doi.org/ 10.1128/AAC.00707-19

130. Arp J, Götze S, Mukherji R, Mattern DJ, García-Altares M, Klapper M, Brock DA, Brakhage AA, Strassmann JE, Queller DC, Bardl B, Willing K, Peschel G, Stallforth P (2018) Synergistic activity of cosecreted natural products from amoebae-associated bacteria. Proc Natl Acad Sci USA. 115(15):3758–3763. https://doi.org/10.1073/pnas.1721790115

131. Uytterhoeven B, Lathouwers T, Voet M, Michiels CW, Lavigne R (2016) A protein interaction map of the kalimantacin biosynthesis assembly line. Front Microbio 7:1726. https://doi.org/10.3389/fmicb. 2016. 01726

132. Tse H, Gu Q, Sze KH, Chu IK, Kao RY, Lee KC, Lam CW, Yang D, Tai SS, Ke Y, Chan E, Chan

WM, Dai J, Leung SP, Leung SY, Yuen KY (2017) A tricyclic pyrrolobenzodiazepine produced by *Klebsiella oxytoca* is associated with cytotoxicity in antibiotic-associated hemorrhagic colitis. J Biol Chem 292(47):19503–19520. https://doi.org/10.1074/jbc.M117.791558

133. Bode HB, Brachmann AO, Jadhav KB, Seyfarth L, Dauth C, Fuchs SW, Kaiser M, Waterfield NR, Sack H, Heinemann SH, Arndt HD (2015) Structure elucidation and activity of kolossin A, the D-/L-pentadecapeptide product of a giant nonribosomal peptide synthetase. Angew Chem Int Ed Engl 54(35):10352–10355. https://doi.org/10.1002/anie.201502835

134. Evans BS, Ntai I, Chen Y, Robinson SJ, Kelleher NL (2011) Proteomics-based discovery of koranimine, a cyclic imine natural product. J Am Chem Soc 133(19):7316–7319. https://doi.org/10.1021/ja2015795

135. Béchet M, Caradec T, Hussein W, Abderrahmani A, Chollet M, Leclère V, Dubois T, Lereclus D, Pupin M, Jacques P (2012) Structure, biosynthesis, and properties of kurstakins, nonribosomal lipopeptides from *Bacillus* spp. Appl Microbiol Biotechnol 95(3):593–600. https://doi.org/10.1007/s00253-012-4181-2

136. Kačar D, Schleissner C, Cañedo LM, Rodríguez P, de la Calle F, Galán B, García JL (2019) Genome of *Labrenzia* sp. PHM005 reveals a complete and active trans-AT PKS gene cluster for the biosynthesis of labrenzin. Front Microbiol 10:2561. https://doi.org/10.3389/fmicb.2019.02561

137. Flórez LV, Scherlach K, Miller IJ, Rodrigues A, Kwan JC, Hertweck C, Kaltenpoth M (2018) An antifungal polyketide associated with horizontally acquired genes supports symbiont-mediated defense in *Lagria villosa* beetles. Nat Commun 9:2478. https://doi.org/10.1038/s41467-018-04955-6

138. Li YX, Zhong Z, Zhang WP, Qian PY (2018) Discovery of cationic nonribosomal peptides as Gram-negative antibiotics through global genome mining. Nat Commun 9:3273. https://doi.org/10.1038/s41467-018-05781-6

139. Li S, Wu X, Zhang L, Shen Y, Du L (2017) Activation of a cryptic gene cluster in *Lysobacter enzymogenes* reveals a module/domain portable mechanism of nonribosomal peptide synthetases in the biosynthesis of pyrrolopyrazines. Org Lett 19(19):5010–5013. https://doi.org/10.1021/acs.orglett.7b01611

140. Kopp M, Irschik H, Gemperlein K, Buntin K, Meiser P, Weissman KJ, Bode HB, Müller R (2011) Insights into the complex biosynthesis of the leupyrrins in *Sorangium cellulosum* So ce690. Mol Biosyst 7(5):1549–1563. https://doi.org/10.1039/c0mb00240b

141. Konz D, Doekel S, Marahiel MA (1999) Molecular and biochemical characterization of the protein template controlling biosynthesis of the lipopeptide lichenysin. J Bacteriol 181(1):133–140. https://doi.org/10.1128/JB.181.1.133-140.1999

142. Kessler N, Schuhmann H, Morneweg S, Linne U, Marahiel MA (2004) The linear pentadecapeptide gramicidin is assembled by four multimodular nonribosomal peptide synthetases that comprise 16 modules with 56 catalytic domains. J Biol Chem 279:7413–7419. https://doi.org/10.1074/jbc.M309658200

143. Luo C, Liu X, Zhou X, Guo J, Truong J, Wang X, Zhou H, Li X, Chen Z (2015) Unusual biosynthesis and structure of locillomycins from *Bacillus subtilis* 916. Appl Environ Microbiol 81:6601–6609. https://doi.org/10.1128/AEM.01639-15

144. Omoboye OO, Oni FE, Batool H, Yimer HZ, De Mot R, Höfte M (2019) *Pseudomonas* cyclic lipopeptides suppress the rice blast fungus *Magnaporthe oryzae* by induced resistance and direct antagonism. Front Plant Sci 10:901. https://doi.org/10.3389/fpls.2019.00901

145. Zipperer A, Konnerth MC, Laux C, Berscheid A, Janek D, Weidenmaier C, Burian M, Schilling NA, Slavetinsky C, Marschal M, Willmann M, Kalbacher H, Schittek B, Brötz-Oesterhelt H, Grond S, Peschel A, Krismer B (2016) Human commensals producing a novel antibiotic impair pathogen

colonization. Nature 535(7613):511–516. https://doi.org/10.1038/nature18634.

146. Fu J, Bian X, Hu S, Wang H, Huang F, Seibert PM, Plaza A, Xia L, Müller R, Stewart AF, Zhang Y (2012) Full-length RecE enhances linear-linear homologous recombination and facilitates direct cloning for bioprospecting. Nat Biotechnol 30(5):440–446. https://doi.org/10.1038/nbt.2183

147. Nollmann FI, Dauth C, Mulley G, Kegler C, Kaiser M, Waterfield NR, Bode HB (2015) Insect-specific production of new GameXPeptides in *Photorhabdus luminescens* TTO1, widespread natural products in entomopathogenic bacteria. Chembiochem 16:205–208. https://doi.org/10.1002/cbic.201402603

148. Hou J, Robbel L, Marahiel MA (2011) Identification and characterization of the lysobactin biosynthetic gene cluster reveals mechanistic insights into an unusual termination module architecture. Chem Biol 18(5):655–664. https://doi.org/10.1016/j.chembiol.2011.02.012

149. Hamamoto H, Urai M, Ishii K, Yasukawa J, Paudel A, Murai M, Kaji T, Kuranaga T, Hamase K, Katsu T, Su J, Adachi T, Uchida R, Tomoda H, Yamada M, Souma M, Kurihara H, Inoue M, Sekimizu K (2015) Lysocin E is a new antibiotic that targets menaquinone in the bacterial membrane. Nat Chem Biol 11(2):127–133. https://doi.org/10.1038/nchembio.1710

150. Panthee S, Hamamoto H, Suzuki Y, Sekimizu K (2017) In silico identification of lysocin biosynthetic gene cluster from *Lysobacter* sp. RH2180-5. J Antibiot (Tokyo) 70(2):204–207. https://doi.org/10.1038/ja.2016.102

151. Keller L, Plaza A, Dubiella C, Groll M, Kaiser M, Müller R (2015) Macyranones: structure, biosynthesis, and binding mode of an unprecedented epoxyketone that targets the 20S proteasome. J Am Chem Soc 137(25):8121–8130. https://doi.org/10.1021/jacs.5b03833

152. Franke J, Ishida K, Ishida-Ito M, Hertweck C (2013) Nitro versus hydroxamate in siderophores of pathogenic bacteria: effect of missing hydroxylamine protection in malleobactin biosynthesis. Angew

Chem Int Ed Engl 52:8271–8275. https://doi.org/10.1002/anie.201303196

153. Helfrich EJN, Vogel CM, Ueoka R, Schäfer M, Ryffel F, Müller DB, Probst S, Kreuzer M, Piel J, Vorholt JA (2018) Bipartite interactions, antibiotic production and biosynthetic potential of the Arabidopsis leaf microbiome. Nat Microbiol 3(8):909–919. https://doi.org/10.1038/s41564-018-0200-0

154. de Bruijn I, de Kock MJ, de Waard P, van Beek TA, Raaijmakers JM (2008) Massetolide A biosynthesis in *Pseudomonas fluorescens*. J Bacteriol 190(8):2777–2789. https://doi.org/10.1128/JB.01563-07

155. Diettrich J, Kage H, Nett M (2019) Genomics-inspired discovery of massiliachelin, an agrochelin epimer from *Massilia* sp. NR 4-1. Beilstein J Org Chem 15:1298–1303. https://doi.org/10.3762/bjoc.15.128

156. Weinig S, Hecht HJ, Mahmud T, Müller R (2003) Melithiazol biosynthesis: further insights into myxobacterial PKS/NRPS systems and evidence for a new subclass of methyl transferases. Chem Biol 10:939–952. https://doi.org/10.1016/j.chembiol.2003.09.012

157. Kage H, Kreutzer MF, Wackler B, Hoffmeister D, Nett M (2013) An iterative type I polyketide synthase initiates the biosynthesis of the antimycoplasma agent micacocidin. Chem Biol 20(6):764–71. https://doi.org/10.1016/j.chembiol.2013.04.010

158. Hoffmann T, Müller S, Nadmid S, Garcia R, Müller R (2013) Microsclerodermins from terrestrial myxobacteria: an intriguing biosynthesis likely connected to a sponge symbiont. J Am Chem Soc 135(45):16904–16911. https://doi.org/10.1021/ja4054509

159. Zvanych R, Lukenda N, Li X, Kim JJ, Tharmarajah S, Magarvey NA (2015) Systems biosynthesis of secondary metabolic pathways within the oral human microbiome member *Streptococcus mutans*. Mol Biosyst 11:97–104. https://doi.org/10.1039/c4mb00406j

160. Duitman EH, Hamoen LW, Rembold M, Venema G, Seitz H, Saenger W, Bernhard F, Reinhardt R, Schmidt M, Ullrich C, Stein T, Leenders F, Vater J (1999) The mycosubtilin synthetase of Bacillus subtilis ATCC6633: a multifunctional hybrid between a peptide synthetase, an amino transferase, and a fatty acid synthase. Proc Natl Acad Sci USA 96(23):13294–13299. https://doi.org/10.1073/pnas.96.23.13294

161. Guez JS, Coucheney F, Guy J, Béchet M, Fontanille P, Chihib NE, Niehren J, Coutte F, Jacques P (2022) Bioinformatics modelling and metabolic engineering of the branched chain amino acid pathway for specific production of mycosubtilin isoforms in *Bacillus subtilis*. Metabolites 12(2):107. https://doi.org/10.3390/metabo12020107

162. Silakowski B, Nordsiek G, Kunze B, Blöcker H, Müller R (2001) Novel features in a combined polyketide synthase/non-ribosomal peptide synthetase: the myxalamid biosynthetic gene cluster of the myxobacterium *Stigmatella aurantiaca* Sga15. Chem Biol 8:59–69. https://doi.org/10.1016/ s1074-5521(00)00056-9

163. Gaitatzis N, Kunze B, Müller R (2001) In vitro reconstitution of the myxochelin biosynthetic machinery of *Stigmatella aurantiaca* Sg a15: Biochemical characterization of a reductive release mechanism from nonribosomal peptide synthetases. Proc Natl Acad Sci USA 98:11136–11141. https://doi.org/10.1073/pnas.201167098

164. Okoth DA, Hug JJ, Garcia R, Müller R (2022) Discovery, Biosynthesis and Biological Activity of a Succinylated Myxochelin from the Myxobacterial Strain MSr12020. Microorganisms 10(10):1959. https://doi.org/10.3390/microorganisms10101959

165. Burgard C, Zaburannyi N, Nadmid S, Maier J, Jenke-Kodama H, Luxenburger E, Bernauer HS, Wenzel SC (2017) Genomics-guided exploitation of lipopeptide diversity in Myxobacteria. ACS Chem Biol 12:779–786. https://doi.org/10.1021/acschembio.6b00953

166. Cortina NS, Krug D, Plaza A, Revermann O, Müller R (2012) Myxoprincomide: a natural product from *Myxococcus xanthus* discovered by comprehensive analysis of the secondary metabolome. Angew Chem Int Ed Engl 51(3):811–816. https://doi.org/10.1002/anie.201106305

167. Sucipto H, Wenzel SC, Müller R (2013) Exploring chemical diversity of α-pyrone antibiotics: molecular basis of myxopyronin biosynthesis. Chembiochem 14:1581–1589. https://doi.org/10.1002/cbic. 201300289

168. Silakowski B, Schairer HU, Ehret H, Kunze B, Weinig S, Nordsiek G, Brandt P, Blöcker H, Höfle G, Beyer S, Müller R (1999) New lessons for combinatorial biosynthesis from myxobacteria. The myxothiazol biosynthetic gene cluster of *Stigmatella aurantiaca* DW4/3-1. J Biol Chem 274:37391–37399. https://doi.org/10.1074/jbc.274.52.37391

169. Simunovic V, Zapp J, Rachid S, Krug D, Meiser P, Müller R (2006) Myxovirescin A biosynthesis is directed by hybrid polyketide synthases/nonribosomal peptide synthetase, 3-hydroxy-3-methylglutaryl-CoA synthases, and trans-acting acyltransferases. Chembiochem 7: 1206–1220. https://doi.org/10.1002/cbic.200600075

170. Krastel P, Roggo S, Schirle M, Ross NT, Perruccio F, Aspesi P Jr, Aust T, Buntin K, Estoppey D, Liechty B, Mapa F, Memmert K, Miller H, Pan X, Riedl R, Thibaut C, Thomas J, Wagner T, Weber E, Xie X, Schmitt EK, Hoepfner D (2015) Nannocystin A: an elongation factor 1 inhibitor from Myxobacteria with differential anti-cancer properties. Angew Chem Int Ed Engl 54(35):10149–54. https://doi.org/10.1002/anie.201505069

171. Cai X, Challinor VL, Zhao L, Reimer D, Adihou H, Grün P, Kaiser M, Bode HB (2017) Biosynthesis of the antibiotic nematophin and its elongated derivatives in entomopathogenic bacteria. Org Lett 19:806–809. https://doi.org/10.1021/acs.orglett.6b03796

172. Kunyavskaya O, Tagirdzhanov AM, Caraballo-Rodríguez AM, Nothias LF, Dorrestein PC,

Korobeynikov A, Mohimani H, Gurevich A (2021) Nerpa: a tool for discovering biosynthetic gene clusters of bacterial nonribosomal peptides. Metabolites 11(10):693. https://doi.org/10.3390/metabo11100693

173. Zhang Q, Xiao X, Zheng J, Li M, Yu M, Ping F, Wang T, Wang X (2019) Influence of maternal inulin-type prebiotic intervention on glucose metabolism and gut microbiota in the offspring of C57BL mice. Front Endocrinol (Lausanne) 10:675. https://doi.org/10.3389/fendo.2019.00675

174. Michelsen CF, Watrous J, Glaring MA, Kersten R, Koyama N, Dorrestein PC, Stougaard P (2015) Nonribosomal peptides, key biocontrol components for *Pseudomonas fluorescens* In5, isolated from a Greenlandic suppressive soil. mBio 6(2):e00079. https://doi.org/10.1128/mBio.00079-15

175. Scott TA, Heine D, Qin Z, Wilkinson B (2017) An L-threonine transaldolase is required for L-threo-β-hydroxy-α-amino acid assembly during obafluorin biosynthesis. Nat Commun 8:15935. https://doi.org/10.1038/ncomms15935

176. Velkov T, Gallardo-Godoy A, Swarbrick JD, Blaskovich MAT, Elliott AG, Han M, Thompson PE, Roberts KD, Huang JX, Becker B, Butler MS, Lash LH, Henriques ST, Nation RL, Sivanesan S, Sani MA, Separovic F, Mertens H, Bulach D, Seemann T, Owen J, Li J, Cooper MA (2018) Structure, function, and biosynthetic origin of octapeptin antibiotics active against extensively drug-resistant gram-negative bacteria. Cell Chem Biol 25:380–391. https://doi.org/10.1016/j.chembiol.2018.01.005

177. Pantel L, Florin T, Dobosz-Bartoszek M, Racine E, Sarciaux M, Serri M, Houard J, Campagne JM, de Figueiredo RM, Midrier C, Gaudriault S, Givaudan A, Lanois A, Forst S, Aumelas A, Cotteaux-Lautard C, Bolla JM, Vingsbo Lundberg C, Huseby DL, Hughes D, Villain-Guillot P, Mankin AS, Polikanov YS, Gualtieri M (2018) Odilorhabdins, antibacterial agents that cause miscoding by binding at a new ribosomal site. Mol Cell 70:83–94 https://doi.org/10.1016/j.molcel.2018.03.001

178. Gross H, Stockwell VO, Henkels MD, Nowak-Thompson B, Loper JE, Gerwick WH (2007) The genomisotopic approach: a systematic method to isolate products of orphan biosynthetic gene clusters. Chem Biol 14(1):53–63. https://doi.org/10.1016/j.chembiol.2006.11.007

179. Ma Z, Geudens N, Kieu NP, Sinnaeve D, Ongena M, Martins JC, Höfte M (2016) Biosynthesis, chemical structure, and structure-activity relationship of orfamide lipopeptides produced by *Pseudomonas protegens* and related species. Front Microbiol 7:382. https://doi.org/10.3389/fmicb.2016.00382

180. Agnoli K, Lowe CA, Farmer KL, Husnain SI, Thomas MS (2006) The ornibactin biosynthesis and transport genes of *Burkholderia cenocepacia* are regulated by an extracytoplasmic function sigma factor which is a part of the Fur regulon. J Bacteriol 188(10):3631–3644. https://doi.org/10.1128/JB.188.10.3631-3644.2006

181. Hardy CD, Butler A (2019) Ambiguity of NRPS structure predictions: four bidentate chelating groups in the siderophore pacifibactin. J Nat Prod 82:990–997. https://doi.org/10.1021/acs.jnatprod.8b01073

182. Guo Y, Huang E, Yuan C, Zhang L, Yousef AE (2012) Isolation of a *Paenibacillus* sp. strain and structural elucidation of its broad-spectrum lipopeptide antibiotic. Appl Environ Microbiol 78(9):3156–3165. https://doi.org/10.1128/AEM.07782-11

183. Wen Y, Wu X, Teng Y, Qian C, Zhan Z, Zhao Y, Li O (2011) Identification and analysis of the gene cluster involved in biosynthesis of paenibactin, a catecholate siderophore produced by *Paenibacillus elgii* B69. Environ Microbiol 13(10):2726–2737. https://doi.org/10.1111/j.1462-2920.2011.02542.x

184. Müller S, Garcia-Gonzalez E, Mainz A, Hertlein G, Heid NC, Mösker E, van den Elst H, Overkleeft HS, Genersch E, Süssmuth RD (2014) Paenilamicin: structure and biosynthesis of a hybrid nonribosomal peptide/polyketide antibiotic from the bee pathogen *Paenibacillus larvae*. Angew Chem

Int Ed Engl 53:10821–10825. https://doi.org/10.1002/anie.201404572

185. Sood S, Steinmetz H, Beims H, Mohr KI, Stadler M, Djukic M, von der Ohe W, Steinert M, Daniel R, Müller R (2014) Paenilarvins: Iturin family lipopeptides from the honey bee pathogen *Paenibacillus larvae*. Chembiochem 15(13):1947–1955. https://doi.org/10.1002/cbic.201402139

186. Kwan JC, Donia MS, Han AW, Hirose E, Haygood MG, Schmidt EW (2012) Genome streamlining and chemical defense in a coral reef symbiosis. Proc Natl Acad Sci USA 109(50):20655–20660. https://doi.org/10.1073/pnas.1213820109

187. Behsaz B, Bode E, Gurevich A, Shi YN, Grundmann F, Acharya D, Caraballo-Rodríguez AM, Bouslimani A, Panitchpakdi M, Linck A, Guan C, Oh J, Dorrestein PC, Bode HB, Pevzner PA, Mohimani H (2021) Integrating genomics and metabolomics for scalable non-ribosomal peptide discovery. Nat Commun 12:3225. https://doi.org/10.1038/s41467-021-23502-4

188. Liu Z,Wu X, Wang J,Huang F (2009) Molecular Evidences for the Biosynthesis of Pederin by Endosymbiont. Agricultural Sciences in China 11(8):1339–1350. https://doi.org/10.1016/S1671-2927(08)60346-6

189. Piel J, Höfer I, Hui D (2004) Evidence for a symbiosis island involved in horizontal acquisition of pederin biosynthetic capabilities by the bacterial symbiont of *Paederus fuscipes* beetles. J Bacteriol 186(5):1280–1286. https://doi.org/10.1128/JB.186.5.1280-1286.2004

190. Qian CD, Liu TZ, Zhou SL, Ding R, Zhao WP, Li O, Wu XC (2012) Identification and functional analysis of gene cluster involvement in biosynthesis of the cyclic lipopeptide antibiotic pelgipeptin produced by Paenibacillus elgii. BMC Microbiol 12:197. https://doi.org/10.1186/1471-2180-12-197

191. Jahns C, Hoffmann T, Müller S, Gerth K, Washausen P, Höfle G, Reichenbach H, Kalesse M, Müller R (2012) Pellasoren: structure elucidation, biosynthesis, and total synthesis of a cytotoxic secondary metabolite from Sorangium cellulosum. Angew Chem Int Ed Engl 51:5239–5243.

https://doi.org/10.1002/anie.201200327

192. Brinkmann S, Semmler S, Kersten C, Patras MA, Kurz M, Fuchs N, Hammerschmidt SJ, Legac J, Hammann PE, Vilcinskas A, Rosenthal PJ, Schirmeister T, Bauer A, Schäberle TF (2022) Identification, characterization, and synthesis of natural parasitic cysteine protease inhibitors: Pentacitidins are more potent falcitidin analogues. ACS Chem Biol 17(3):576–589. https://doi.org/10.1021/acschembio.1c00861

193. Park S, Hyun H, Lee JS, Cho K (2016) Identification of the phenalamide biosynthetic gene cluster in *Myxococcus stipitatus* DSM 14675. J Microbiol Biotechnol 26:1636–1642. https://doi.org/10.4014/jmb.1603. 03023

194. Zhao L, Awori RM, Kaiser M, Groß J, Opatz T, Bode HB (2019) Structure, biosynthesis, and bioactivity of photoditritide from *Photorhabdus temperata* Meg1. J Nat Prod 82:3499–3503. https://doi.org/10. 1021/acs.jnatprod.9b00932.

195. Zhao L, Bode HB (2019) Production of a photohexapeptide library from entomopathogenic *Photorhabdus asymbiotica* PB68.1. Org Biomol Chem 17:7858–7862. https://doi.org/10.1039/c9ob01489f

196. Zhao L, Vo TD, Kaiser M, Bode HB (2020) Phototemtide A, a Cyclic Lipopeptide Heterologously Expressed from *Photorhabdus temperata* Meg1, Shows Selective Antiprotozoal Activity. Chembiochem 21(9):1288–1292. https://doi.org/10.1002/cbic.201900665

197. Shi YM, Hirschmann M, Shi YN, Bode HB (2022) Cleavage off-loading and post-assembly-line conversions yield products with unusual termini during biosynthesis. ACS Chem Biol 17(8):2221–2228. https://doi.org/10.1021/acschembio.2c00367

198. Esmaeel Q, Pupin M, Kieu NP, Chataigné G, Béchet M, Deravel J, Krier F, Höfte M, Jacques P, Leclère V (2016) *Burkholderia* genome mining for nonribosomal peptide synthetases reveals a great

potential for novel siderophores and lipopeptides synthesis. Microbiologyopen 5(3):512–526. https://doi.org/ 10.1002/ mbo3.347

199. Souto A, Montaos MA, Rivas AJ, Balado M, Osorio CR, Rodríguez J, Lemos ML, Jiménez C (2012) Structure and biosynthetic assembly of piscibactin, a siderophore from *Photobacterium damselae* subsp. *piscicida*, predicted from genome analysis. Eur J Org Chem 2012:5693–5700. https://doi.org/10.1002/ejoc.201200818

200. Zachow C, Jahanshah G, de Bruijn I, Song C, Ianni F, Pataj Z, Gerhardt H, Pianet I, Lämmerhofer M, Berg G, Gross H, Raaijmakers JM (2015) The novel lipopeptide poaeamide of the endophyte *Pseudomonas poae* RE*1-1-14 is involved in pathogen suppression and root colonization. Mol Plant Microbe Interact 28(7):800–810. https://doi.org/10.1094/MPMI-12-14-0406-R

201. Choi SK, Park SY, Kim R, Kim SB, Lee CH, Kim JF, Park SH (2009) Identification of a polymyxin synthetase gene cluster of *Paenibacillus polymyxa* and heterologous expression of the gene in *Bacillus subtilis*. J Bacteriol 191(10):3350–3358. https://doi.org/10.1128/JB.01728-08

202. Shaheen M, Li J, Ross AC, Vederas JC, Jensen SE (2011) *Paenibacillus polymyxa* PKB1 produces variants of polymyxin B-type antibiotics. Chem Biol 18(12):1640–1648. https://doi.org/10.1016/ j.chembiol.2011.09.017

203. Galea CA, Han M, Zhu Y, Roberts K, Wang J, Thompson PE, L J, Velkov T (2017) Characterization of the polymyxin D synthetase biosynthetic cluster and product profile of *Paenibacillus polymyxa* ATCC 10401. J Nat Prod 80(5):1264–1274. https://doi.org/10.1021/acs.jnatprod.6b00807

204. Niu B, Vater J, Rueckert C, Blom J, Lehmann M, Ru JJ, Chen XH, Wang Q, Borriss R (2013) Polymyxin P is the active principle in suppressing phytopathogenic *Erwinia* spp. by the biocontrol rhizobacterium *Paenibacillus polymyxa* M-1. BMC Microbiol 13:137. https://doi.org/10.1186/1471-2180-13-137

205. Masschelein J, Mattheus W, Gao LJ, Moons P, Van Houdt R, Uytterhoeven B, Lamberigts C, Lescrinier E, Rozenski J, Herdewijn P, Aertsen A, Michiels C, Lavigne R (2013) A PKS/NRPS/FAS hybrid gene cluster from *Serratia plymuthica* RVH1 encoding the biosynthesis of three broad spectrum, zeamine-related antibiotics. PLoS One 8:e54143. https://doi.org/10.1371/journal.pone.0054143

206. Brachmann AO, Reimer D, Lorenzen W, Augusto Alonso E, Kopp Y, Piel J, Bode HB (2012) Reciprocal cross talk between fatty acid and antibiotic biosynthesis in a nematode symbiont. Angew Chem Int Ed Engl 51(48):12086–12089. https://doi.org/10.1002/anie.201205384

207. Kim D, Lee JS, Park YK, Kim JF, Jeong H, Oh TK, Kim BS, Lee CH (2007) Biosynthesis of antibiotic prodiginines in the marine bacterium *Hahella chejuensis* KCTC 2396. J Appl Microbio 102:937–944. https://doi.org/10.1111/j.1365-2672.2006.03172.x

208. Li W, Estrada-de los Santos P, Matthijs S, Xie GL, Busson R, Cornelis P, Rozenski J, De Mot R (2011) Promysalin, a salicylate-containing *Pseudomonas putida* antibiotic, promotes surface colonization and selectively targets other *Pseudomonas*. Chem Biol 18:1320–1330. https://doi.org/10.1016/j.chembiol.2011.08.006

209. Girard L, Geudens N, Pauwels B, Höfte M, Martins JC, De Mot R (2022) Transporter gene-mediated typing for detection and genome mining of lipopeptide-producing *Pseudomonas*. Appl Environ Microbiol 88(2):e0186921. https://doi.org/10.1128/AEM.01869-21

210. Oni FE, Geudens N, Adiobo A, Omoboye OO, Enow EA, Onyeka JT, Salami AE, De Mot R, Martins JC, Höfte M (2020) Biosynthesis and antimicrobial activity of pseudodesmin and viscosinamide cyclic lipopeptides produced by pseudomonads associated with the cocoyam rhizosphere. Microorganisms 8(7):1079. https://doi.org/10.3390/microorganisms8071079

211. Mercado-Blanco J, van der Drift KM, Olsson PE, Thomas-Oates JE, van Loon LC, Bakker PA (2001)

Analysis of the *pmsCEAB* gene cluster involved in biosynthesis of salicylic acid and the siderophore pseudomonine in the biocontrol strain *Pseudomonas fluorescens* WCS374. J Bacteriol 183:1909–1920. https://doi.org/10.1128/JB.183.6.1909-1920.2001

212. Ióca LP, Dai Y, Kunakom S, Diaz-Espinosa J, Krunic A, Crnkovic CM, Orjala J, Sanchez LM, Ferreira AG, Berlinck RGS, Eustáquio AS (2021) A family of nonribosomal peptides modulate collective behavior in *Pseudovibrio* bacteria isolated from marine sponges. Angew Chem Int Ed Engl 60(29):15891–15898. https://doi.org/10.1002/anie.202017320
213. Dubern JF, Coppoolse ER, Stiekema WJ, Bloemberg GV (2008) Genetic and functional characterization of the gene cluster directing the biosynthesis of putisolvin I and II in *Pseudomonas putida* strain PCL1445. Microbiology 154:2070–2083. https://doi.org/10.1099/mic.0.2008/016444-0
214. Ronnebaum TA, Lamb AL (2018) Nonribosomal peptides for iron acquisition: pyochelin biosynthesis as a case study. Curr Opin Struct Biol 53:1–11. https://doi.org/10.1016/j.sbi.2018.01.015
215. Nowak-Thompson B, Chaney N, Wing JS, Gould SJ, Loper JE (1999) Characterization of the pyoluteorin biosynthetic gene cluster of *Pseudomonas fluorescens* Pf-5. J Bacteriol 181:2166–2174. https://doi.org/10.1128/JB.181.7.2166-2174.1999
216. Ravel J, Cornelis P (2003) Genomics of pyoverdine-mediated iron uptake in pseudomonads. Trends Microbiol 11(5):195–200. https://doi.org/10.1016/s0966-842x(03)00076-3
217. Owen JG, Ackerley DF (2011) Characterization of pyoverdine and achromobactin in *Pseudomonas syringae* pv. *phaseolicola* 1448a. BMC Microbiol 11:218. https://doi.org/10.1186/1471-2180-11-218
218. Smith EE, Sims EH, Spencer DH, Kaul R, Olson MV (2005) Evidence for diversifying selection at the pyoverdine locus of *Pseudomonas aeruginosa*. J Bacteriol 187(6):2138–47. https://doi.org/10.1128/JB.187.6.2138-2147.2005
219. Chen WJ, Kuo TY, Hsieh FC, Chen PY, Wang CS, Shih YL, Lai YM, Liu JR, Yang YL, Shih MC

(2016) Involvement of type VI secretion system in secretion of iron chelator pyoverdine in *Pseudomonas taiwanensis*. Sci Rep 6:32950. https://doi.org/10.1038/srep32950

220. Parker DL, Lee SW, Geszvain K, Davis RE, Gruffaz C, Meyer JM, Torpey JW, Tebo BM (2014) Pyoverdine synthesis by the Mn(II)-oxidizing bacterium *Pseudomonas putida* GB-1. Front Microbiol 5:202. https://doi.org/10.3389/fmicb.2014.00202

221. Matthijs S, Laus G, Meyer JM, Abbaspour-Tehrani K, Schäfer M, Budzikiewicz H, Cornelis P (2009) Siderophore-mediated iron acquisition in the entomopathogenic bacterium *Pseudomonas entomophila* L48 and its close relative *Pseudomonas putida* KT2440. Biometals 22(6):951–964. https://doi.org/10.1007/s10534-009-9247-y.

222. Loper JE, Hassan KA, Mavrodi DV, Davis EW 2nd, Lim CK, Shaffer BT, Elbourne LD, Stockwell VO, Hartney SL, Breakwell K, Henkels MD, Tetu SG, Rangel LI, Kidarsa TA, Wilson NL, van de Mortel JE, Song C, Blumhagen R, Radune D, Hostetler JB, Brinkac LM, Durkin AS, Kluepfel DA, Wechter WP, Anderson AJ, Kim YC, Pierson LS 3rd, Pierson EA, Lindow SE, Kobayashi DY, Raaijmakers JM, Weller DM, Thomashow LS, Allen AE, Paulsen IT (2012) Comparative genomics of plant-associated *Pseudomonas* spp.: insights into diversity and inheritance of traits involved in multitrophic interactions. PLoS Genet 8(7):e1002784. https://doi.org/10.1371/journal.pgen.1002784

223. Mossialos D, Ochsner U, Baysse C, Chablain P, Pirnay JP, Koedam N, Budzikiewicz H, Fernández DU, Schäfer M, Ravel J, Cornelis P (2002) Identification of new, conserved, non-ribosomal peptide synthetases from fluorescent pseudomonads involved in the biosynthesis of the siderophore pyoverdine. Mol Microbiol 45(6):1673–1685. https://doi.org/10.1046/j.1365-2958.2002.03120.x

224. Hartney SL, Mazurier S, Girard MK, Mehnaz S, Davis EW 2nd, Gross H, Lemanceau P, Loper JE (2013) Ferric-pyoverdine recognition by Fpv outer membrane proteins of *Pseudomonas protegens* Pf-5. J Bacteriol 195(4):765–776. https://doi.org/10.1128/JB.01639-12

225. Matthijs S, Brandt N, Ongena M, Achouak W, Meyer JM, Budzikiewicz H (2016) Pyoverdine and histicorrugatin-mediated iron acquisition in *Pseudomonas thivervalensis*. Biometals 29(3):467–485. https://doi.org/10.1007/s10534-016-9929-1

226. Moon CD, Zhang XX, Matthijs S, Schäfer M, Budzikiewicz H, Rainey PB (2008) Genomic, genetic and structural analysis of pyoverdine-mediated iron acquisition in the plant growth-promoting bacterium *Pseudomonas fluorescens* SBW25. BMC Microbiol 8:7. https://doi.org/10.1186/1471-2180-8-7

227. Guo W, Li F, Xia J, Wang W (2021) Complete genome sequence of a marine-derived bacterium *Pseudomonas* sp. SXM-1 and characterization of its siderophore through antiSMASH analysis and with mass spectroscopic method. Mar Genomics 55:100802. https://doi.org/10.1016/j.margen.2020.100802

228. Klapper M, Braga D, Lackner G, Herbst R, Stallforth P (2018) Bacterial alkaloid biosynthesis: structural diversity via a minimalistic nonribosomal peptide synthetase. Cell Chem Biol 25:659–665. https://doi.org/10.1016/j.chembiol.2018.02.013

229. Schimming O, Challinor VL, Tobias NJ, Adihou H, Grün P, Pöschel L, Richter C, Schwalbe H, Bode HB (2015) Structure, biosynthesis, and occurrence of bacterial pyrrolizidine alkaloids. Angew Chem Int Ed Engl 54(43):12702–12705. https://doi.org/10.1002/anie.201504877

230. Witte SNR, Hug JJ, Géraldy MNE, Müller R, Kalesse M (2017) Biosynthesis and total synthesis of pyrronazol B: a secondary metabolite from *Nannocystis pusilla*. Chemistry 23(63):15917–15921. https://doi.org/10.1002/chem.201703782.

231. Kjaerulff L, Raju R, Panter F, Scheid U, Garcia R, Herrmann J, Müller R (2017) Pyxipyrrolones: structure elucidation and biosynthesis of cytotoxic Myxobacterial metabolites. Angew Chem Int Ed Engl 56:9614–9618. https://doi.org/10.1002/anie.201704790

232. Murai Y, Mori S, Konno H, Hikichi Y, Kai K (2017) Ralstonins A and B, lipopeptides with chlamydospore-inducing and phytotoxic activities from the plant pathogen *Ralstonia solanacearum*. Org Lett 19(16):4175–4178. https://doi.org/10.1021/acs.orglett.7b01685

233. Lin XB, Lohans CT, Duar R, Zheng J, Vederas JC, Walter J, Gänzle M (2015) Genetic determinants of reutericyclin biosynthesis in *Lactobacillus reuteri*. Appl Environ Microbiol 81(6):2032–2041. https://doi.org/10.1128/AEM.03691-14

234. Gänzle MG (2004) Reutericyclin: biological activity, mode of action, and potential applications. Appl Microbiol Biotechnol 64(3):326–332. https://doi.org/10.1007/s00253-003-1536-8

235. Reimer D, Cowles KN, Proschak A, Nollmann FI, Dowling AJ, Kaiser M, ffrench-Constant R, Goodrich-Blair H, Bode HB (2013) Rhabdopeptides as insect-specific virulence factors from entomopathogenic bacteria. Chembiochem 14:1991–1997. https://doi.org/10.1002/cbic.201300205

236. Pistorius D, Müller R (2012) Discovery of the rhizopodin biosynthetic gene cluster in *Stigmatella aurantiaca* Sg a15 by genome mining. Chembiochem 13(3):416–426. https://doi.org/10.1002/cbic.201100575

237. Partida-Martinez LP, Hertweck C (2007) A gene cluster encoding rhizoxin biosynthesis in "*Burkholderia rhizoxina*", the bacterial endosymbiont of the fungus *Rhizopus microsporus*. Chembiochem 8(1):41–45. https://doi.org/10.1002/cbic.200600393

238. Brendel N, Partida-Martinez LP, Scherlach K, Hertweck C (2007) A cryptic PKS-NRPS gene locus in the plant commensal *Pseudomonas fluorescens* Pf-5 codes for the biosynthesis of an antimitotic rhizoxin complex. Org Biomol Chem 5(14):2211–2213. https://doi.org/10.1039/b707762a

239. Bosello M, Robbel L, Linne U, Xie X, Marahiel MA (2011) Biosynthesis of the siderophore rhodochelin requires the coordinated expression of three independent gene clusters in *Rhodococcus jostii* RHA1. J Am Chem So 133(12):4587–4595. https://doi.org/10.1021/ja1109453

240. Wang G, Zhao Z, Ke J, Engel Y, Shi YM, Robinson D, Bingol K, Zhang Z, Bowen B, Louie K, Wang B, Evans R, Miyamoto Y, Cheng K, Kosina S, De Raad M, Silva L, Luhrs A, Lubbe A, Hoyt DW, Francavilla C, Otani H, Deutsch S, Washton NM, Rubin EM, Mouncey NJ, Visel A, Northen T, Cheng JF, Bode HB, Yoshikuni Y (2019) CRAGE enables rapid activation of biosynthetic gene clusters in undomesticated bacteria. Nat Microbiol 4:2498–2510. https://doi.org/10.1038/s41564-019-0573-8

241. Velasco A, Acebo P, Gomez A, Schleissner C, Rodríguez P, Aparicio T, Conde S, Muñoz R, de la Calle F, Garcia JL, Sánchez-Puelles JM (2004) Molecular characterization of the safracin biosynthetic pathway from *Pseudomonas fluorescens* A2-2: designing new cytotoxic compounds. Mol Microbio 56:144–154. https://doi.org/10.1111/j.1365-2958.2004.04433.x

242. Pospiech A, Bietenhader J, Schupp T (1996) Two multifunctional peptide synthetases and an *O*-methyltransferase are involved in the biosynthesis of the DNA-binding antibiotic and antitumour agent saframycin Mx1 from *Myxococcus xanthus*. Microbiology 142:741–746. https://doi.org/10.1099/00221287-142-4-741

243. Kim GJ, Li X, Kim SH, Yang I, Hahn D, Chin J, Nam SJ, Nam JW, Nam DH, Oh DC, Chang HW, Choi H (2018) Seongsanamides A–D: antiallergic bicyclic peptides from *Bacillus safensis* KCTC 12796BP. Org Lett 20:7539–7543. https://doi.org/10.1021/acs.orglett.8b03293

244. Rosconi F, Davyt D, Martínez V, Martínez M, Abin-Carriquiry JA, Zane H, Butler A, de Souza EM, Fabiano E (2013) Identification and structural characterization of serobactins, a suite of lipopeptide siderophores produced by the grass endophyte *Herbaspirillum seropedicae*. Environ Microbiol 15:916–927. https://doi.org/10.1111/1462-2920.12075

245. Su C, Xiang Z, Liu Y, Zhao X, Sun Y, Li Z, Li L, Chang F, Chen T, Wen X, Zhou Y, Zhao F (2016) Analysis of the genomic sequences and metabolites of *Serratia surfactantfaciens* sp. nov. YD25^{T} that

simultaneously produces prodigiosin and serrawettin W2. BMC Genomics 17(1):865. https://doi.org/10.1186/s12864-016-3171-7

246. Flury P, Vesga P, Péchy-Tarr M, Aellen N, Dennert F, Hofer N, Kupferschmied KP, Kupferschmied P, Metla Z, Ma Z, Siegfried S, de Weert S, Bloemberg G, Höfte M, Keel CJ, Maurhofer M (2017) Antimicrobial and insecticidal: cyclic lipopeptides and hydrogen cyanide produced by plant-beneficial *Pseudomonas* strains CHA0, CMR12a, and PCL1391 contribute to insect killing. Front Microbiol 8:100. https://doi.org/10.3389/fmicb.2017.00100

247. Garcia-Gonzalez E, Müller S, Ensle P, Süssmuth RD, Genersch E (2014) Elucidation of sevadicin, a novel non-ribosomal peptide secondary metabolite produced by the honey bee pathogenic bacterium *Paenibacillus larvae*. Environ Microbiol 16(5):1297–309. https://doi.org/10.1111/1462-2920.12417

248. Potharla VY, Wang C, Cheng YQ (2014) Identification and characterization of the spiruchostatin biosynthetic gene cluster enable yield improvement by overexpressing a transcriptional activator. J Ind Microbiol Biotechnol 41(9):1457–1465. https://doi.org/10.1007/s10295-014-1474-8

249. Marner M, Patras MA, Kurz M, Zubeil F, Förster F, Schuler S, Bauer A, Hammann P, Vilcinskas A, Schäberle TF, Glaeser J (2020) Molecular networking-guided discovery and characterization of stechlisins, a group of cyclic lipopeptides from a *Pseudomonas* sp. J Nat Prod 83(9):2607–2617. https://doi.org/10.1021/acs.jnatprod.0c00263

250. Li R, Oliver RA, Townsend CA (2017) Identification and characterization of the sulfazecin monobactam biosynthetic gene cluster. Cell Chem Bio 24:24–34. https://doi.org/10.1016/j.chembiol.2016.11.010

251. Götze S, Arp J, Lackner G, Zhang S, Kries H, Klapper M, García-Altares M, Willing K, Günther M, Stallforth P (2019) Structure elucidation of the syringafactin lipopeptides provides insight in the evolution of nonribosomal peptide synthetases. Chem Sci 10(48):10979–10990.

https://doi.org/10.1039/c9sc03633d

252. Amrein H, Makart S, Granado J, Shakya R, Schneider-Pokorny J, Dudler R (2004) Functional analysis of genes involved in the synthesis of syringolin A by *Pseudomonas syringae* pv. *syringae* B301 D-R. Mol Plant Microbe Interact 17(1):90–97. https://doi.org/10.1094/MPMI.2004.17.1.90

253. Guenzi E, Galli G, Grgurina I, Gross DC, Grandi G (1998) Characterization of the syringomycin synthetase gene cluster. A link between prokaryotic and eukaryotic peptide synthetases. J Biol Chem 273:32857–3263. https://doi.org/10.1074/jbc.273.49.32857

254. Grgurina I, Mariotti F, Fogliano V, Gallo M, Scaloni A, Iacobellis NS, Lo Cantore P, Mannina L, van Axel Castelli V, Greco ML, Graniti A (2002) A new syringopeptin produced by bean strains of *Pseudomonas syringae* pv. *syringae*. Biochim Biophys Acta 1597(1):81–89. https://doi.org/10.1016/s0167-4838(02)00283-2

255. Scholz-Schroeder BK, Soule JD, Gross DC (2003) The sypA, sypS, and sypC synthetase genes encode twenty-two modules involved in the nonribosomal peptide synthesis of syringopeptin by *Pseudomonas syringae* pv. *syringae* B301D. Mol Plant Microbe Interact 16(4):271–80. https://doi.org/10.1094/MPMI.2003.16.4.271

256. Ravindran A, Jalan N, Yuan JS, Wang N, Gross DC (2015) Comparative genomics of *Pseudomonas syringae* pv. *syringae* strains B301D and HS191 and insights into intrapathovar traits associated with plant pathogenesis. Microbiologyopen 4(4):553–573. https://doi.org/10.1002/mbo3.261

257. Kreutzer MF, Nett M (2012) Genomics-driven discovery of taiwachelin, a lipopeptide siderophore from *Cupriavidus taiwanensis*. Org Biomol Chem 10(47):9338–9343. https://doi.org/10.1039/c2ob26296g

258. Desjardine K, Pereira A, Wright H, Matainaho T, Kelly M, Andersen RJ (2007) Tauramamide, a lipopeptide antibiotic produced in culture by *Brevibacillus laterosporus* isolated from a marine habitat:

structure elucidation and synthesis. J Nat Prod 70(12):1850–1853. https://doi.org/10.1021/np070209r

259. Kronenwerth M, Bozhüyük KA, Kahnt AS, Steinhilber D, Gaudriault S, Kaiser M, Bode HB (2014) Characterisation of taxlllaids A–G; natural products from *Xenorhabdus indica*. Chemistry 20(52):17478–17487. https://doi.org/10.1002/chem.201403979

260. Guo C, Mandalapu D, Ji X, Gao J, Zhang Q (2018) Chemistry and biology of teixobactin. Chem Eur J 24(21):5406–5422. https://doi.org/10.1002/chem.201704167.

261. Wozniak CE, Lin Z, Schmidt EW, Hughes KT, Liou TG (2018) Thailandamide, a fatty acid Synthesis antibiotic that is coexpressed with a resistant target gene. Antimicrob Agents Chemother 62:e00463-18. https://doi.org/10.1128/AAC.00463-18

262. Liu X, Biswas S, Berg MG, Antapli CM, Xie F, Wang Q, Tang MC, Tang GL, Zhang L, Dreyfuss G, Cheng YQ (2013) Genomics-guided discovery of thailanstatins A, B, and C as pre-mRNA splicing inhibitors and antiproliferative agents from *Burkholderia thailandensis* MSMB43. J Nat Prod 76:685–693. https://doi.org/10.1021/np300913h

263. Ross AC, Xu Y, Lu L, Kersten RD, Shao Z, Al-Suwailem AM, Dorrestein PC, Qian PY, Moore BS (2013) Biosynthetic multitasking facilitates thalassospiramide structural diversity in marine bacteria. J Am Chem Soc 135(3):1155–1162. https://doi.org/10.1021/ja3119674

264. Kirchner N, Cano-Prieto C, Schulz-Fincke AC, Gütschow M, Ortlieb N, Moschny J, Niedermeyer THJ, Horak J, Lämmerhofer M, van der Voort M, Raaijmakers JM, Gross H (2021) Discovery of thanafactin A, a linear, proline-containing octalipopeptide from *Pseudomonas* sp. SH-C52, motivated by genome mining. J Nat Prod 84:101–109. https://doi.org/10.1021/acs.jnatprod.0c01174

265. Watrous J, Roach P, Alexandrov T, Heath BS, Yang JY, Kersten RD, van der Voort M, Pogliano K, Gross H, Raaijmakers JM, Moore BS, Laskin J, Bandeira N, Dorrestein PC (2012) Mass spectral molecular networking of living microbial colonies. Proc Natl Acad Sci USA 109:E1743–E1752.

https://doi.org/10.1073/pnas.1203689109

266. Mendes R, Kruijt M, de Bruijn I, Dekkers E, van der Voort M, Schneider JH, Piceno YM, DeSantis TZ, Andersen GL, Bakker PA, Raaijmakers JM (2011) Deciphering the rhizosphere microbiome for disease-suppressive bacteria. Science 332(6033):1097–1100. https://doi.org/10.1126/science.1203980

267. Van Der Voort M, Meijer HJ, Schmidt Y, Watrous J, Dekkers E, Mendes R, Dorrestein PC, Gross H, Raaijmakers JM (2015) Genome mining and metabolic profiling of the rhizosphere bacterium *Pseudomonas* sp. SH-C52 for antimicrobial compounds. Front Microbiol 6:693. https://doi.org/10.3389/fmicb.2015.00693

268. Oueis E, Klefisch T, Zaburannyi N, Garcia R, Plaza A, Müller R (2019) Two biosynthetic pathways in *Jahnella thaxteri* for thaxteramides, distinct types of lipopeptides. Org Lett 21(14):5407–5412. https://doi.org/10.1021/acs.orglett.9b01524

269. Murphy AC, Gao SS, Han LC, Carobene S , Fukuda D, Song Z, Hothersall J, Cox RJ, Crosby J, Crump MP, Thomas CM,. Willis L, Simpson TJ (2014) Biosynthesis of thiomarinol a and related metabolites of *Pseudoalteromonas* sp. SANK 73390. Chem Sci 5:397–402. https://doi.org/10.1039/C3SC52281D

270. Buntin K, Irschik H, Weissman KJ, Luxenburger E, Blöcker H, Müller R (2010) Biosynthesis of thuggacins in myxobacteria: comparative cluster analysis reveals basis for natural product structural diversity. Chem Biol 17(4):342–56. https://doi.org/10.1016/j.chembiol.2010.02.013

271. Wolff H, Bode HB (2018) The benzodiazepine-like natural product tilivalline is produced by the entomopathogenic bacterium *Xenorhabdus eapokensis*. PLoS One 13:e0194297. https://doi.org/10.1371/journal.pone.0194297

272. Scherlach K, Lackner G, Graupner K, Pidot S, Bretschneider T, Hertweck C (2013) Biosynthesis and mass spectrometric imaging of tolaasin, the virulence factor of brown blotch mushroom disease.

Chembiochem 14(18):2439–2443. https://doi.org/10.1002/cbic.201300553

273. Lohans CT, Huang Z, van Belkum MJ, Giroud M, Sit CS, Steels EM, Zheng J, Whittal RM, McMullen LM, Vederas JC (2012) Structural characterization of the highly cyclized lantibiotic paenicidin A via a partial desulfurization/reduction strategy. J Am Chem Soc 134:19540–19543. https://doi.org/10.1021/ja3089229

274. Sandmann A, Sasse F, Müller R (2004) Identification and analysis of the core biosynthetic machinery of tubulysin, a potent cytotoxin with potential anticancer activity. Chem Biol 11:1071–1079. https://doi.org/ 10.1016/j.chembiol.2004.05.014

275. Han AW, Sandy M, Fishman B, Trindade-Silva AE, Soares CA, Distel DL, Butler A, Haygood MG (2013) Turnerbactin, a novel triscatecholate siderophore from the shipworm endosymbiont *Teredinibacter turnerae* T7901. PLoS One 8(10):e76151. https://doi.org/10.1371/journal.pone.0076151

276. Spathelf BM, Rautenbach M (2009) Anti-listerial activity and structure-activity relationships of the six major tyrocidines, cyclic decapeptides from *Bacillus aneurinolyticus*. Bioorg Med Chem 17(15):5541-5548. https://doi.org/10.1016/j.bmc.2009.06.029

277. Komaki H, Hosoyama A, Ichikaw N, Igarashi Y (2016) Draft genome sequence of a sponge-derived *Brevibacillus* sp. TP-B0800, a producer of ulbactins with tumor cell migration inhibitory activity. Gene Reports 6:140–143. https://doi.org/10.1016/j.genrep.2016.10.006.

278. Johnston CW, Skinnider MA, Wyatt MA, Li X, Ranieri MR, Yang L, Zechel DL, Ma B, Magarvey NA (2015) An automated genomes-to-natural products platform (GNP) for the discovery of modular natural products. Nat Commun 6:8421. https://doi.org/10.1038/ncomms9421

279. Balado M, Osorio CR, Lemos ML (2006) A gene cluster involved in the biosynthesis of vanchrobactin, a chromosome-encoded siderophore produced by *Vibrio anguillarum*. Microbiology

(Reading) 152(Pt 12):3517–3528. https://doi.org/10.1099/mic.0.29298-0

280. Kurth C, Schieferdecker S, Athanasopoulou K, Seccareccia I, Nett M (2016) Variochelins, lipopeptide siderophores from *Variovorax boronicumulans* discovered by genome mining. J Nat Prod 79(4):865–872. https://doi.org/10.1021/acs.jnatprod.5b00932

281. Keating TA, Marshall CG, Walsh CT (2000) Reconstitution and characterization of the *Vibrio cholerae* vibriobactin synthetase from VibB, VibE, VibF, and VibH. Biochemistry 39:15522–15530. https://doi.org/ 10.1021/bi0016523

282. González V, Santamaría RI, Bustos P, Hernández-González I, Medrano-Soto A, Moreno-Hagelsieb G, Janga SC, Ramírez MA, Jiménez-Jacinto V, Collado-Vides J, Dávila G (2006) The partitioned *Rhizobium etli* genome: genetic and metabolic redundancy in seven interacting replicons. Proc Natl Acad Sci U S A 103(10):3834–3839. https://doi.org/10.1073/pnas.0508502103

283. Yan F, Auerbach D, Chai Y, Keller L, Tu Q, Hüttel S, Glemser A, Grab HA, Bach T, Zhang Y, Müller R (2018) Biosynthesis and heterologous production of vioprolides: rational biosynthetic engineering and unprecedented 4-methylazetidinecarboxylic acid formation. Angew Chem Int Ed Engl 57:8754–8759. https://doi.org/10.1002/anie.201802479

284. Bonnichsen L, Bygvraa Svenningsen N, Rybtke M, de Bruijn I, Raaijmakers JM, Tolker-Nielsen T, Nybroe O (2015) Lipopeptide biosurfactant viscosin enhances dispersal of *Pseudomonas fluorescens* SBW25 biofilms. Microbiology (Reading) 161(12):2289–2297. https://doi.org/10.1099/mic.0.000191

285. Götze S, Arp J, Lackner G, Zhang S, Kries H, Klapper M, García-Altares M, Willing K, Günther M, Stallforth P (2019) Structure elucidation of the syringafactin lipopeptides provides insight in the evolution of nonribosomal peptide synthetases. Chem Sci 10(48):10979–10990. https://doi.org/10.1039/c9sc03633d

286. Zhang W, Li Y, Qian G, Wang Y, Chen H, Li YZ, Liu F, Shen Y, Du L (2011) Identification and

characterization of the anti-methicillin-resistant *Staphylococcus aureus* WAP-8294A2 biosynthetic gene cluster from *Lysobacter enzymogenes* OH11. Antimicrob Agents Chemother 55(12):5581–5589. https://doi.org/10.1128/AAC.05370-11

287. Sang M, Wang H, Shen Y, Rodrigues de Almeida N, Conda-Sheridan M, Li S, Li Y, Du L (2019) Identification of an anti-MRSA cyclic lipodepsipeptide, WBP-29479A1, by genome mining of *Lysobacter antibioticus*. Org Lett 21(16):6432–6436. https://doi.org/10.1021/acs.orglett.9b02333
288. Rokni-Zadeh H, Li W, Sanchez-Rodriguez A, Sinnaeve D, Rozenski J, Martins JC, De Mot R (2012) Genetic and functional characterization of cyclic lipopeptide white-line-inducing principle (WLIP) production by rice rhizosphere isolate *Pseudomonas putida* RW10S2. Appl Environ Microbiol 78(14):4826–4834. https://doi.org/10.1128/AEM.00335-12
289. Li W, Rokni-Zadeh H, De Vleeschouwer M, Ghequire MG, Sinnaeve D, Xie GL, Rozenski J, Madder A, Martins JC, De Mot R (2013) The antimicrobial compound xantholysin defines a new group of *Pseudomonas* cyclic lipopeptides. PLoS One 8:e62946. https://doi.org/10.1371/journal.pone.0062946
290. Kegler C, Bode HB (2020) Artificial splitting of a non-ribosomal peptide synthetase by inserting natural docking domains. Angew Chem Int Ed Engl 59:13463–13467. https://doi.org/10.1002/anie.201915989
291. Tobias NJ, Wolff H, Djahanschiri B, Grundmann F, Kronenwerth M, Shi YM, Simonyi S, Grün P, Shapiro-Ilan D, Pidot SJ, Stinear TP, Ebersberger I, Bode HB (2017) Natural product diversity associated with the nematode symbionts *Photorhabdus* and *Xenorhabdus*. Nat Microbiol 2:1676–1685. https://doi.org/10.1038/s41564-017-0039-9
292. Crawford JM, Portmann C, Kontnik R, Walsh CT, Clardy J (2011) NRPS substrate promiscuity diversifies the xenematides. Org Lett 13:5144–5147. https://doi.org/10.1021/ol2020237

293. Zhou Q, Grundmann F, Kaiser M, Schiell M, Gaudriault S, Batzer A, Kurz M, Bode HB (2013) Structure and biosynthesis of xenoamicins from entomopathogenic *Xenorhabdus*. Chemistry 19:16772–16779. https://doi.org/10.1002/chem.201302481

294. Park D, Ciezki K, van der Hoeven R, Singh S, Reimer D, Bode HB, Forst S (2009) Genetic analysis of xenocoumacin antibiotic production in the mutualistic bacterium *Xenorhabdus nematophila*. Mol Microbiol 73(5):938–949. https://doi.org/10.1111/j.1365-2958.2009.06817.x

295. Lang G, Kalvelage T, Peters A, Wiese J, Imhoff JF (2008) Linear and cyclic peptides from the entomopathogenic bacterium *Xenorhabdus nematophilus*. J Nat Prod 71(6):1074–1077. https://doi.org/10.1021/np800053n

296. Kegler C, Nollmann FI, Ahrendt T, Fleischhacker F, Bode E, Bode HB (2014) Rapid determination of the amino acid configuration of xenotetrapeptide. Chembiochem 15:826–828. https://doi.org/10.1002/ cbic. 201300602

297. Zhou Q, Dowling A, Heide H, Wöhnert J, Brandt U, Baum J, Ffrench-Constant R, Bode HB (2012) Xentrivalpeptides A–Q: depsipeptide diversification in Xenorhabdus. J Nat Prod 75(10):1717–1722. https://doi.org/10.1021/np300279g

298. Pfeifer BA, Wang CC, Walsh CT, Khosla C (2003) Biosynthesis of yersiniabactin, a complex polyketide-nonribosomal peptide, using *Escherichia coli* as a heterologous host. Appl Environ Microbiol 69(11):6698–702. https://doi.org/10.1128/AEM.69.11.6698-6702.2003

299. Taniguchi M, Suzumura K, Nagai K, Kawasaki T, Takasaki J, Sekiguchi M, Moritani Y, Saito T, Hayashi K, Fujita S, Tsukamoto S, Suzuki K (2004) YM-254890 analogues, novel cyclic depsipeptides with $G\alpha_{q/11}$ inhibitory activity from *Chromobacterium* sp. QS3666. Bioorg Med Chem 12(12):3125–3133. https://doi.org/10.1016/j.bmc.2004.04.006

300. Kevany BM, Rasko DA, Thomas MG (2009) Characterization of the complete zwittermicin A

biosynthesis gene cluster from *Bacillus cereus*. Appl Environ Microbiol 75:1144–1155. https://doi.org/10.1128/AEM.02518-08

301. Borgio JF, Alhujaily R, Alquwaie R, Alabdullah MJ, AlHasani E, Alothman W, Alaqeel RK, Alfaraj AS, Kaabi A, Alhur NF, Akhtar S, AlJindan R, Almofty S, Almandil NB, AbdulAzeez S (2023) Mining the nanotube-forming *Bacillus amyloliquefaciens* MR14M3 genome for determining anti-*Candida auris* and anti-*Candida albicans* potential by pathogenicity and comparative genomics analysis. Comput Struct Biotechnol J 21:4261-4276. https://doi.org/10.1016/j.csbj.2023.08.031.

302. Wang Z, Zhang W, Wang Z, Zhang Z, Liu Y, Liu S, Wu Q, Saiding E, Han J, Zhou J, Xu J, Yi X, Zhang Z, Wang R, Su X (2024) Analysis of antimicrobial biological activity of a marine *Bacillus velezensis* NDB. Arch Microbiol 206 (3)**:**131. https://doi.org/10.1007/s00203-024-03861-4.

303. Borgio JF, Alhujaily R, Alfaraj AS, Alabdullah MJ, Alaqeel RK, Kaabi A, Alquwaie R, Alhur NF, AlJindan R, Almofty S, Almohazey D, Natarajan A, Dhas TS, AbdulAzeez S, Almandil NB (2024) Genome-guided identification of surfactin-producing *Bacillus halotolerans* AQ11M9 with anti-*Candida auris* potential. Int J Mol Sci 25(19)**:**10408. https://doi.org/10.3390/ijms251910408.

304. Ashajyothi M, Mahadevakumar S, Venkatesh YN, Sarma PVSRN, Danteswari C, Balamurugan A, Prakash G, Khandelwal V, Tarasatyavathi C, Podile AR, Mysore KS, Chandranayaka S (2024) Comprehensive genomic analysis of *Bacillus subtilis* and *Bacillus paralicheniformis* associated with the pearl millet panicle reveals their antimicrobial potential against important plant pathogens. BMC Plant Biol 24:197. https://doi.org/10.1186/s12870-024-04975-z.

305. Wu D, Fu L, Cao Y, Dong N, Li D (2023) Genomic insights into antimicrobial potential and optimization of fermentation conditions of pig-derived *Bacillus subtilis* BS21. Front Microbiol 14:1239837. https://doi.org/10.3389/fmicb.2023.1239837.

306. Meesil W, Muangpat P, Sitthisak S, Rattanarojpong T, Chantratita N, Machado RAR, Shi YM, Bode

HB, Vitta A, Thanwisai A (2023) Genome mining reveals novel biosynthetic gene clusters in entomopathogenic bacteria. Sci Rep 13:20764. https://doi.org/10.1038/s41598-023-47121-9.
